# Supplementary material for: Lipid peroxidation and type I interferon coupling fuels pathogenic macrophage activation causing tuberculosis susceptibility
Source: eLife. 2025 Oct 2;14:RP106814. doi: 10.7554/eLife.106814 (PMC12490860; doi:10.7554/eLife.106814)

Figure 2A: Nrf2

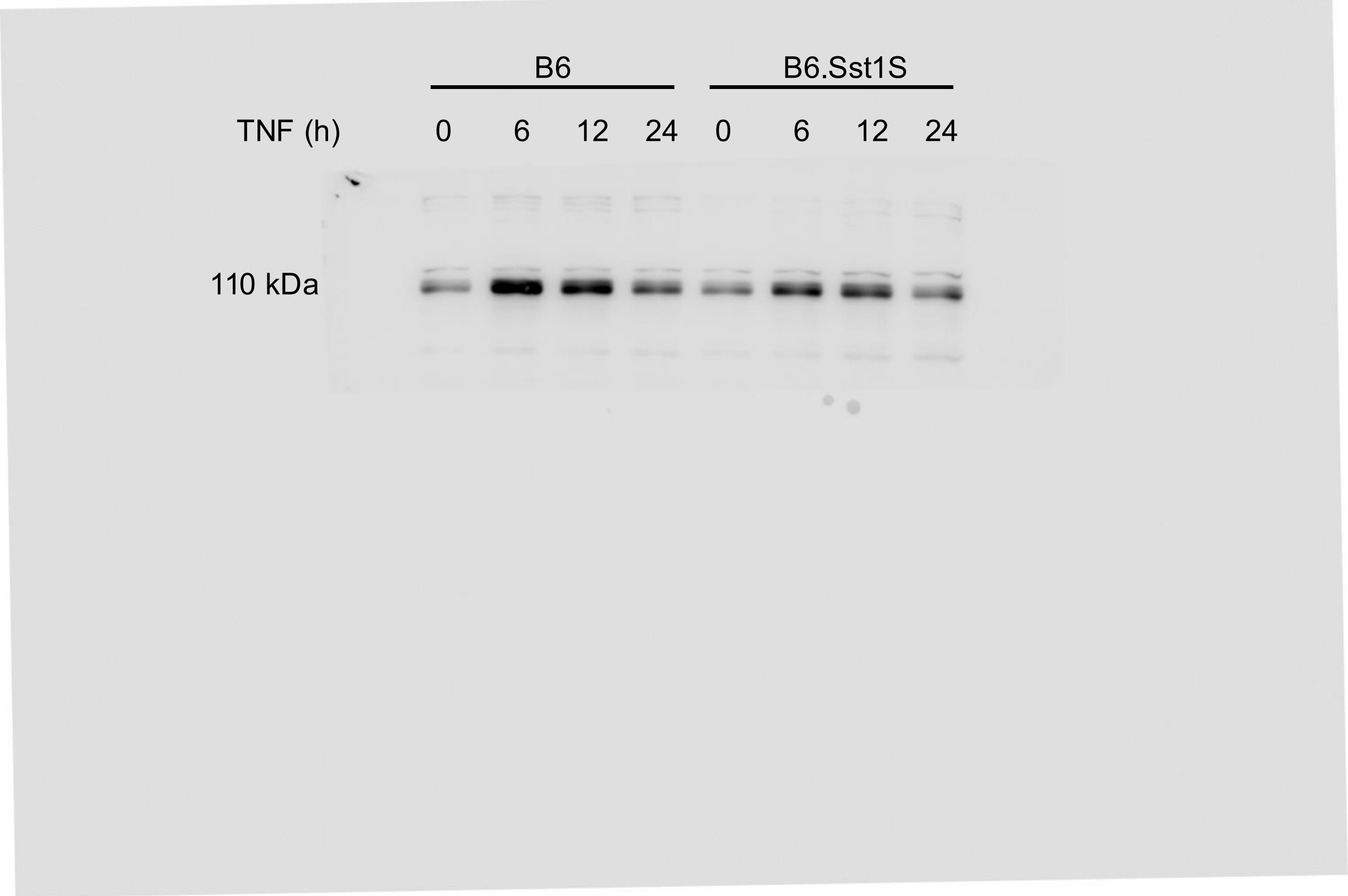

Figure 2A: β-tubulin

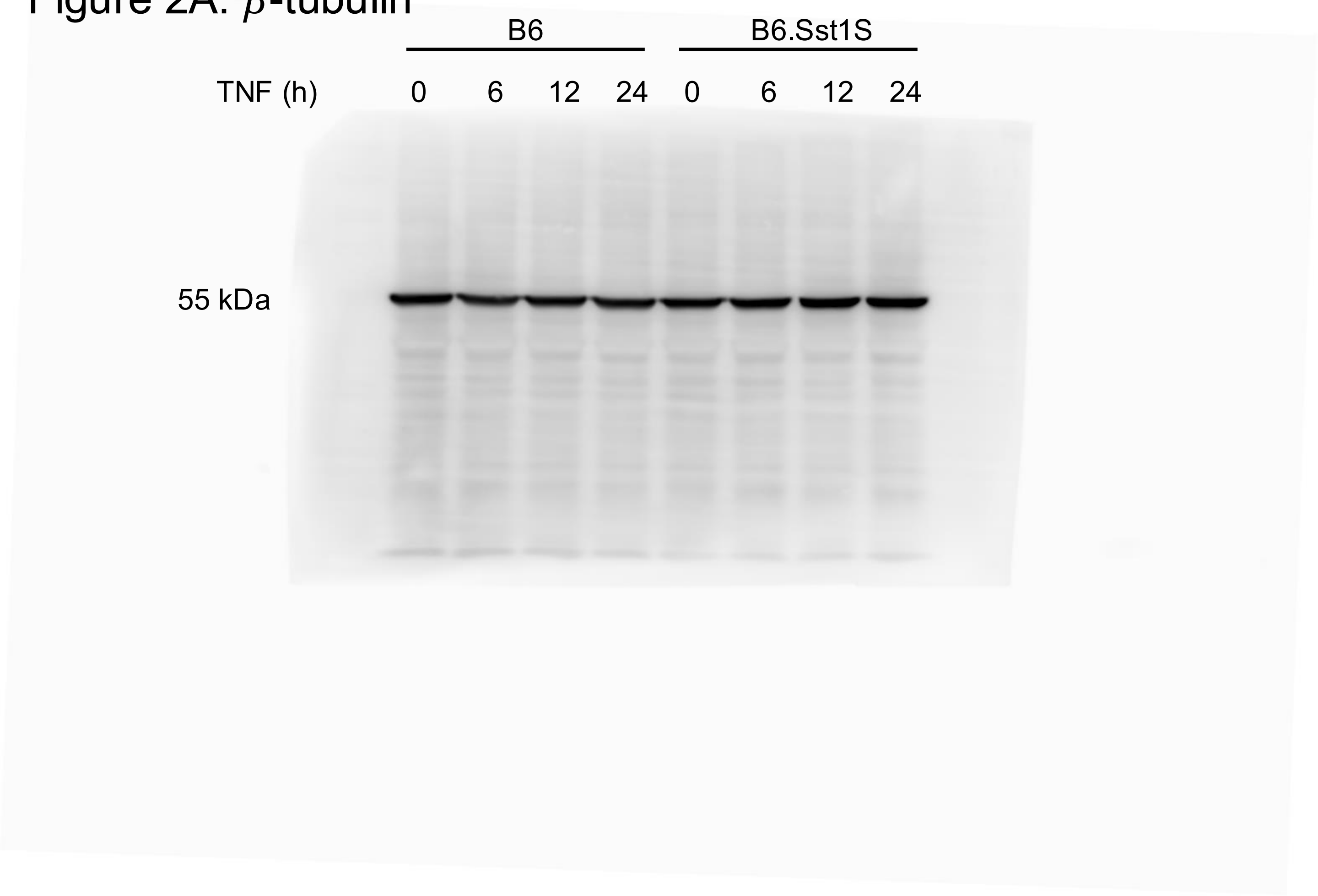

Figure 2B and 2C: Bach1

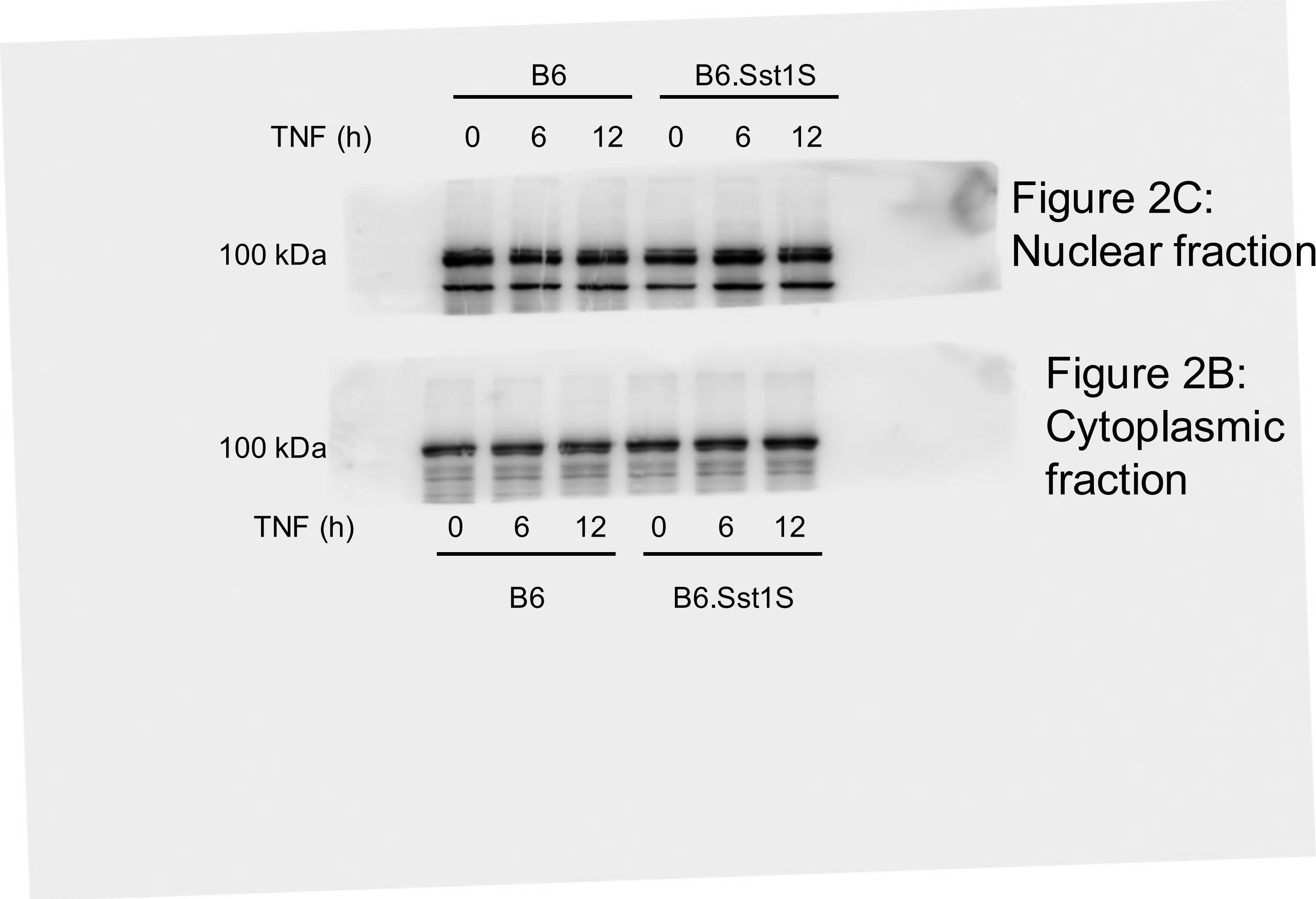

Figure 2B and 2C: Nrf2

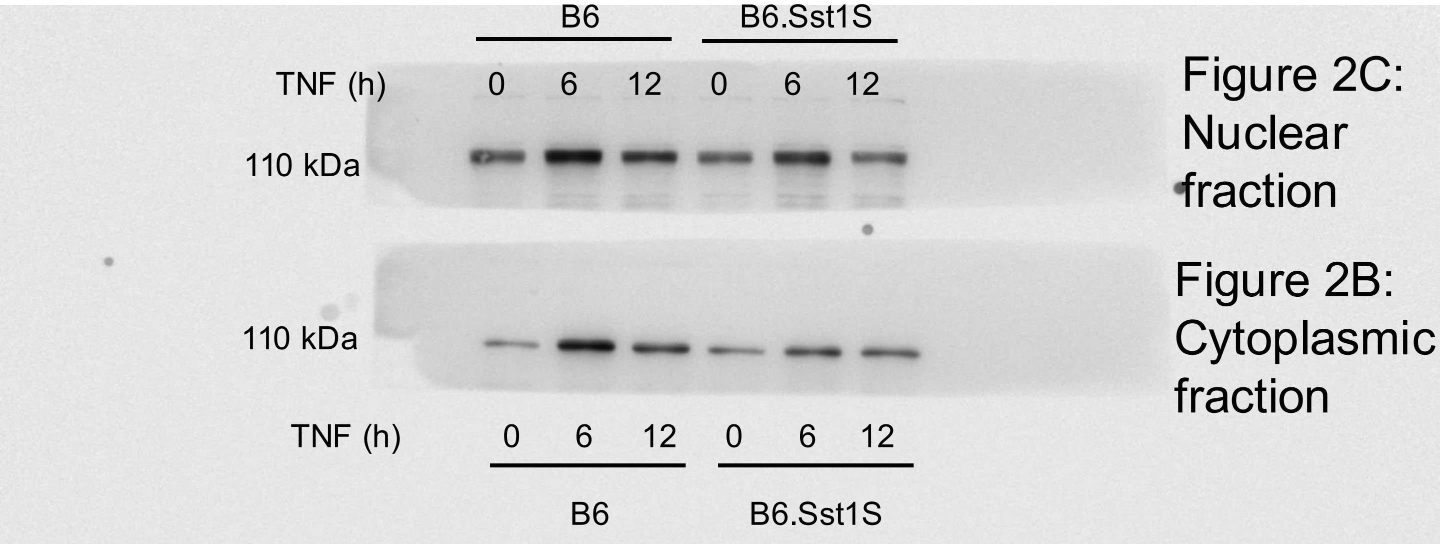

Figure 2B: β-tubulin

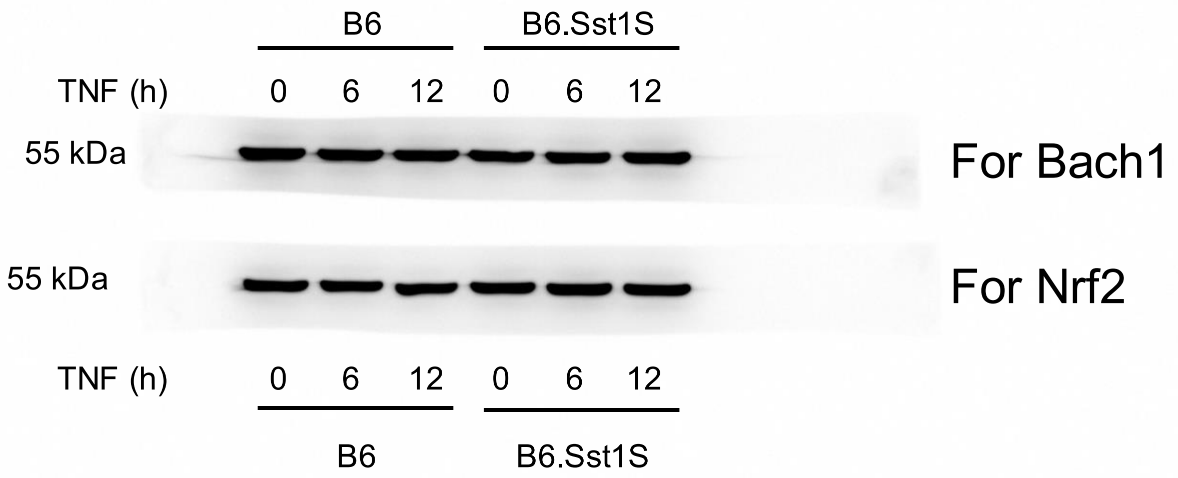

Figure 2C: Histone H3

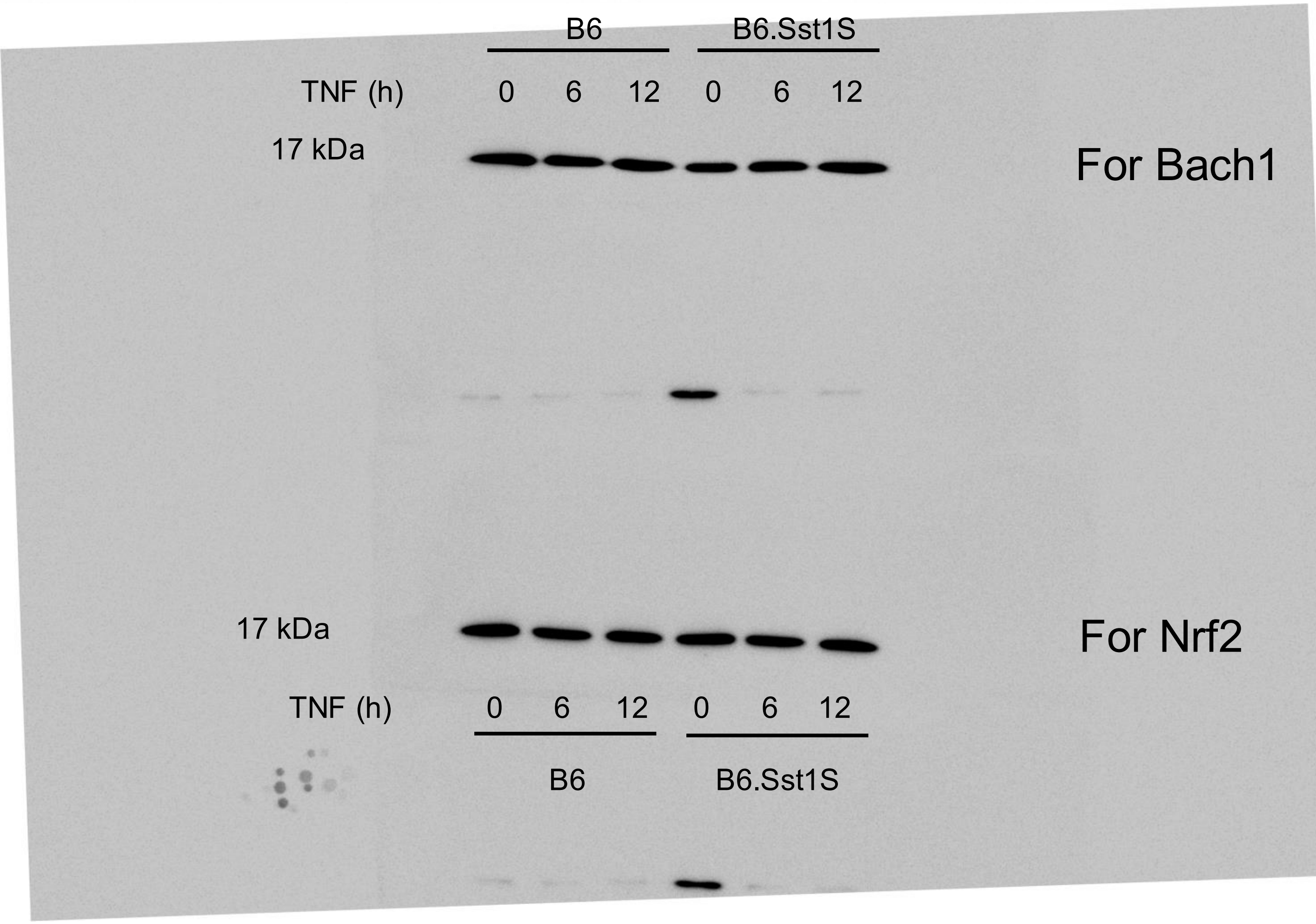

Figure 2H: Nrf2

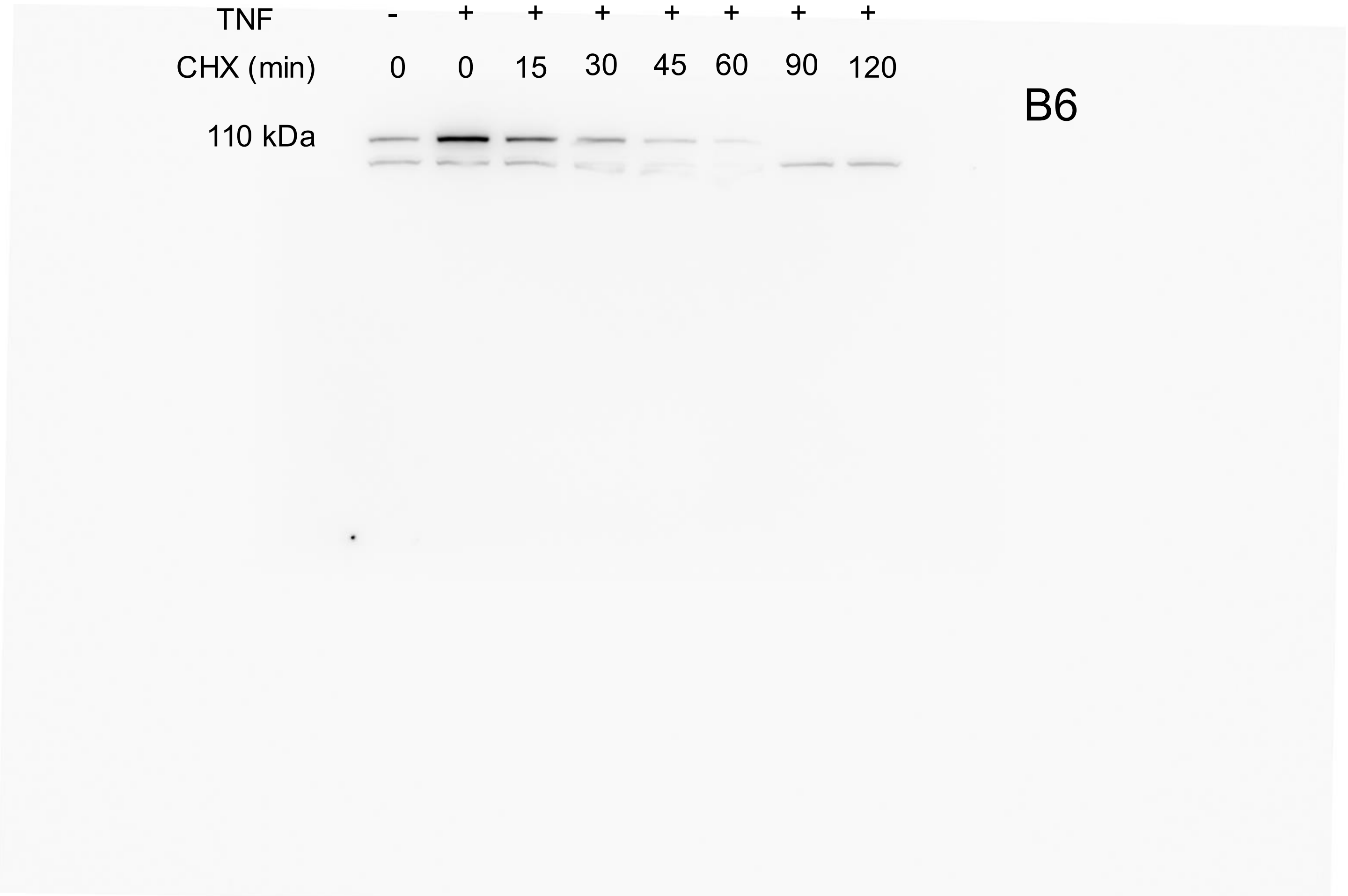

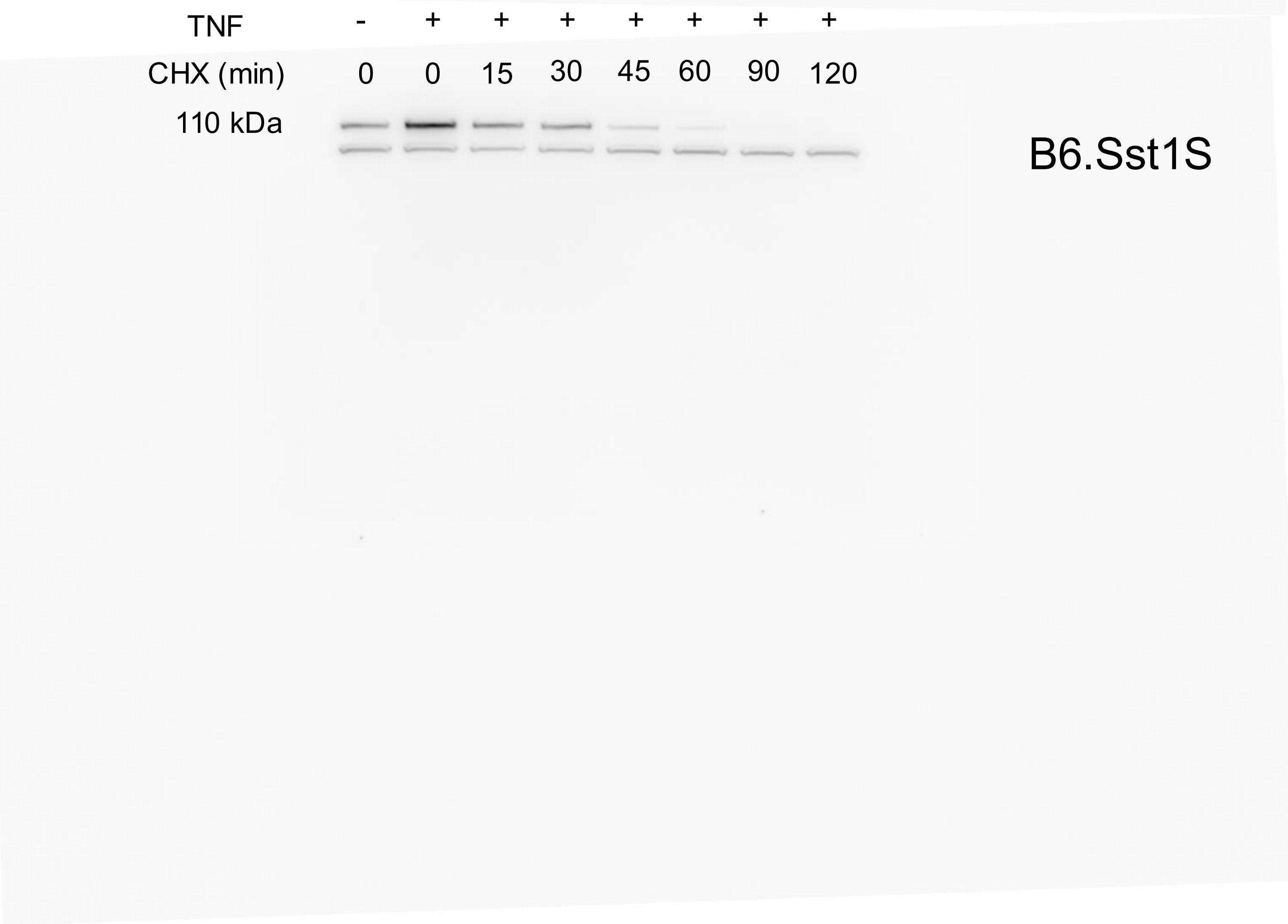

Figure 2H: β-tubulin

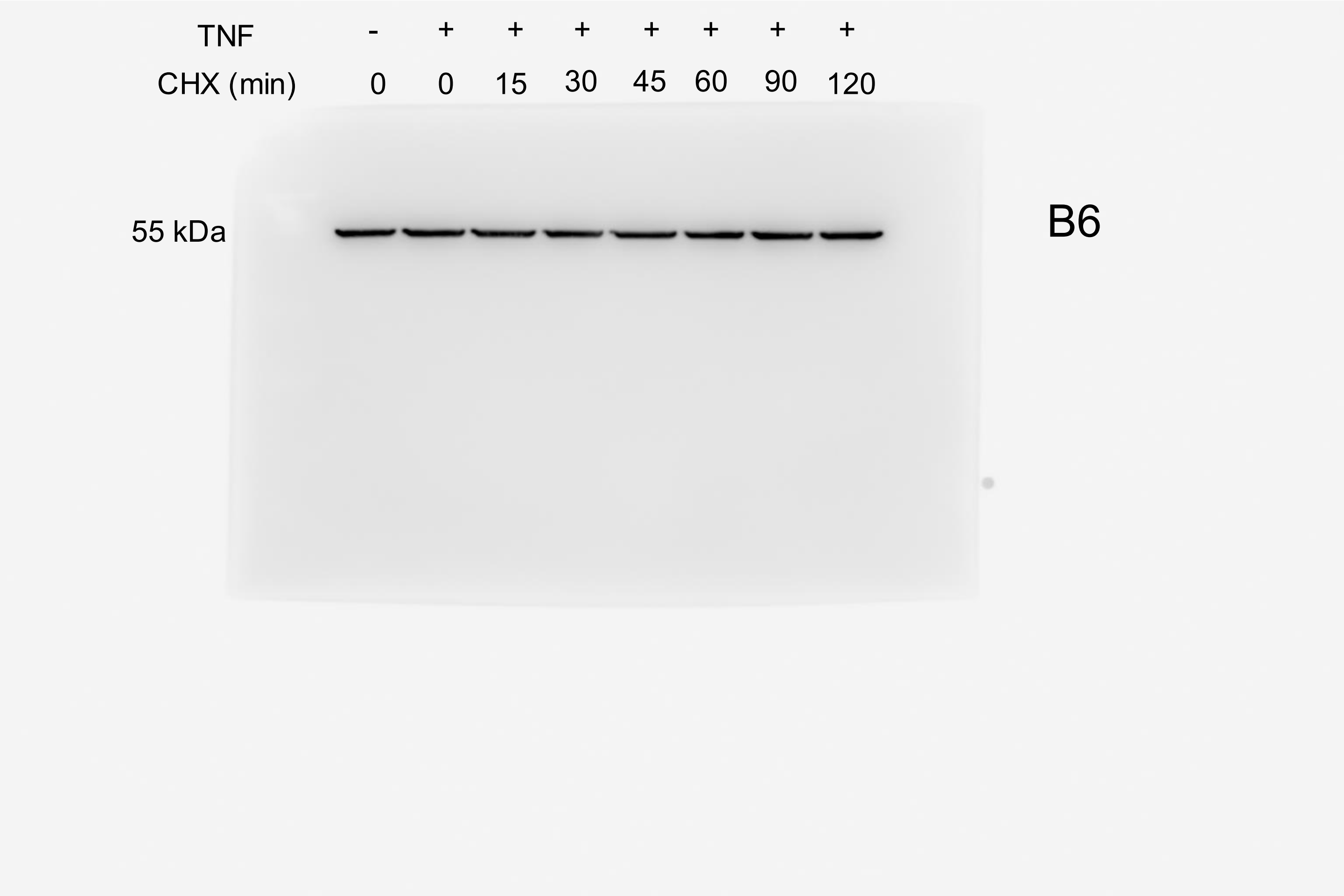

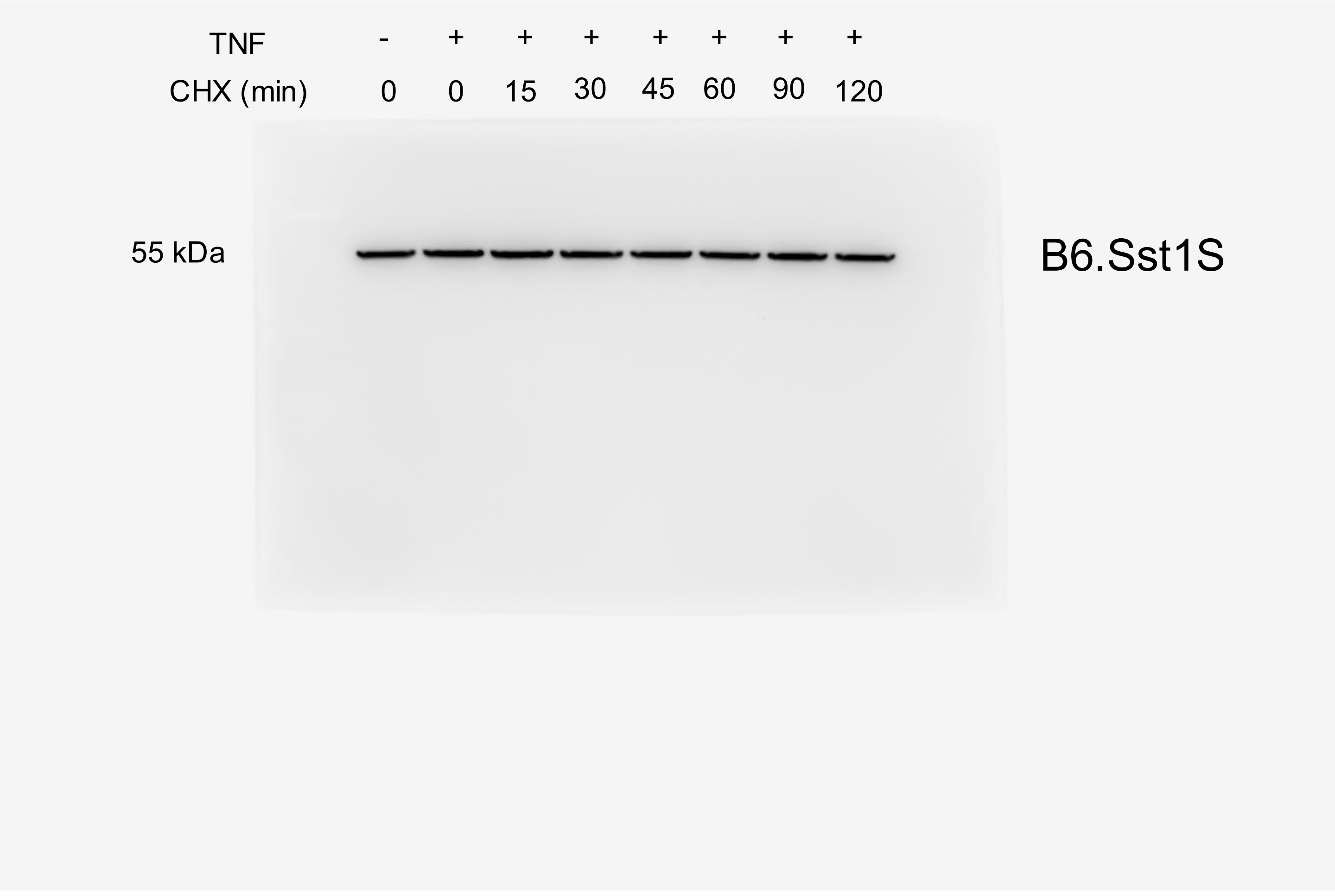

Figure 2I: EMSA

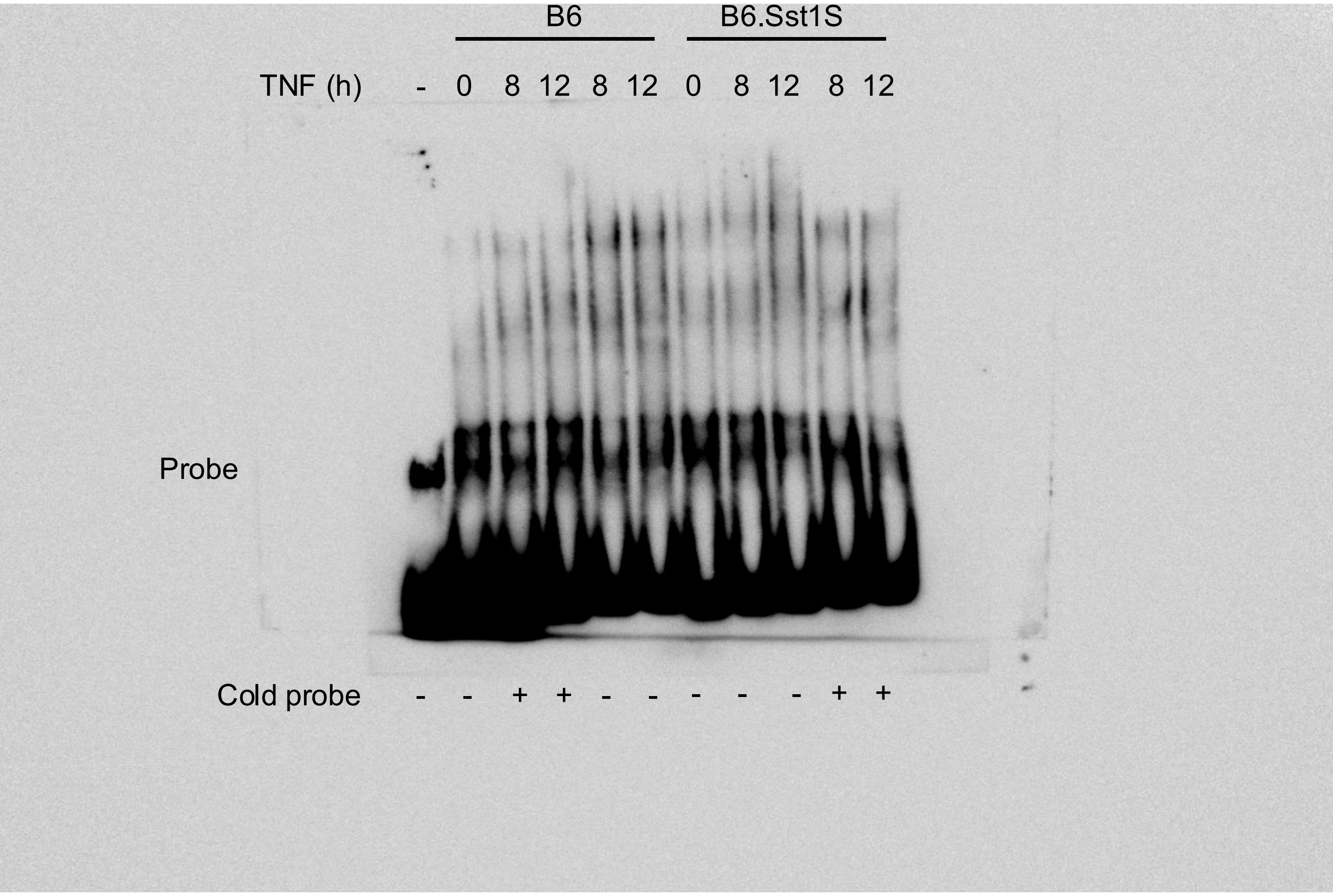

Supplement: Figure 2—source data 1. [file elife-106814-fig2-data1.zip › Figure 2-source data 1.pdf]
