# Supplementary figures and images for: Lipid peroxidation and type I interferon coupling fuels pathogenic macrophage activation causing tuberculosis susceptibility
Source: eLife. 2025 Oct 2;14:RP106814. doi: 10.7554/eLife.106814 (PMC12490860; doi:10.7554/eLife.106814)

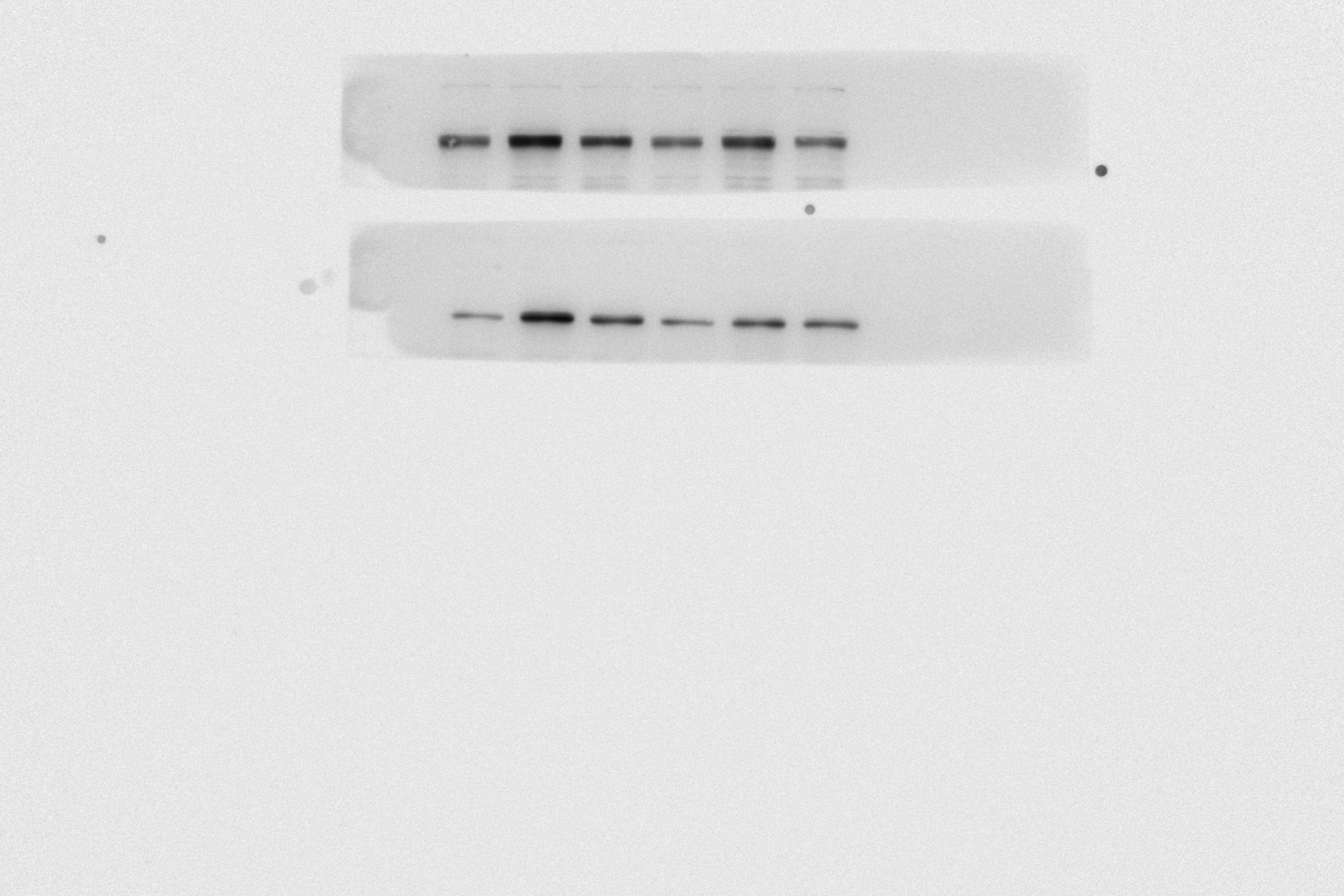

Supplement: Figure 2—source data 2. [file elife-106814-fig2-data2.zip › Figure 2-source data 2/Figure 2B and 2C_Nrf2.tif]

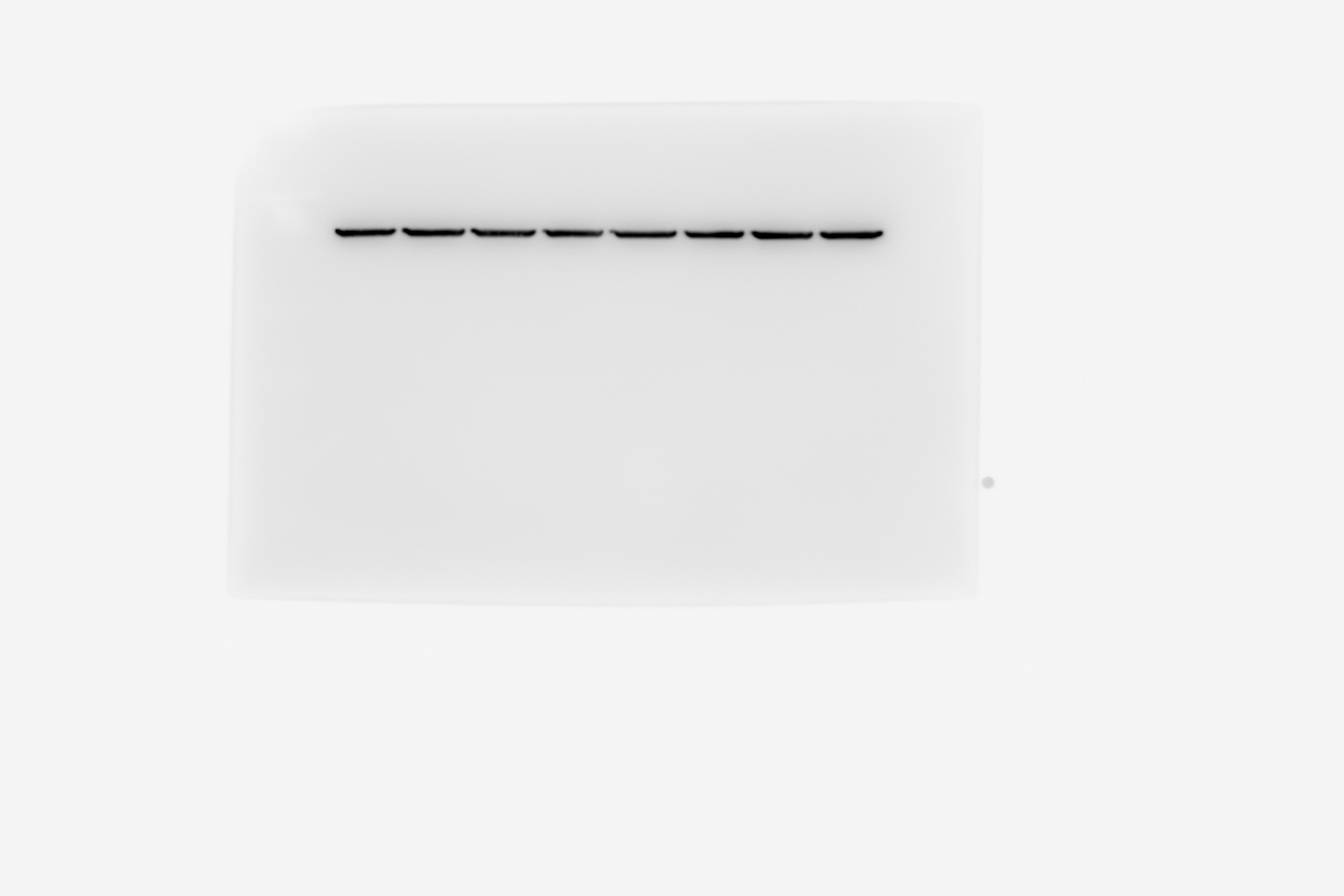

Supplement: Figure 2—source data 2. [file elife-106814-fig2-data2.zip › Figure 2-source data 2/Figure 2H_b-tubulin (for B6).tif]

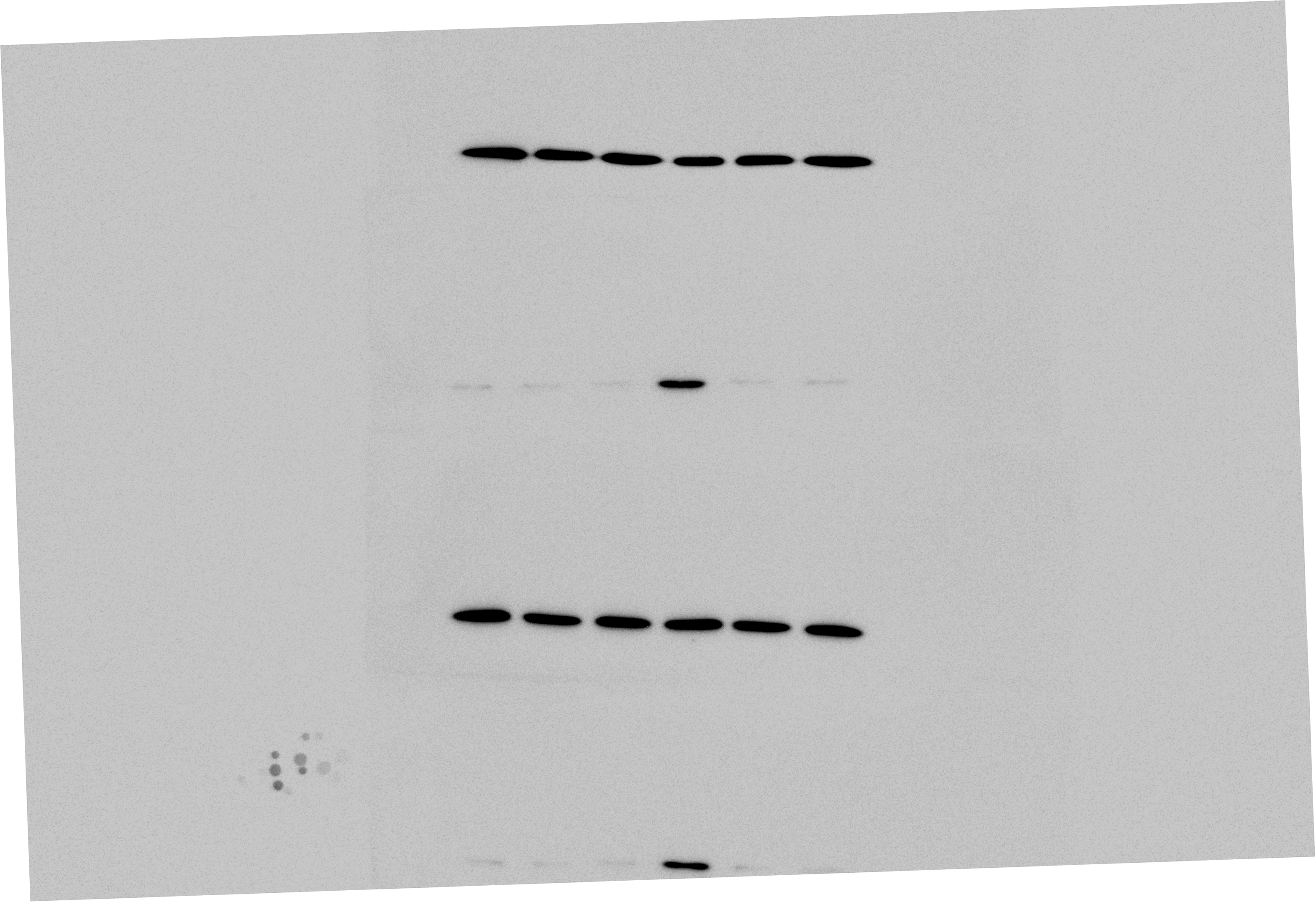

Supplement: Figure 2—source data 2. [file elife-106814-fig2-data2.zip › Figure 2-source data 2/Figure 2C_Histone H3.tif]

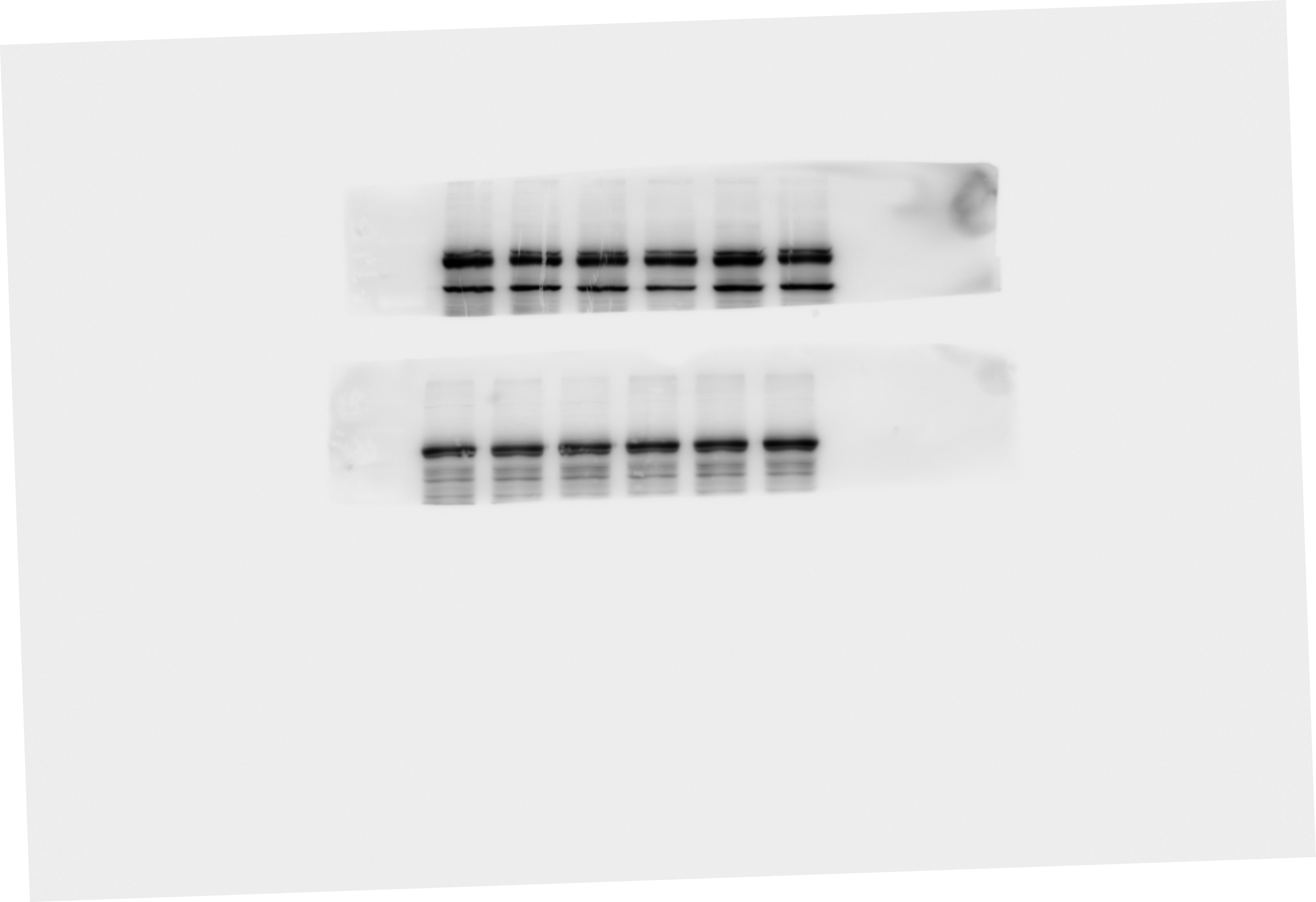

Supplement: Figure 2—source data 2. [file elife-106814-fig2-data2.zip › Figure 2-source data 2/Figure 2_2B and C_Bach1.tif]

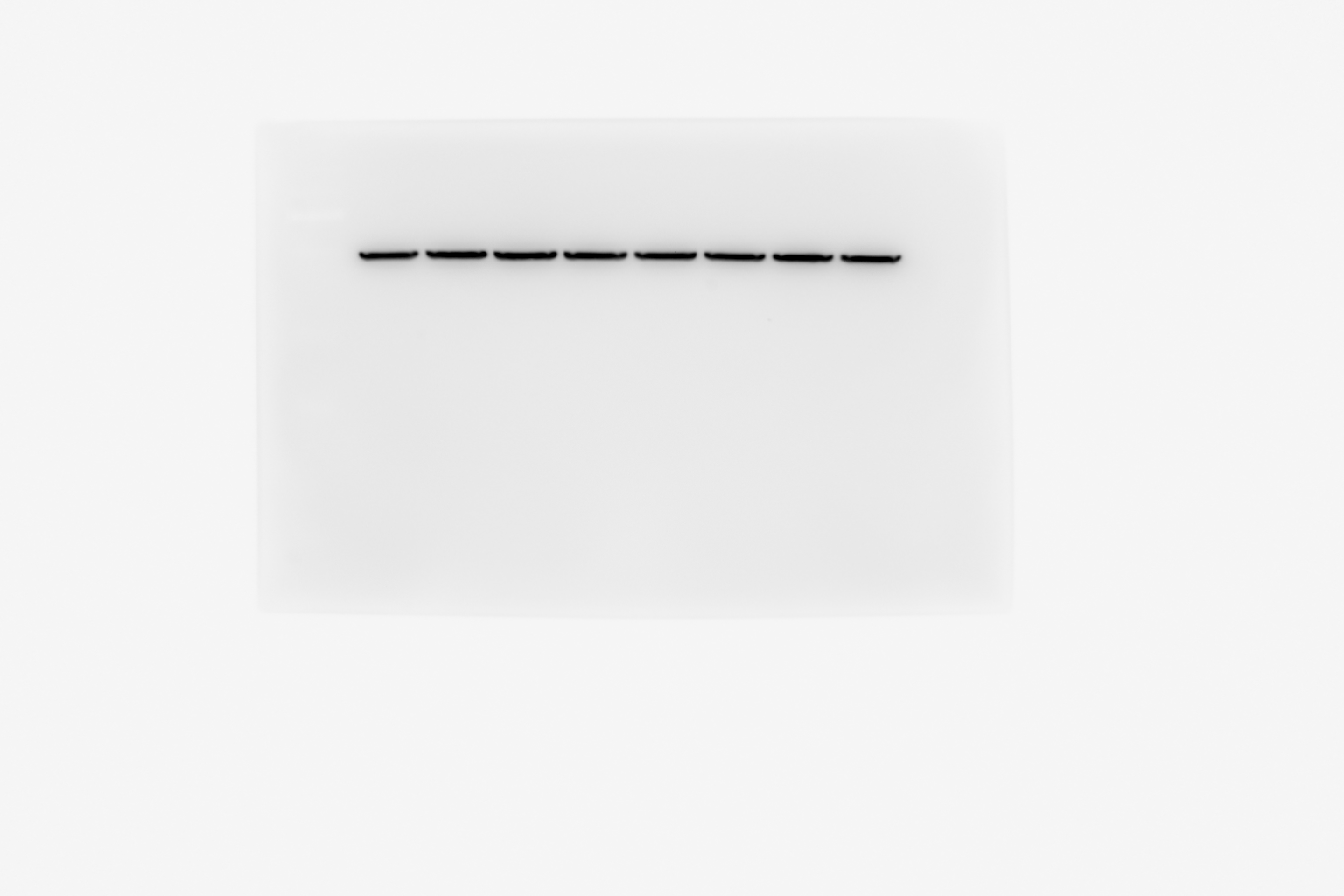

Supplement: Figure 2—source data 2. [file elife-106814-fig2-data2.zip › Figure 2-source data 2/Figure 2H_b-tubulin (for B6.Sst1S).tif]

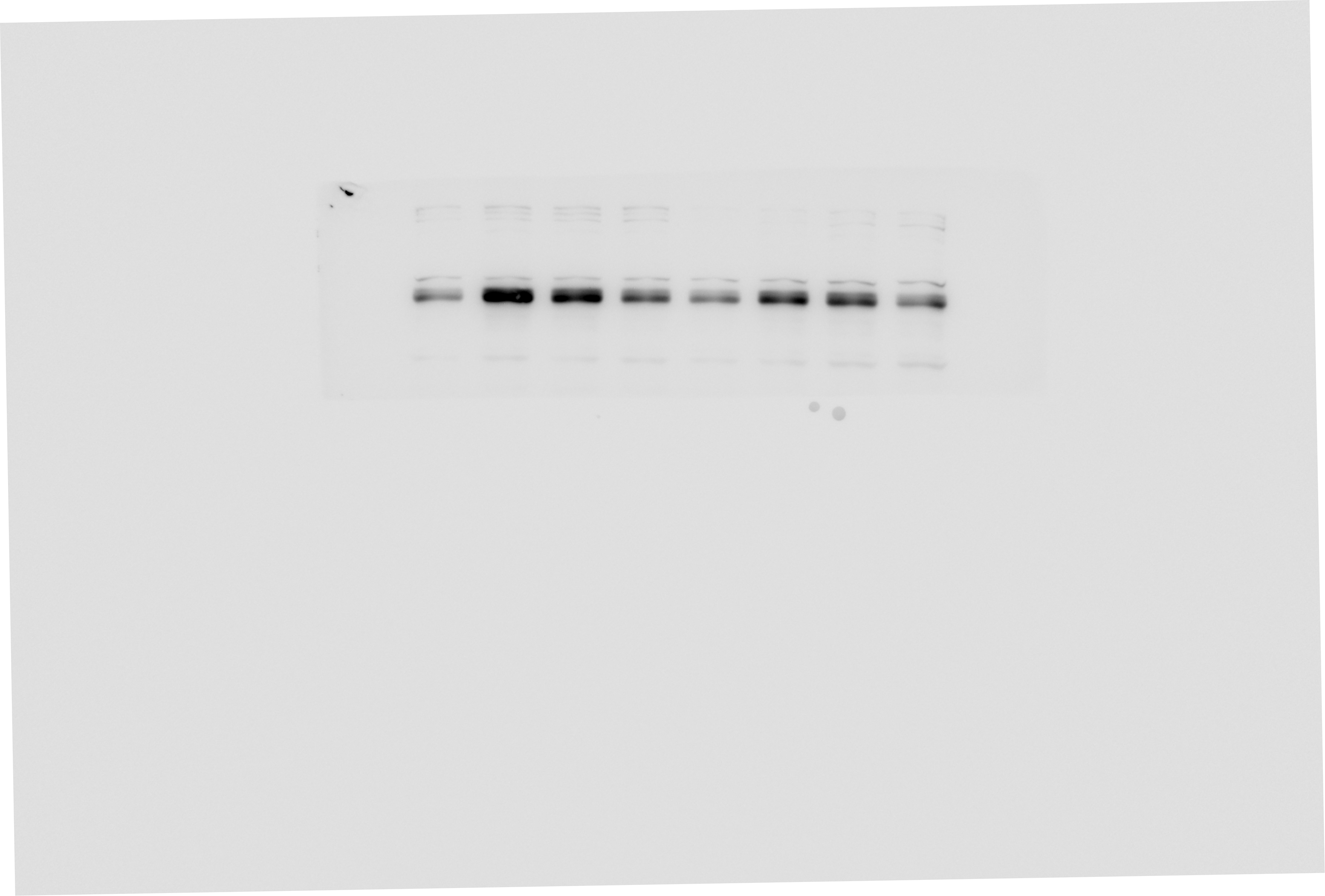

Supplement: Figure 2—source data 2. [file elife-106814-fig2-data2.zip › Figure 2-source data 2/Figure 2A_Nrf2.tif]

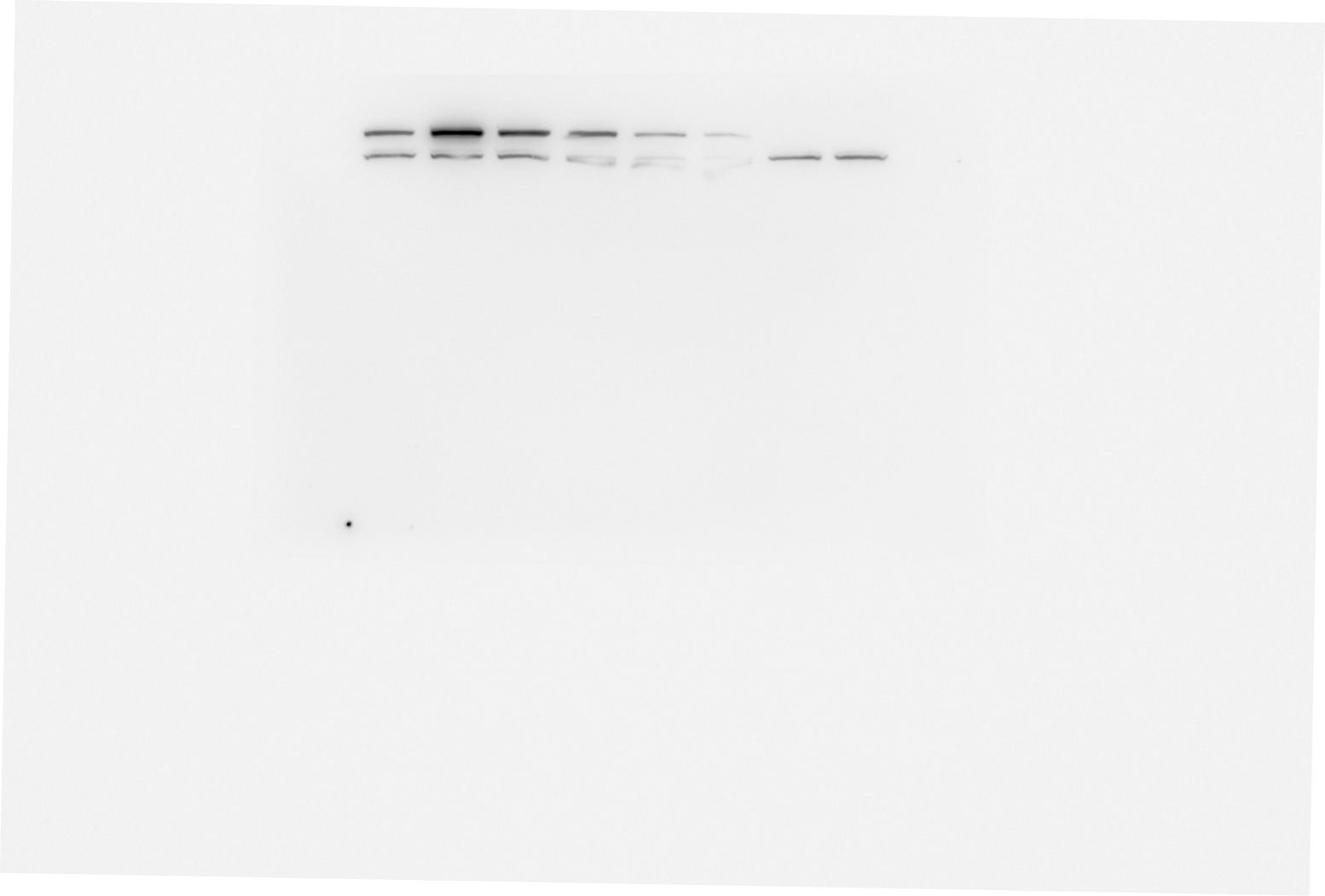

Supplement: Figure 2—source data 2. [file elife-106814-fig2-data2.zip › Figure 2-source data 2/Figure 2H_Nrf2 (for B6).tif]

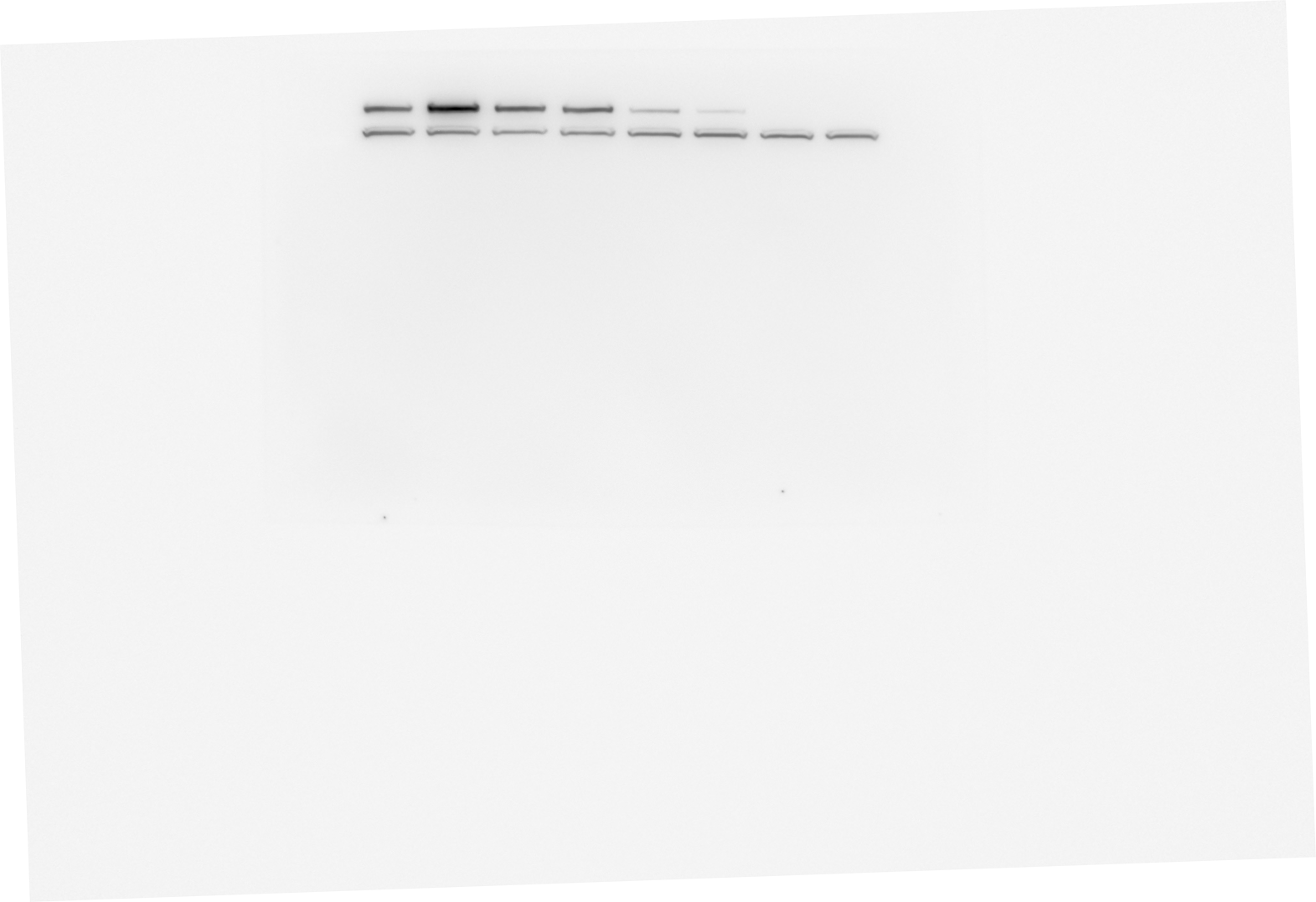

Supplement: Figure 2—source data 2. [file elife-106814-fig2-data2.zip › Figure 2-source data 2/Figure 2H_Nrf2 (B6.Sst1S).tif]

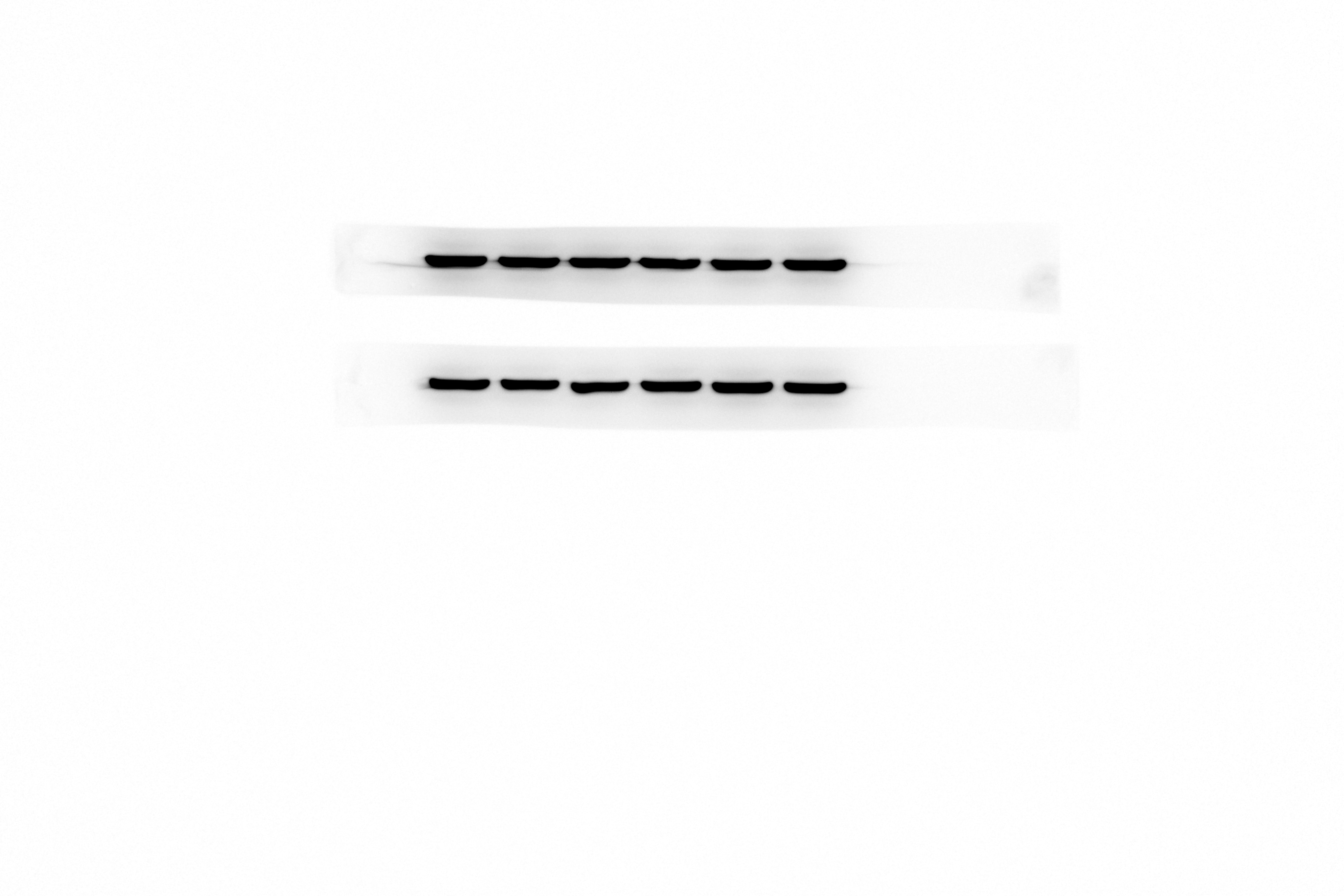

Supplement: Figure 2—source data 2. [file elife-106814-fig2-data2.zip › Figure 2-source data 2/Figure 2B_b-tubulin.tif]

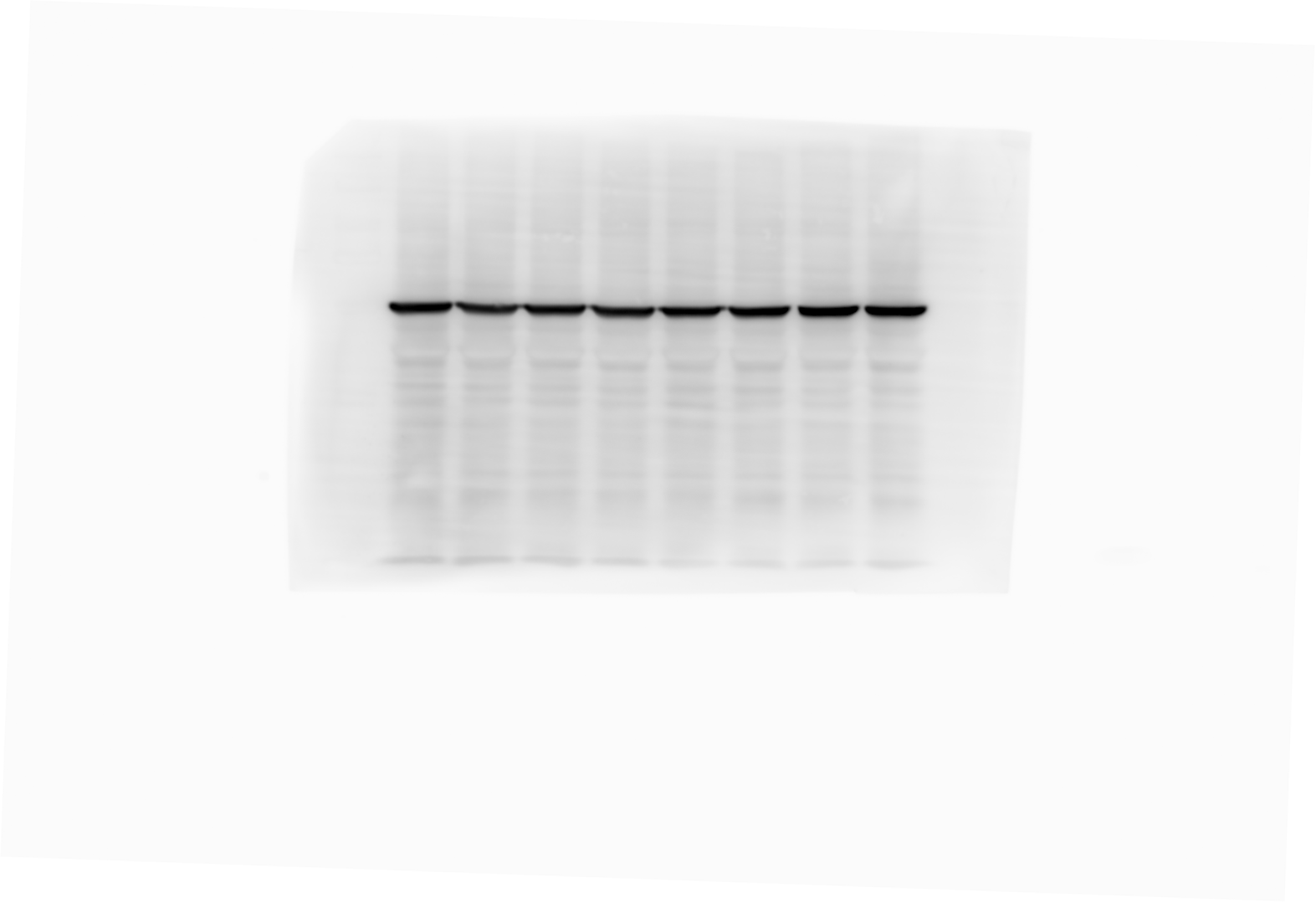

Supplement: Figure 2—source data 2. [file elife-106814-fig2-data2.zip › Figure 2-source data 2/Figure 2A_b-tubulin.tif]

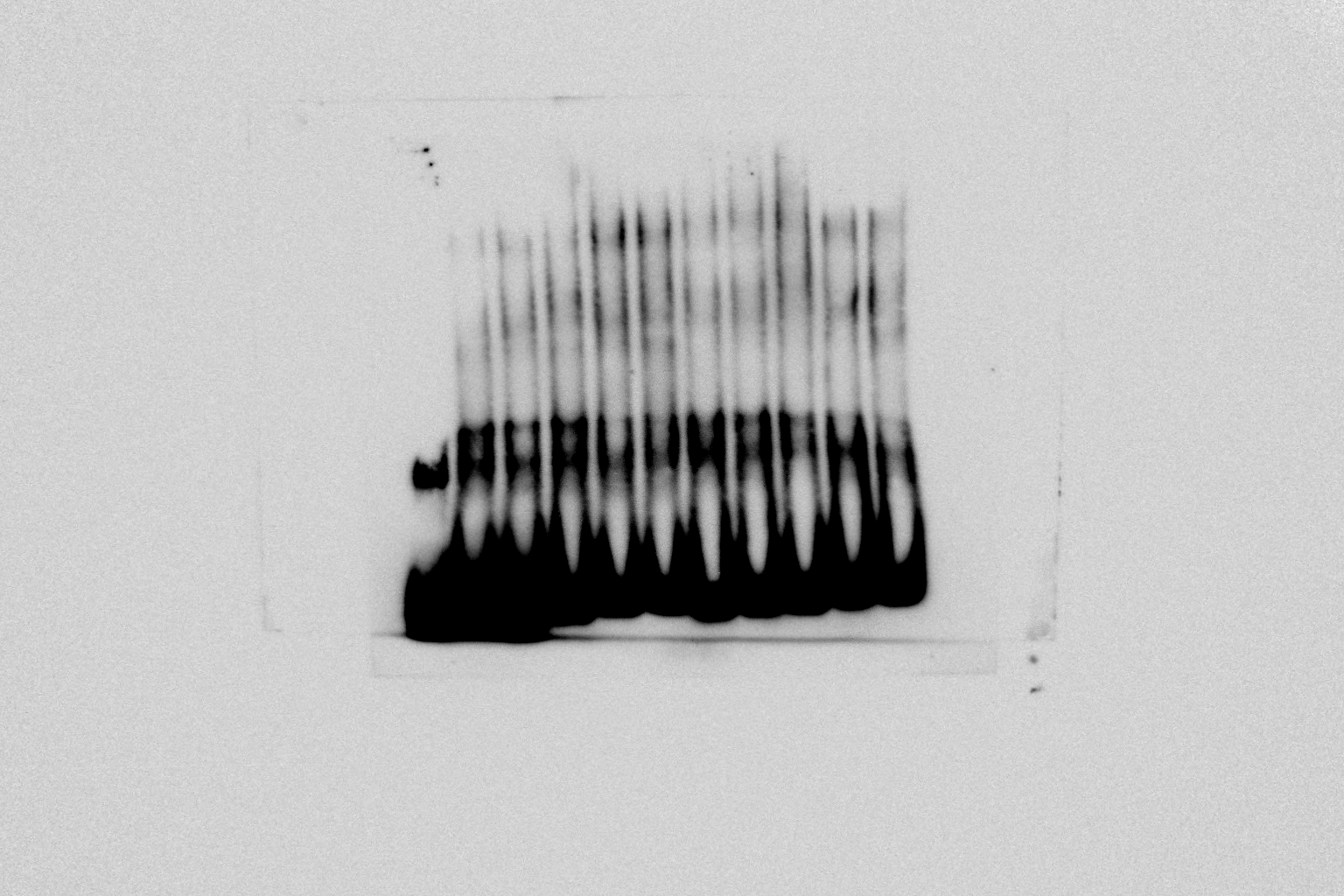

Supplement: Figure 2—source data 2. [file elife-106814-fig2-data2.zip › Figure 2-source data 2/Figure 2I_EMSA.tif]

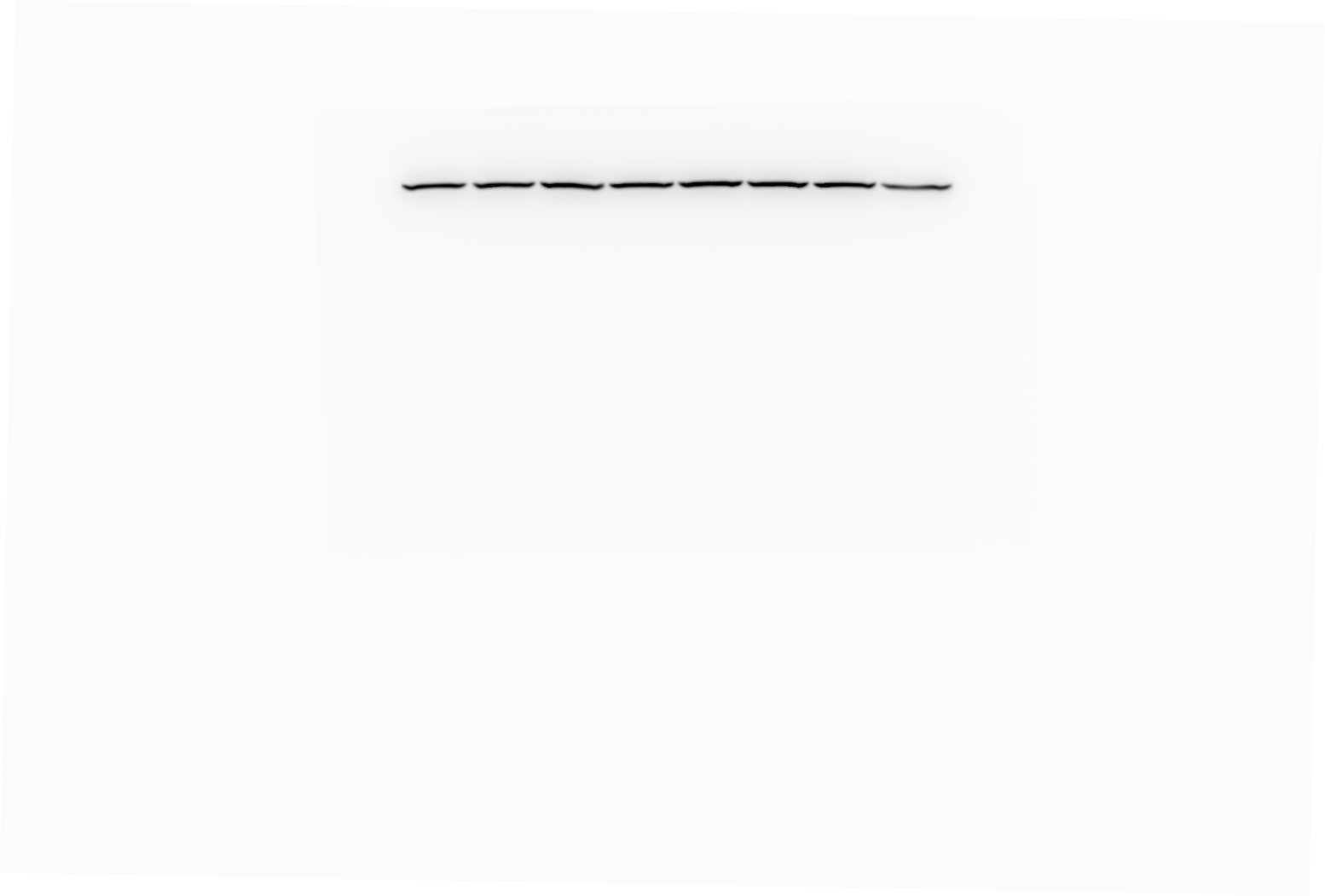

Supplement: Figure 2—figure supplement 1—source data 2. [file elife-106814-fig2-figsupp1-data2.zip › Figure 2-figure suppplement 1-source data 2/Figure 2-figure supplement 1A_b-TrCP.tif]

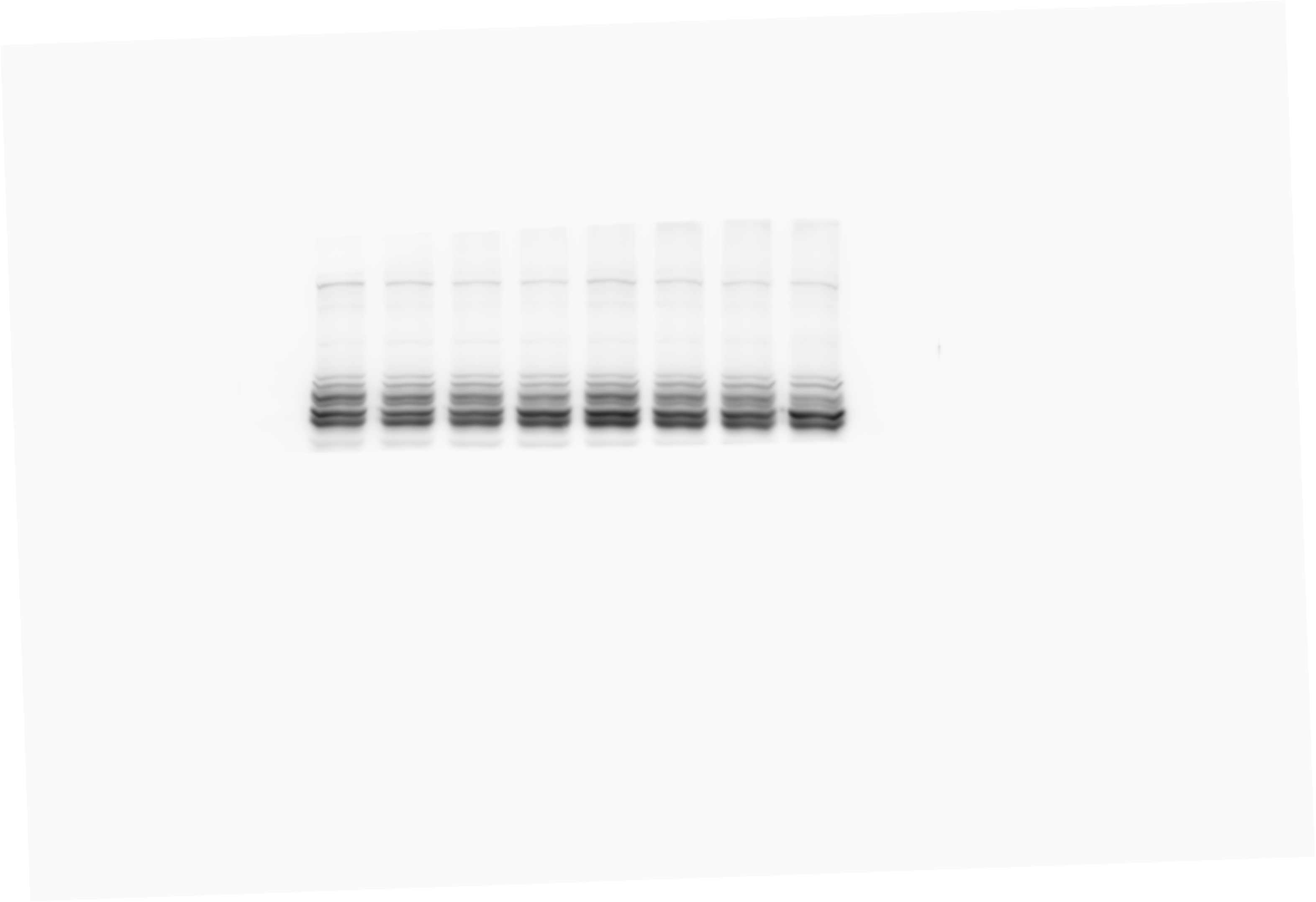

Supplement: Figure 2—figure supplement 1—source data 2. [file elife-106814-fig2-figsupp1-data2.zip › Figure 2-figure suppplement 1-source data 2/Figure 2-figure supplement 1A_Keap1.tif]

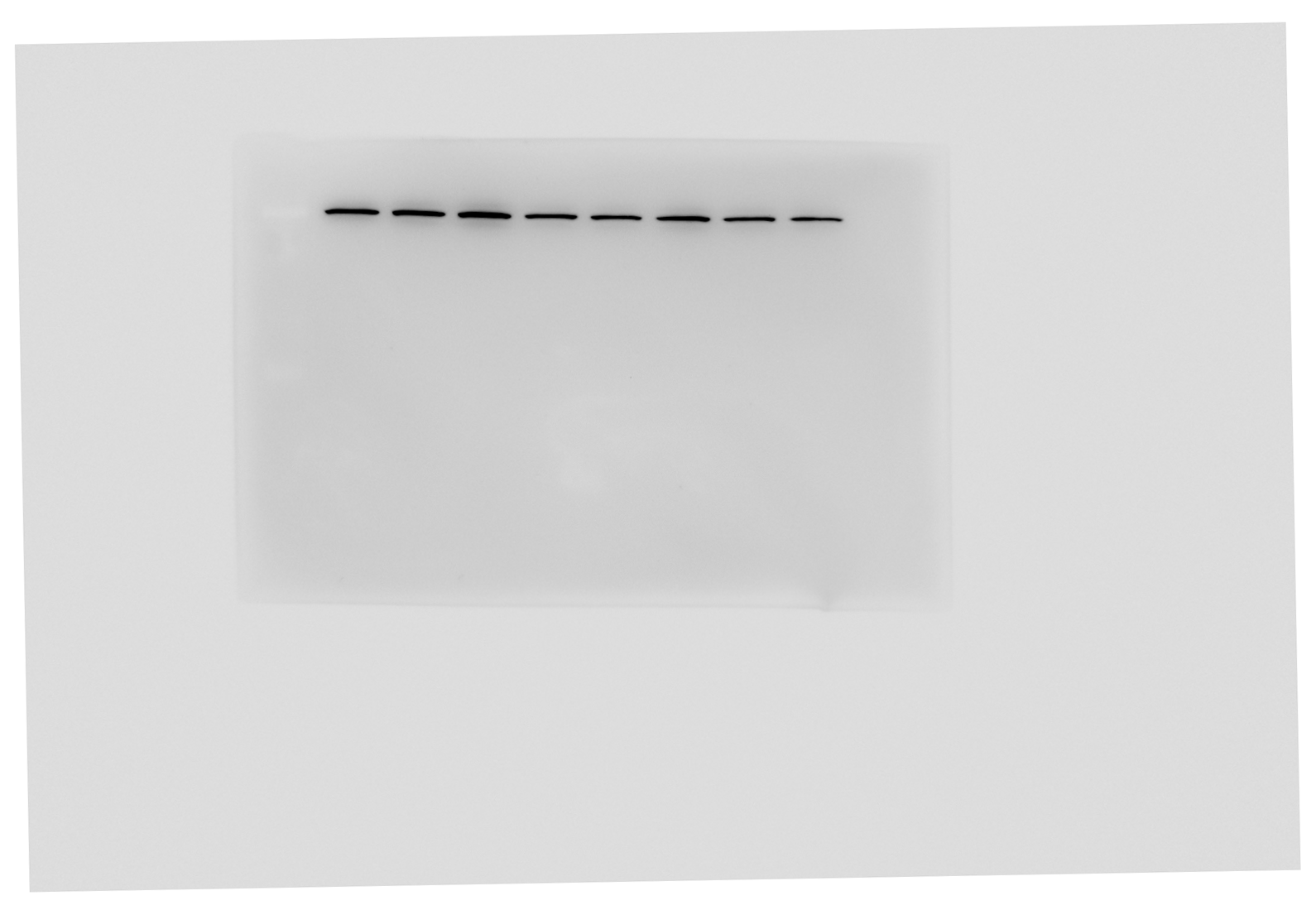

Supplement: Figure 2—figure supplement 1—source data 2. [file elife-106814-fig2-figsupp1-data2.zip › Figure 2-figure suppplement 1-source data 2/Figure 2-figure supplement 1A_Nrf1.tif]

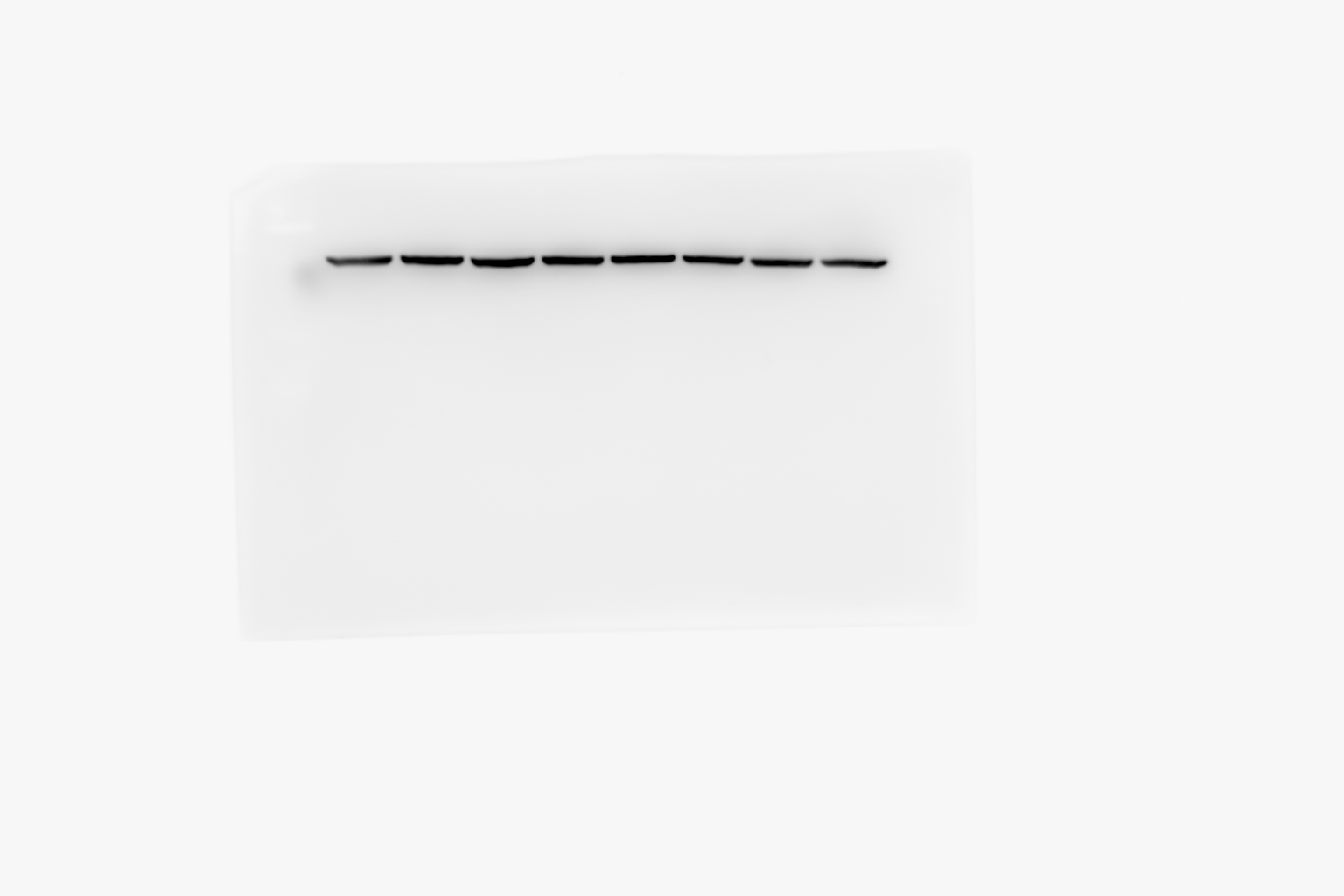

Supplement: Figure 3—source data 2. [file elife-106814-fig3-data2.zip › Figure 3-source data 2/Figure 3D_b-tubulin (for Gpx4).tif]

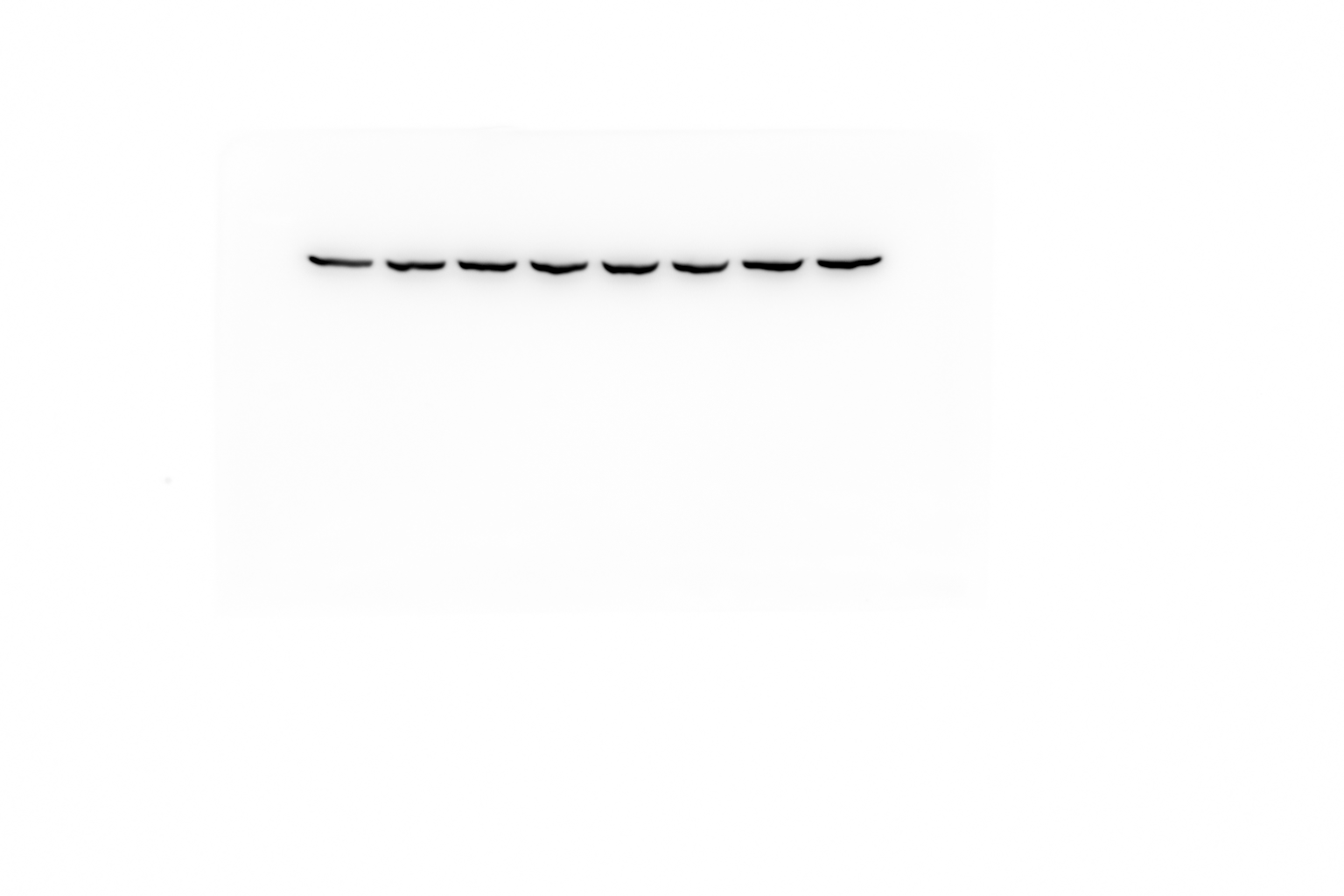

Supplement: Figure 3—source data 2. [file elife-106814-fig3-data2.zip › Figure 3-source data 2/Figure 3K_b-tubulin (12 h).tif]

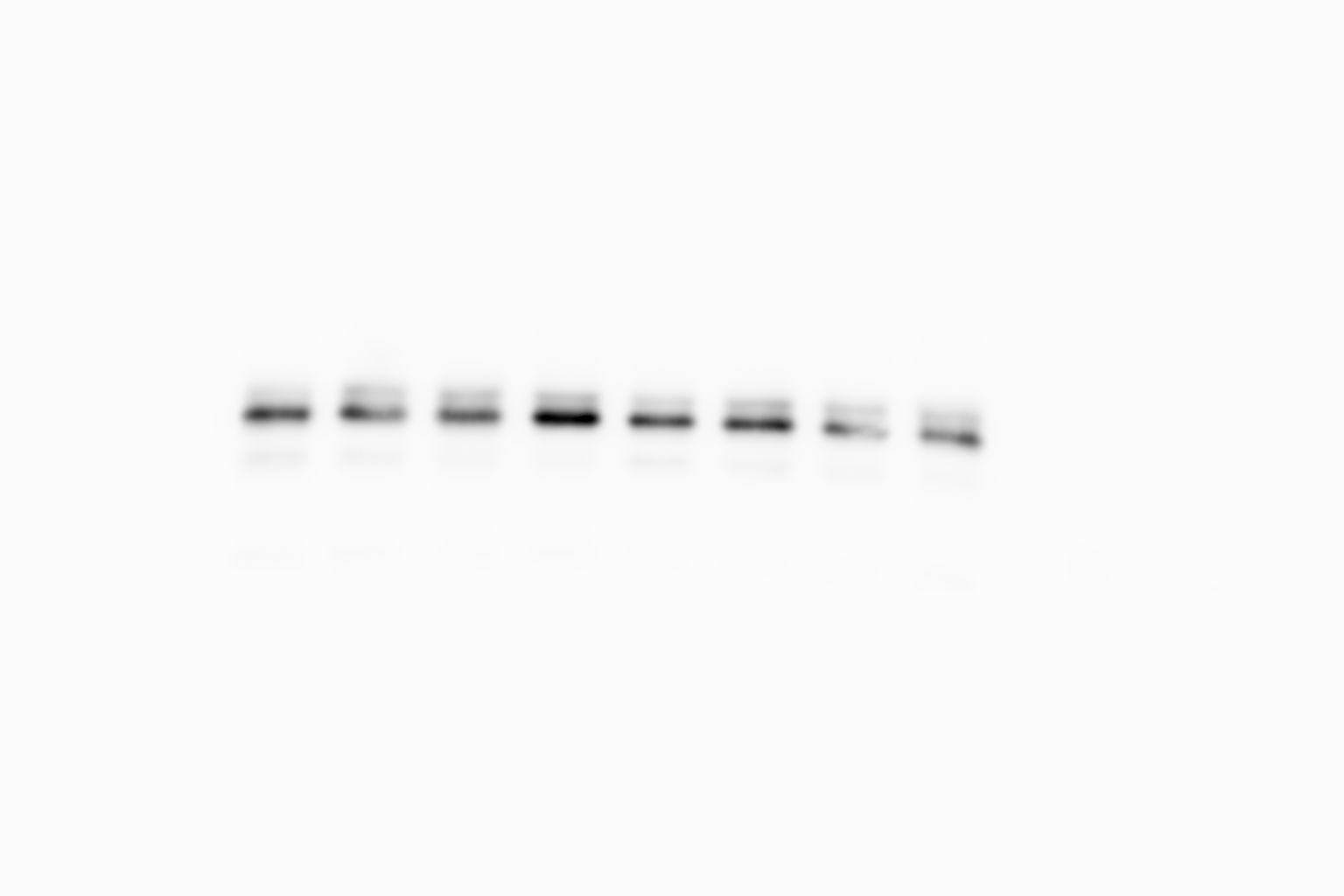

Supplement: Figure 3—source data 2. [file elife-106814-fig3-data2.zip › Figure 3-source data 2/Figure 3C_Fth.tif]

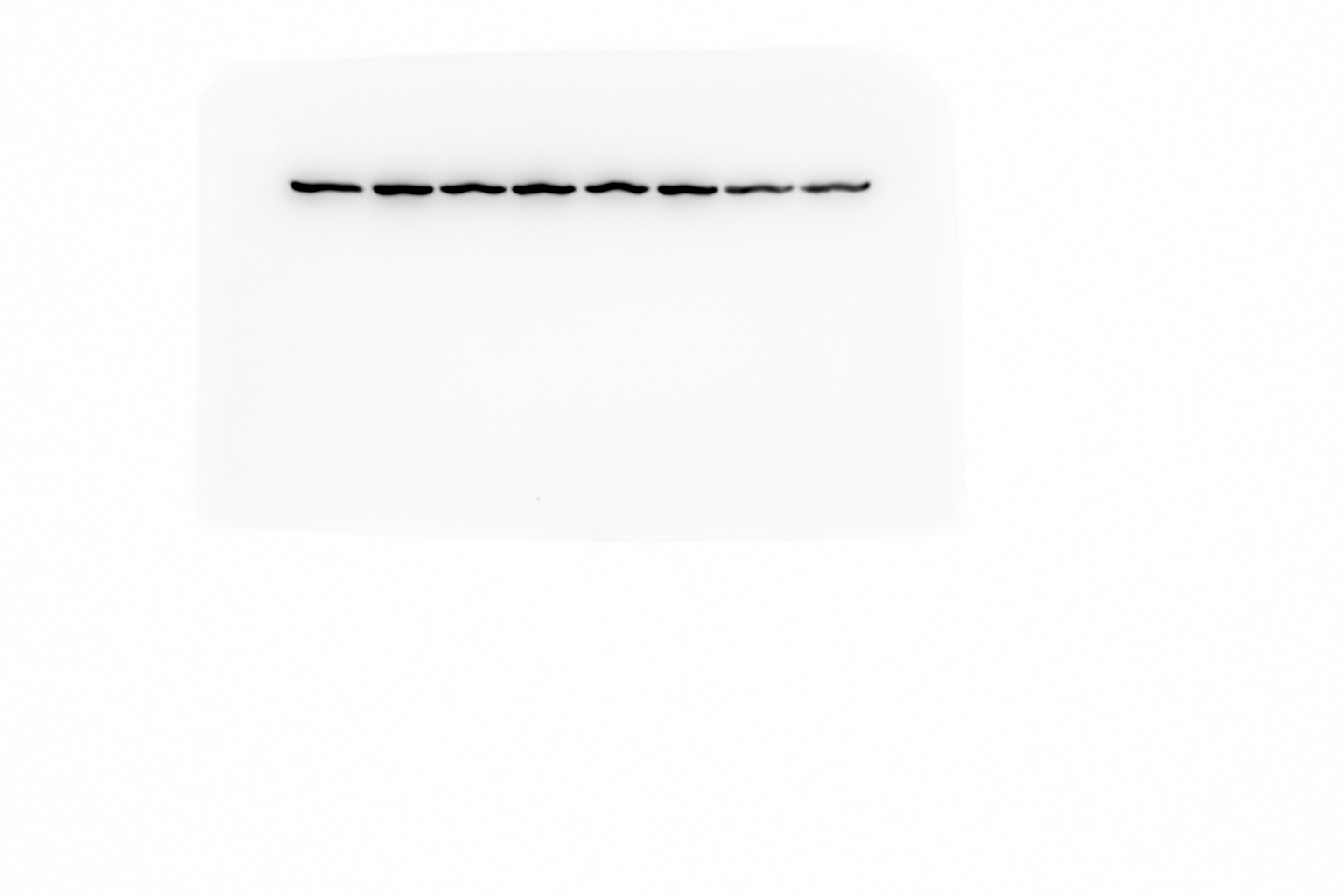

Supplement: Figure 3—source data 2. [file elife-106814-fig3-data2.zip › Figure 3-source data 2/Figure 3K_b-tubulin (36 h).tif]

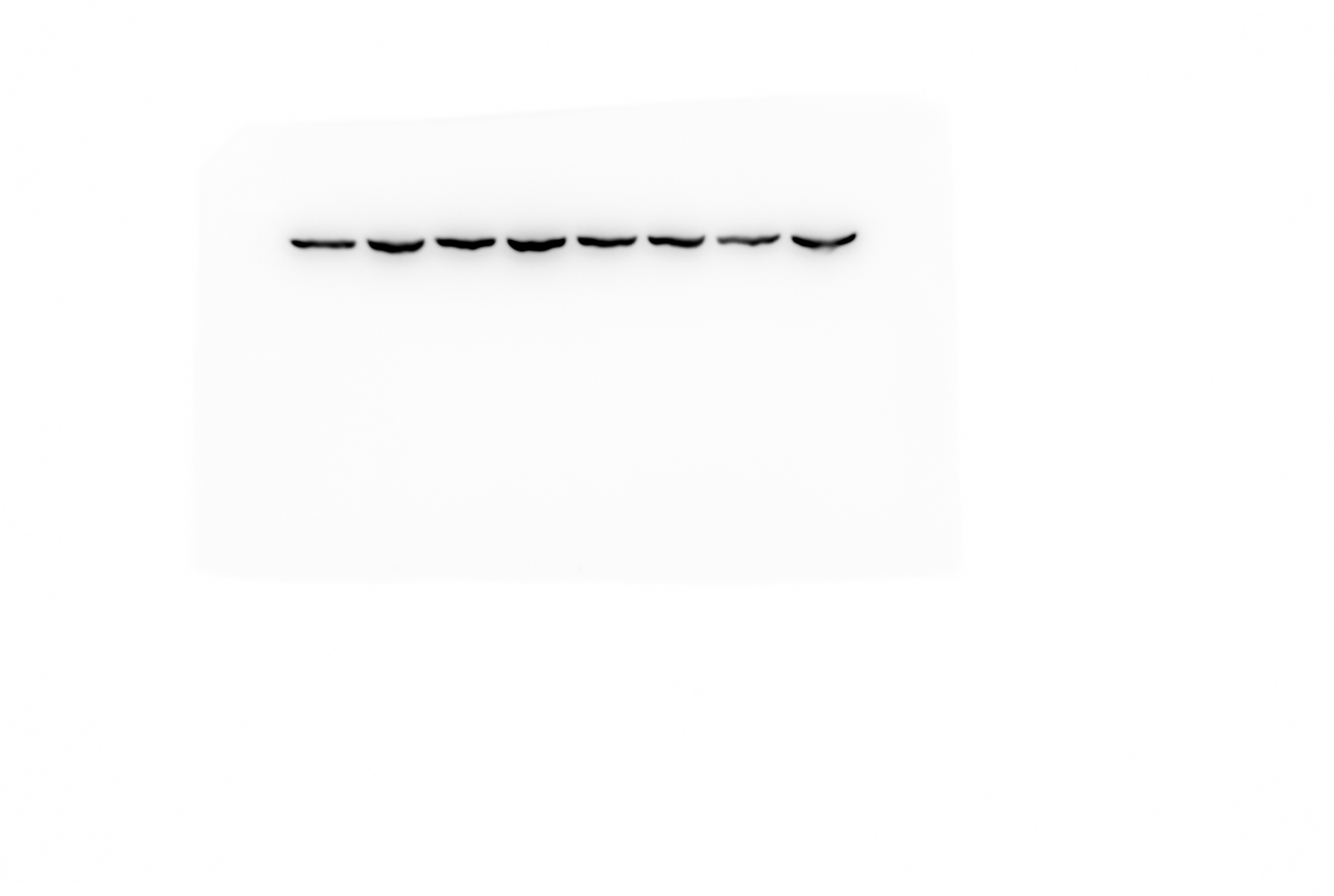

Supplement: Figure 3—source data 2. [file elife-106814-fig3-data2.zip › Figure 3-source data 2/Figure 3K_b-tubulin (24 h).tif]

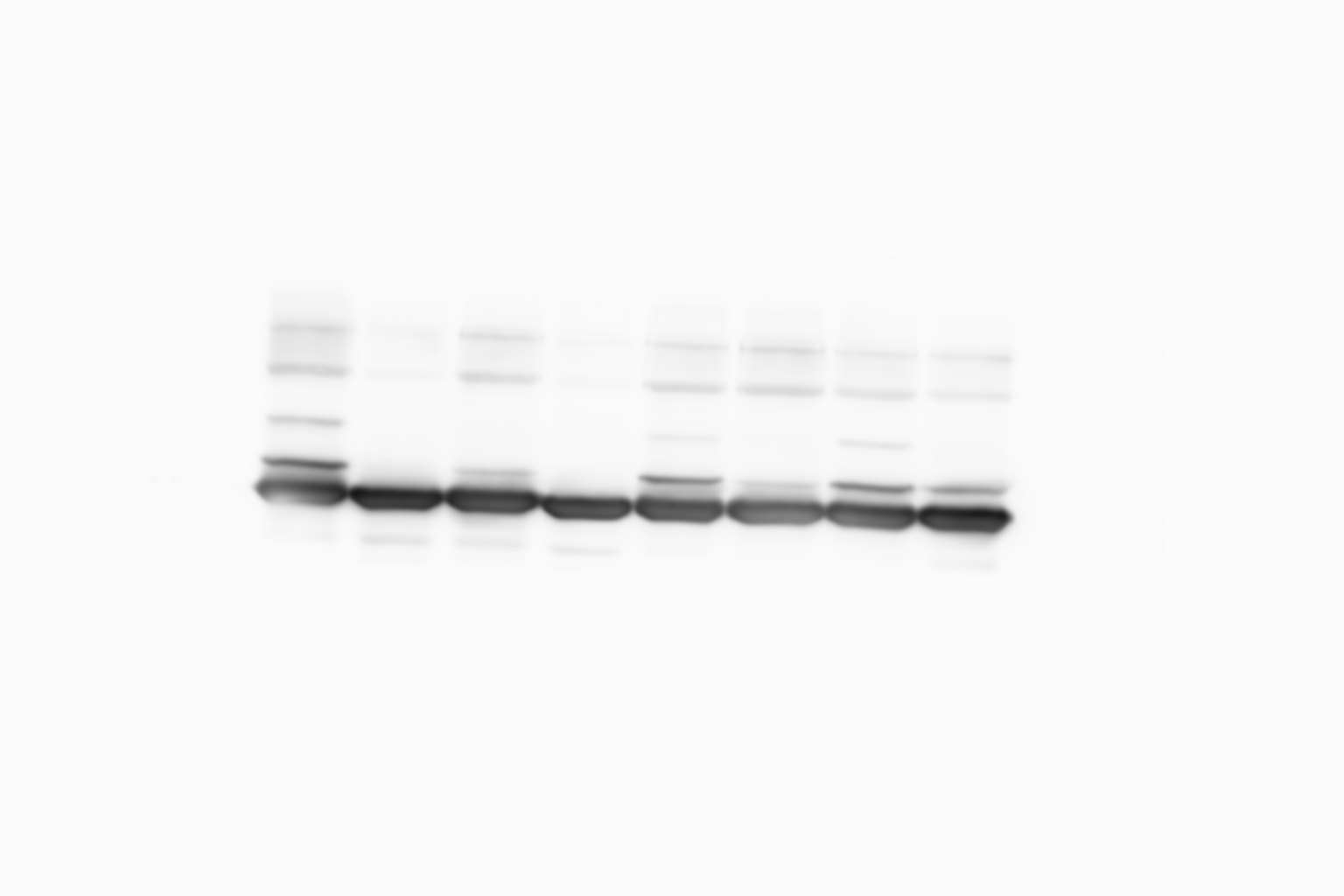

Supplement: Figure 3—source data 2. [file elife-106814-fig3-data2.zip › Figure 3-source data 2/Figure 3C_b-actin (for Fth).tif]

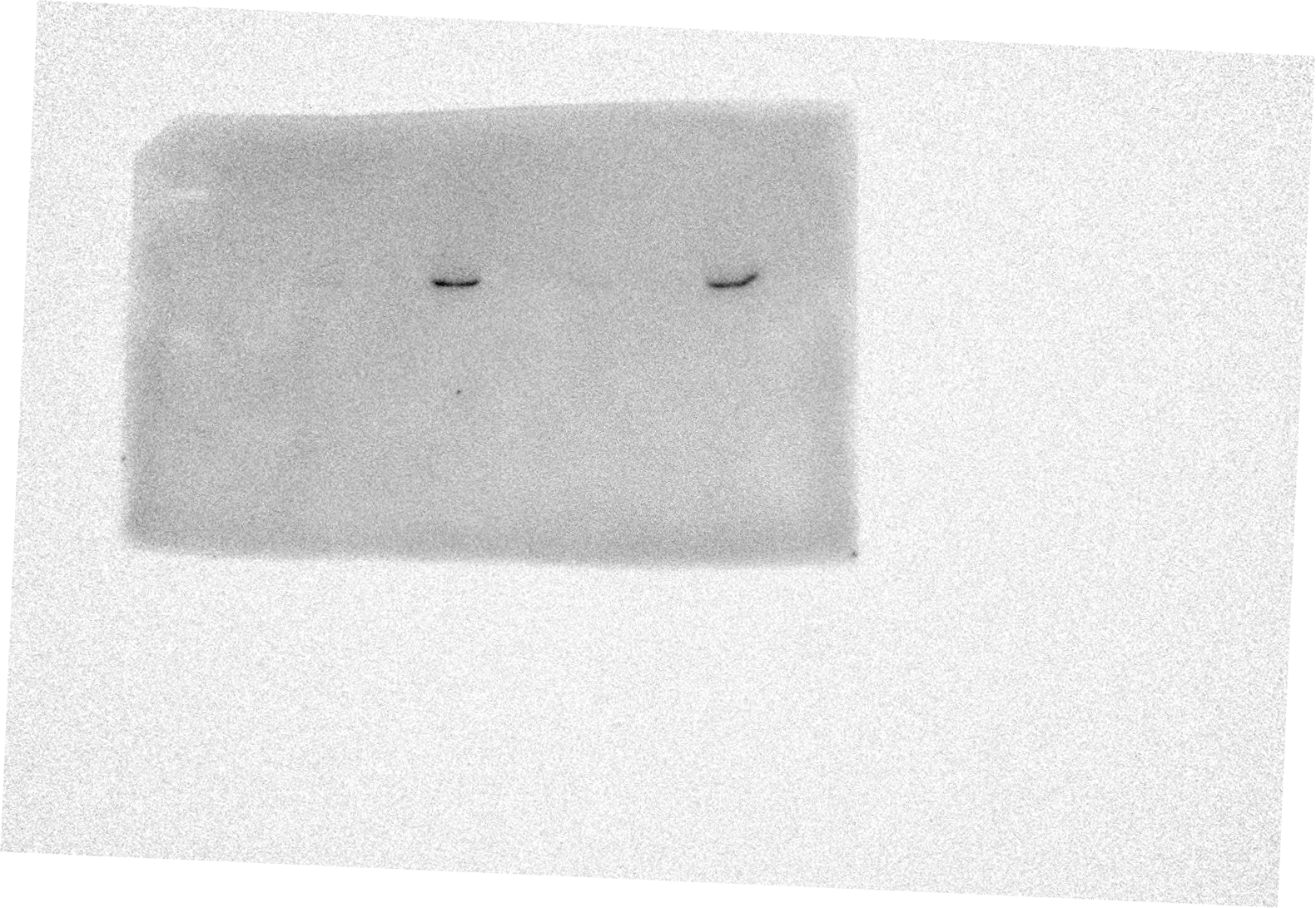

Supplement: Figure 3—source data 2. [file elife-106814-fig3-data2.zip › Figure 3-source data 2/Figure 3K_pcJun (24 h).tif]

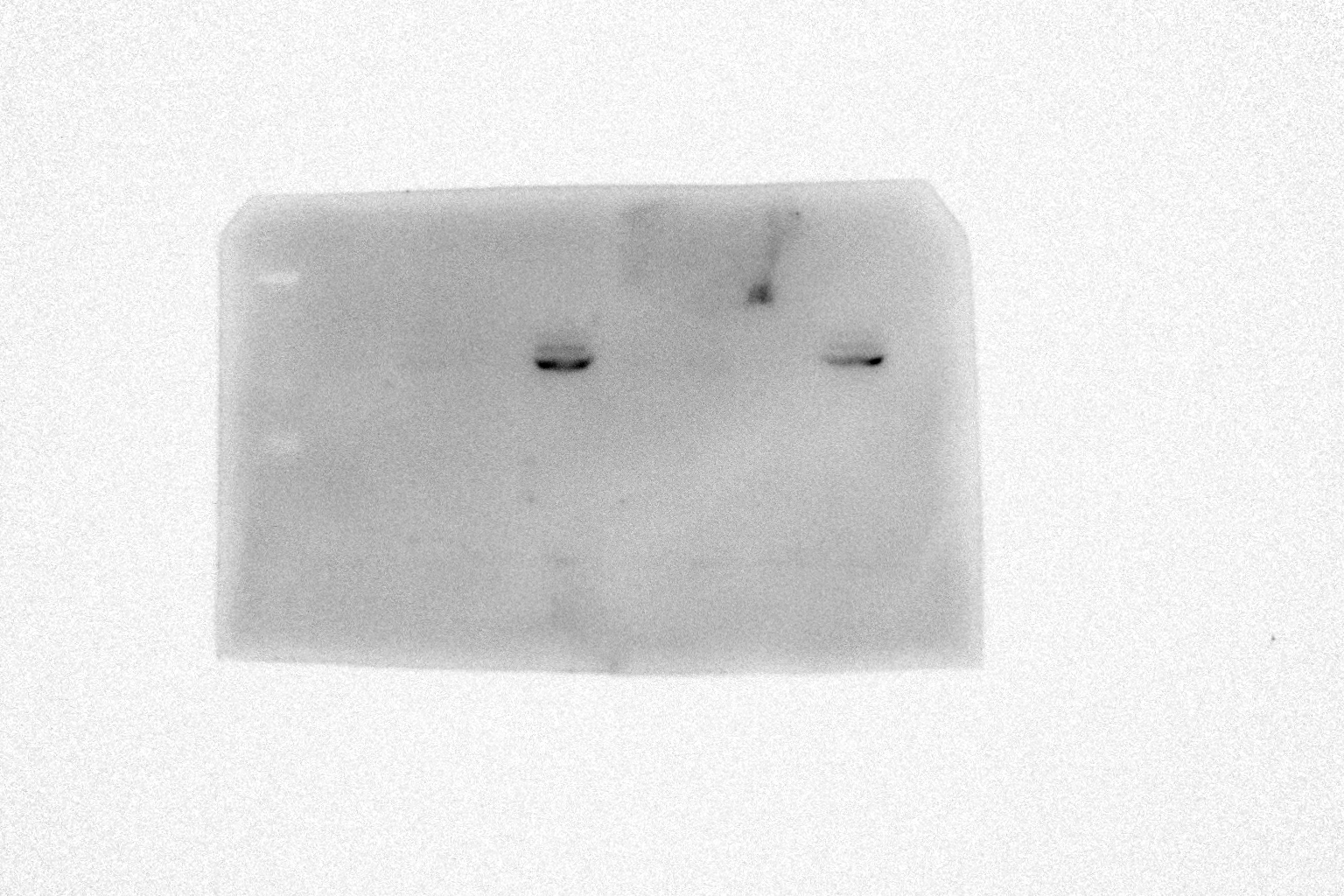

Supplement: Figure 3—source data 2. [file elife-106814-fig3-data2.zip › Figure 3-source data 2/Figure 3k_pcJun (36 h).tif]

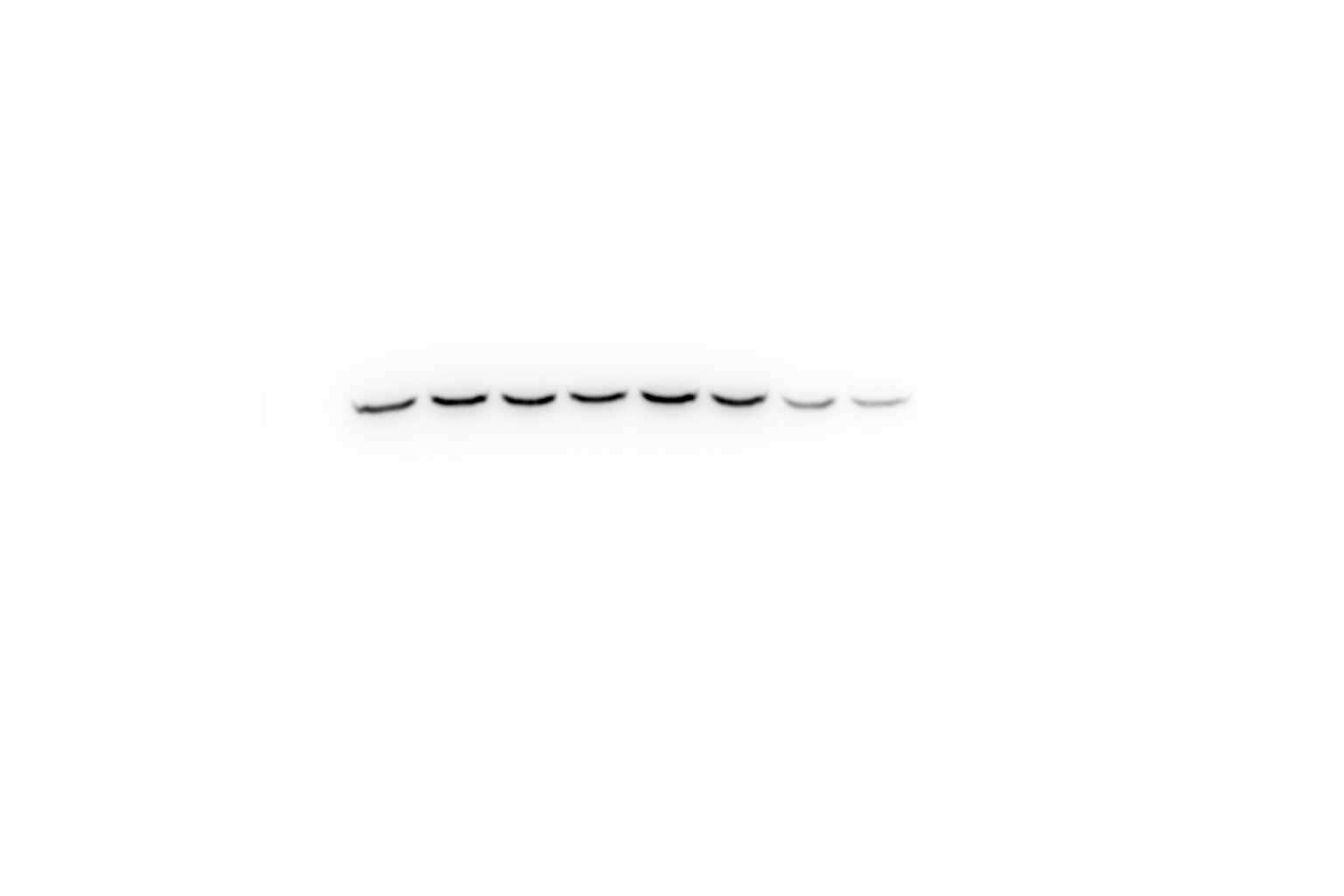

Supplement: Figure 3—source data 2. [file elife-106814-fig3-data2.zip › Figure 3-source data 2/Figure 3C_Ftl.tif]

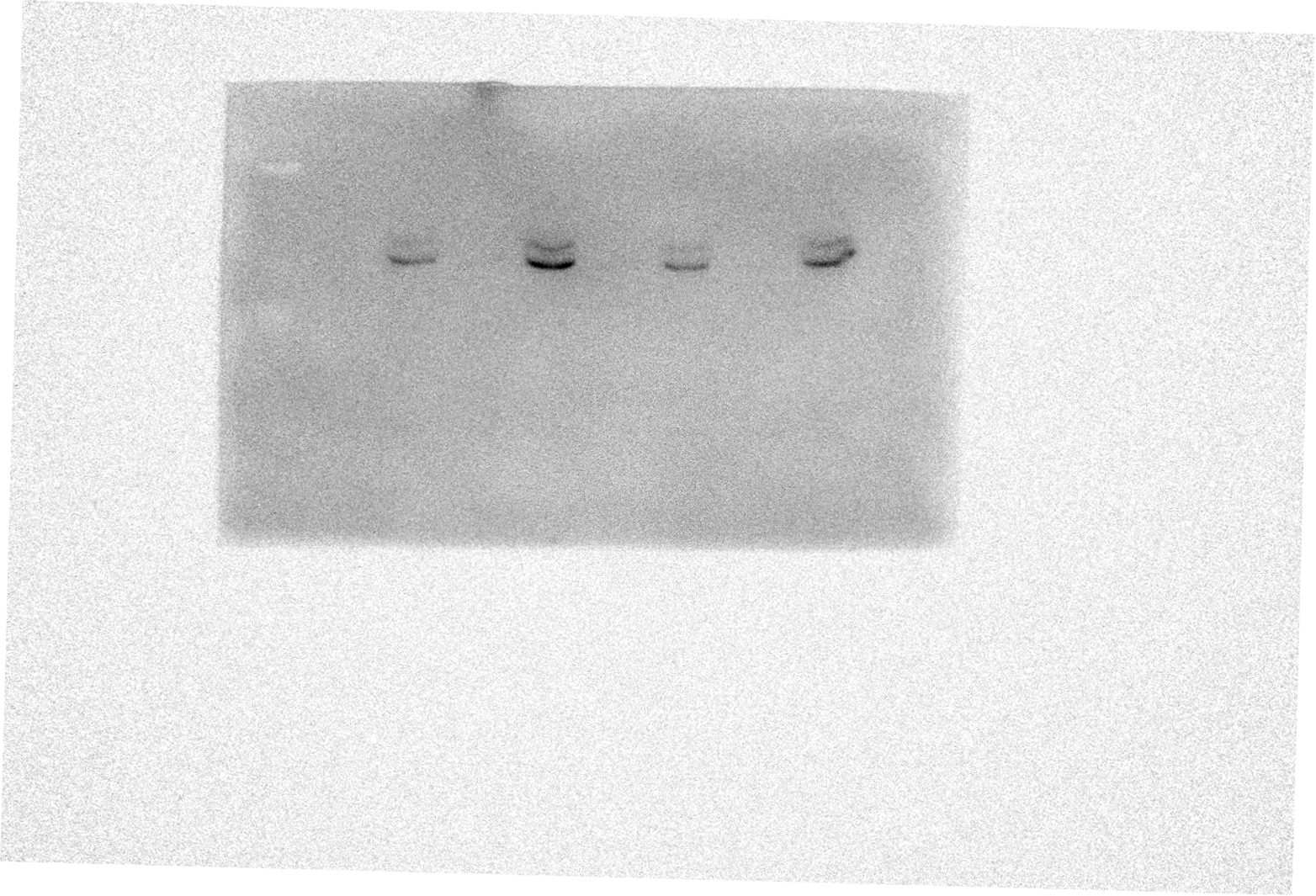

Supplement: Figure 3—source data 2. [file elife-106814-fig3-data2.zip › Figure 3-source data 2/Figure 3K-pcJun (12 h).tif]

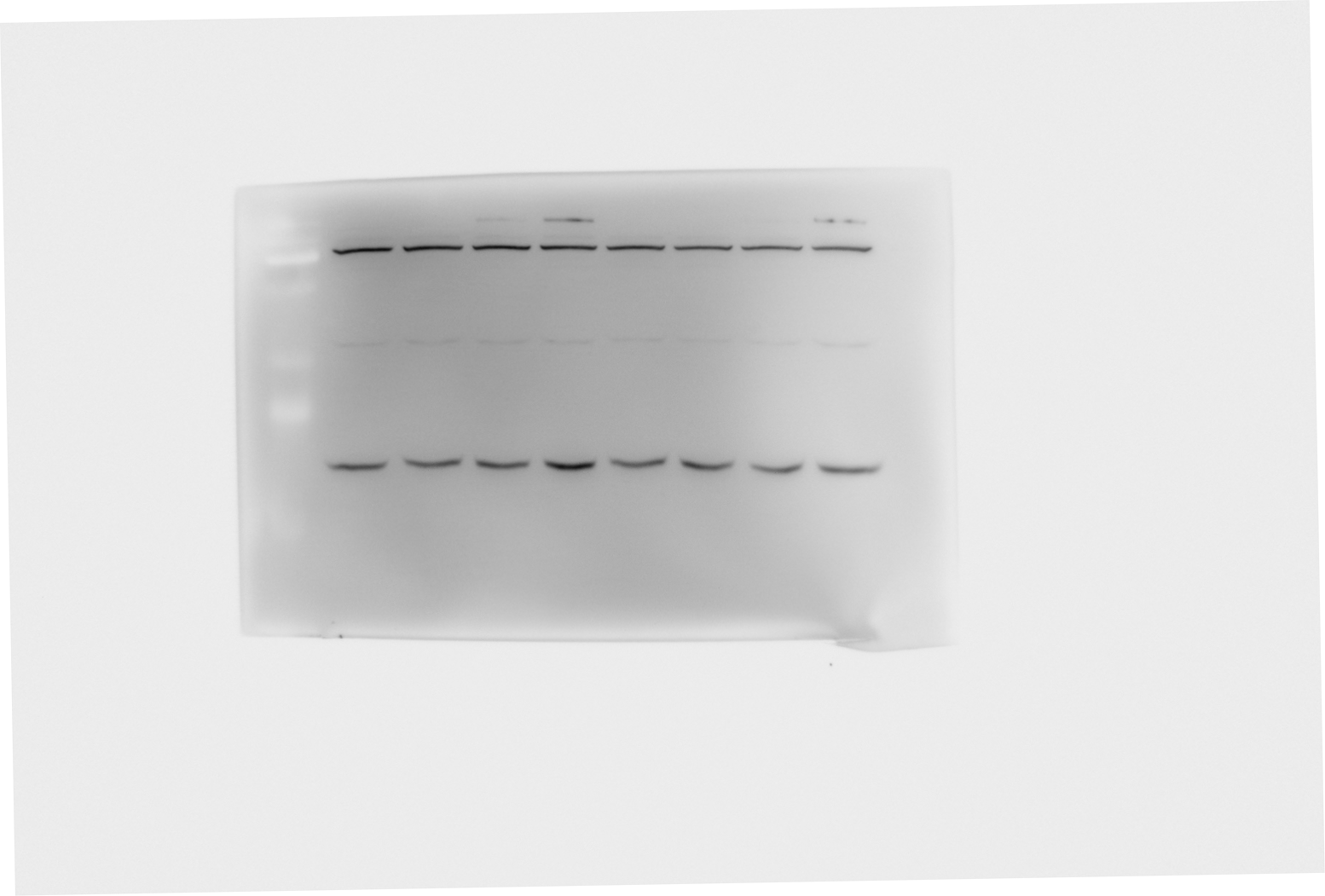

Supplement: Figure 3—source data 2. [file elife-106814-fig3-data2.zip › Figure 3-source data 2/Figure 3D_Gpx1.tif]

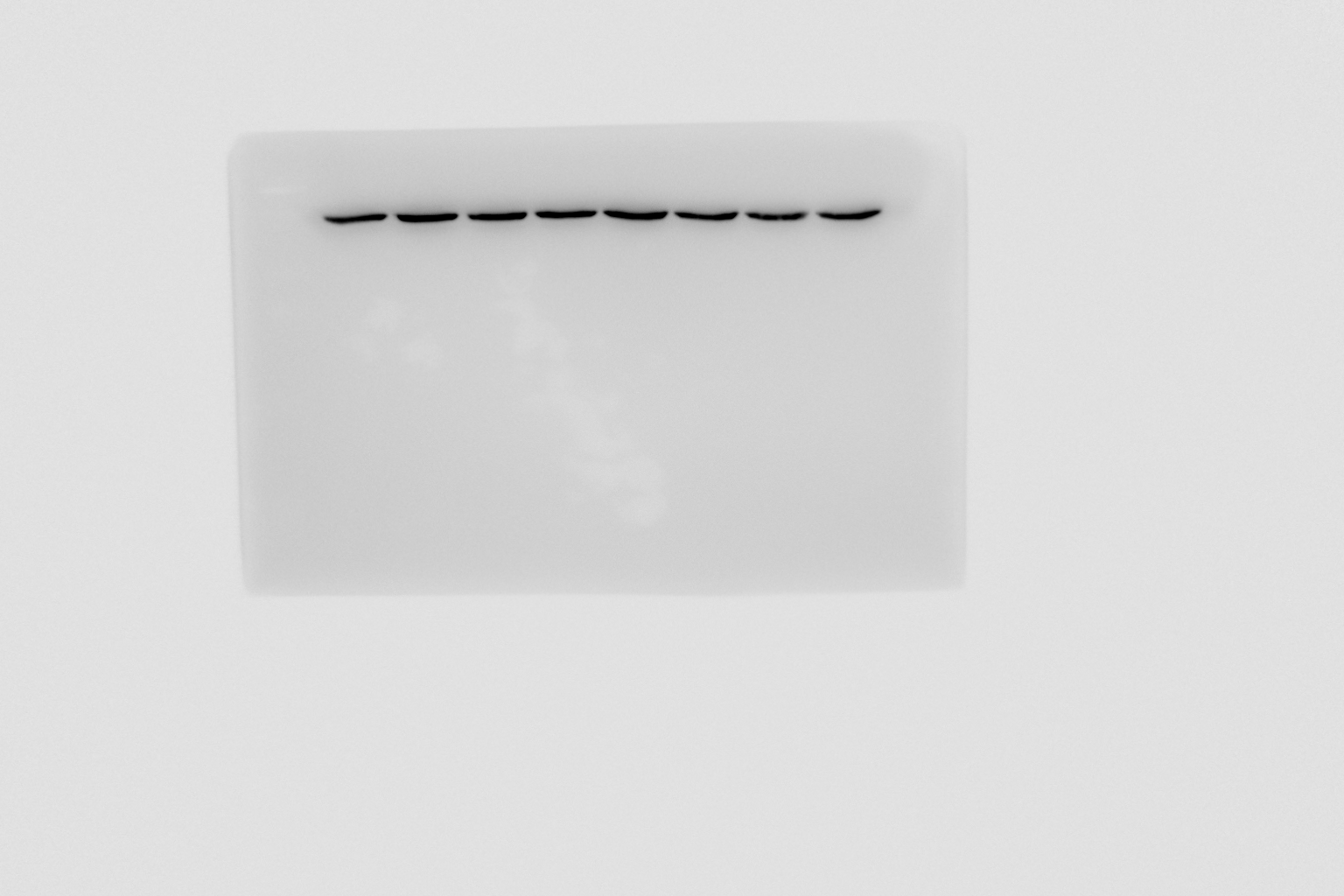

Supplement: Figure 3—source data 2. [file elife-106814-fig3-data2.zip › Figure 3-source data 2/Figure 3C_b-tubulin (for Ftl).tif]

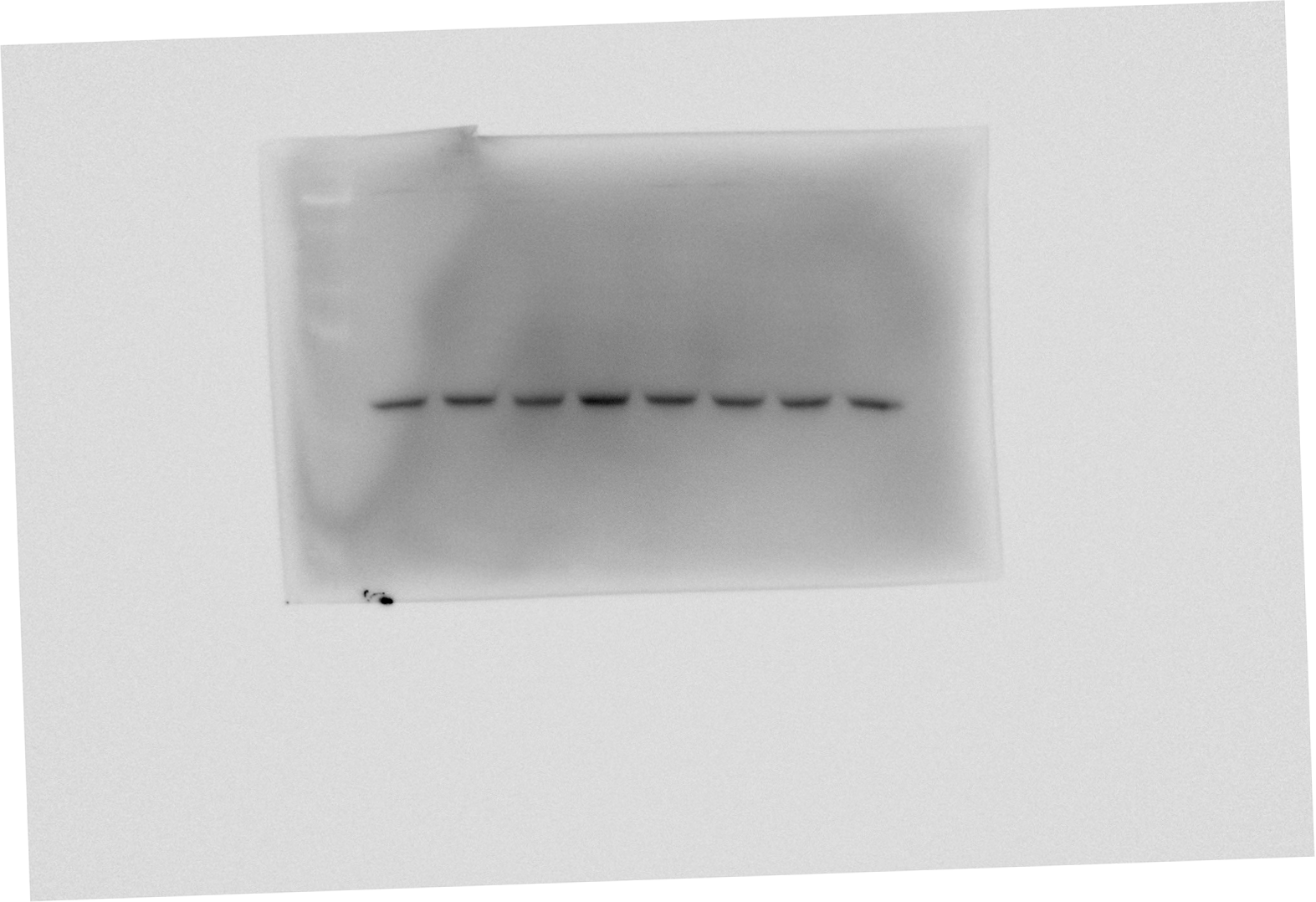

Supplement: Figure 3—source data 2. [file elife-106814-fig3-data2.zip › Figure 3-source data 2/Figure 3D_Gpx4.tif]

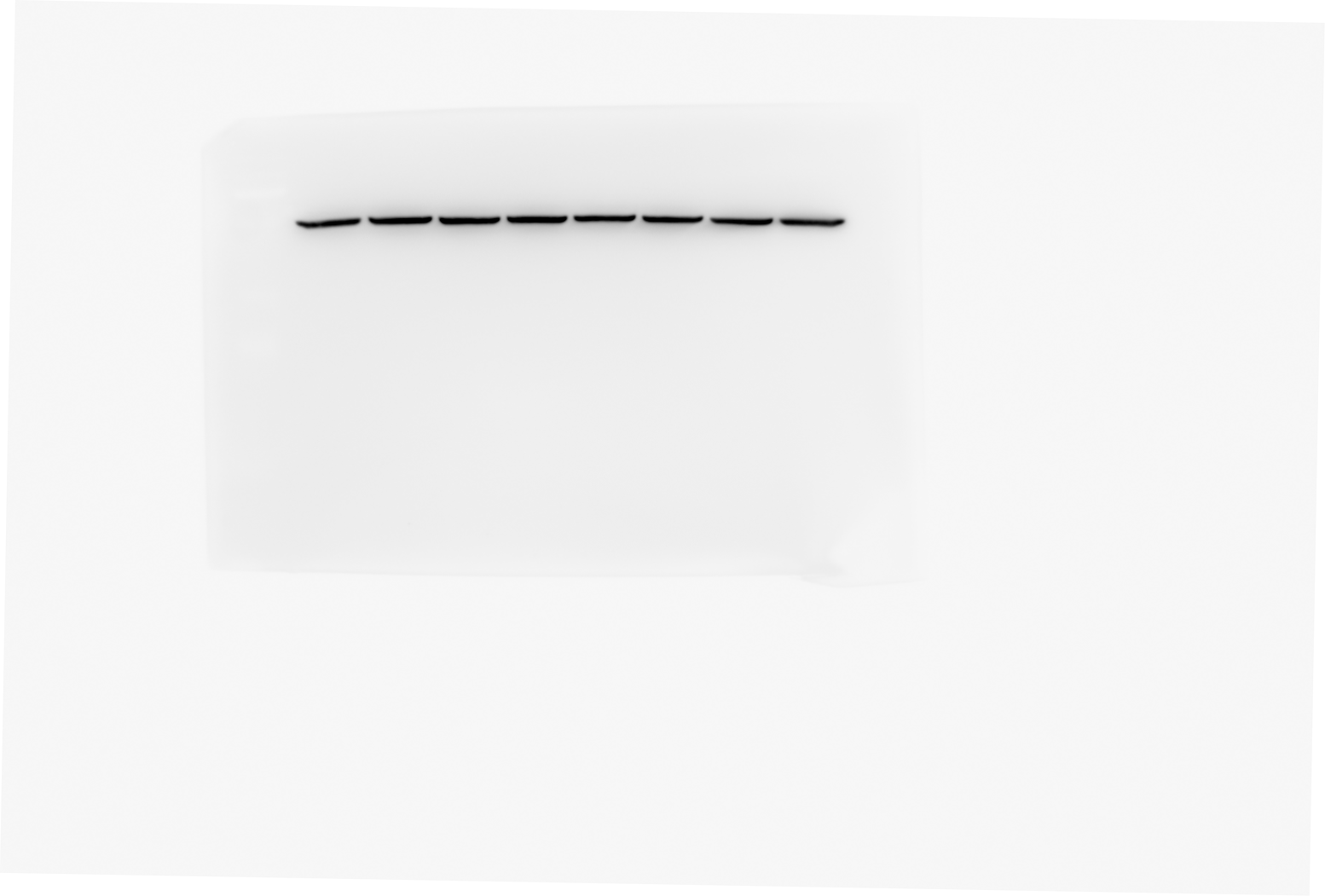

Supplement: Figure 3—source data 2. [file elife-106814-fig3-data2.zip › Figure 3-source data 2/Figure 3D_b-tubulin (for Gpx1).tif]

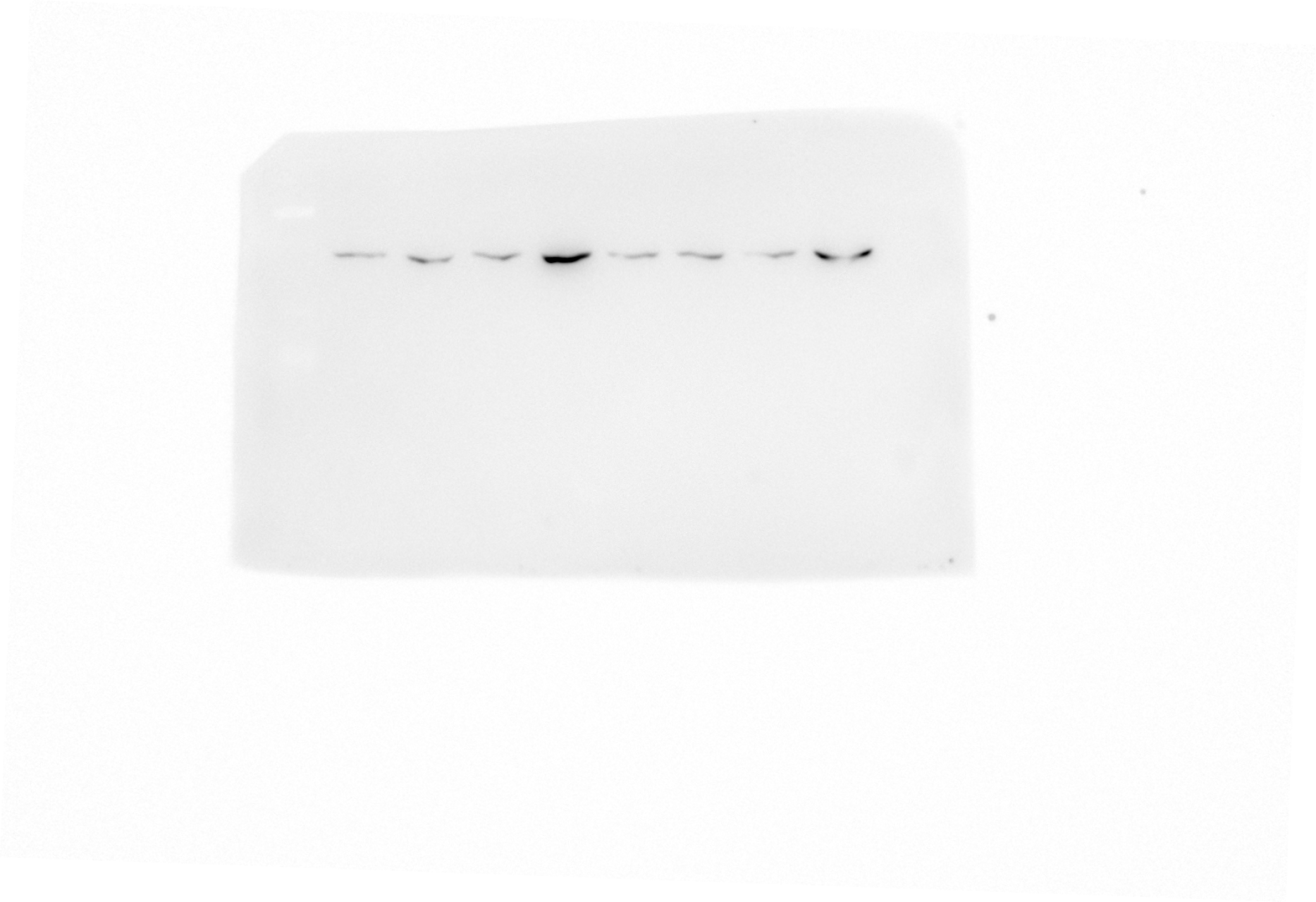

Supplement: Figure 3—source data 2. [file elife-106814-fig3-data2.zip › Figure 3-source data 2/Figure 3K_pASK1 (24 h).tif]

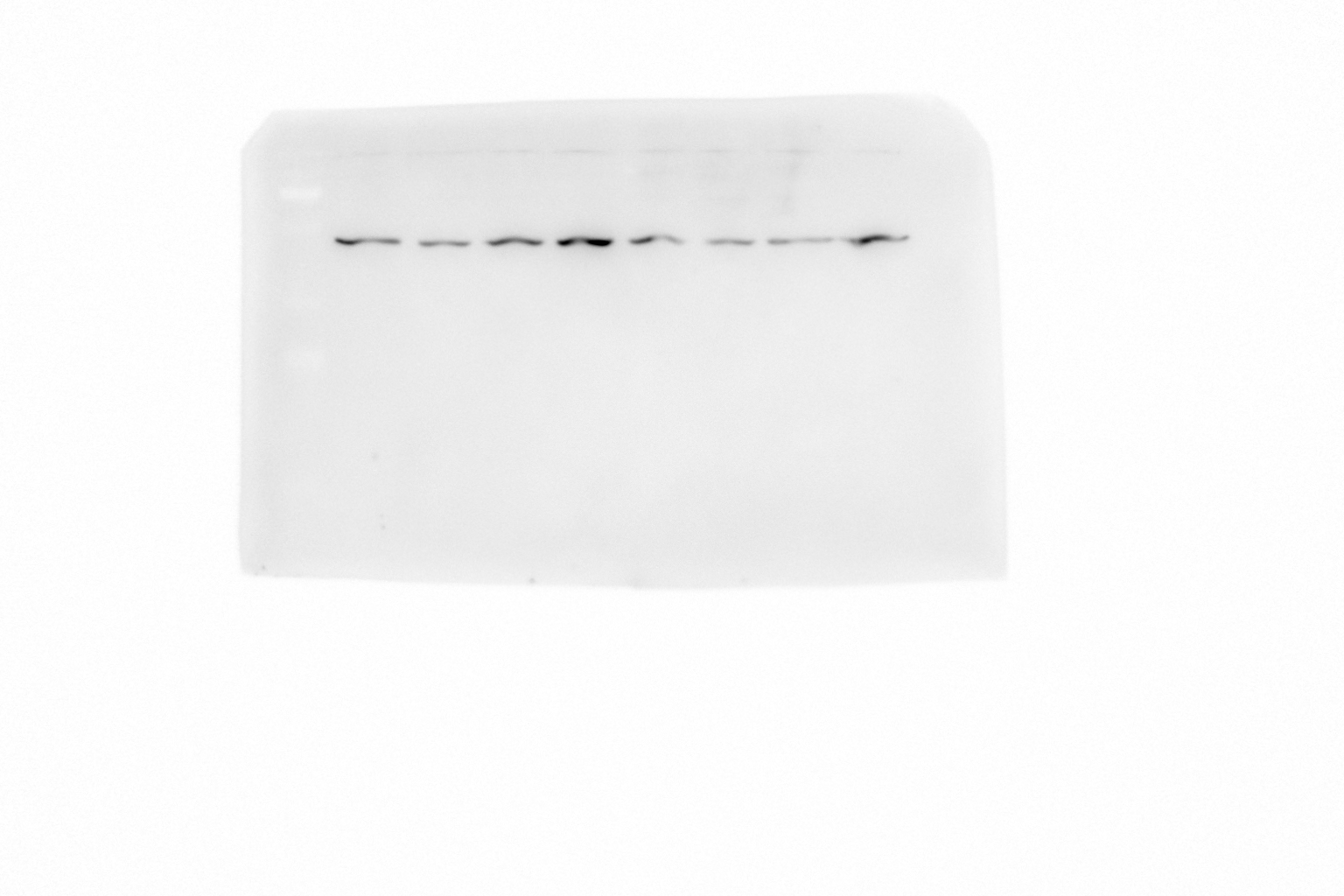

Supplement: Figure 3—source data 2. [file elife-106814-fig3-data2.zip › Figure 3-source data 2/Figure 3K_pASK1 (36 h).tif]

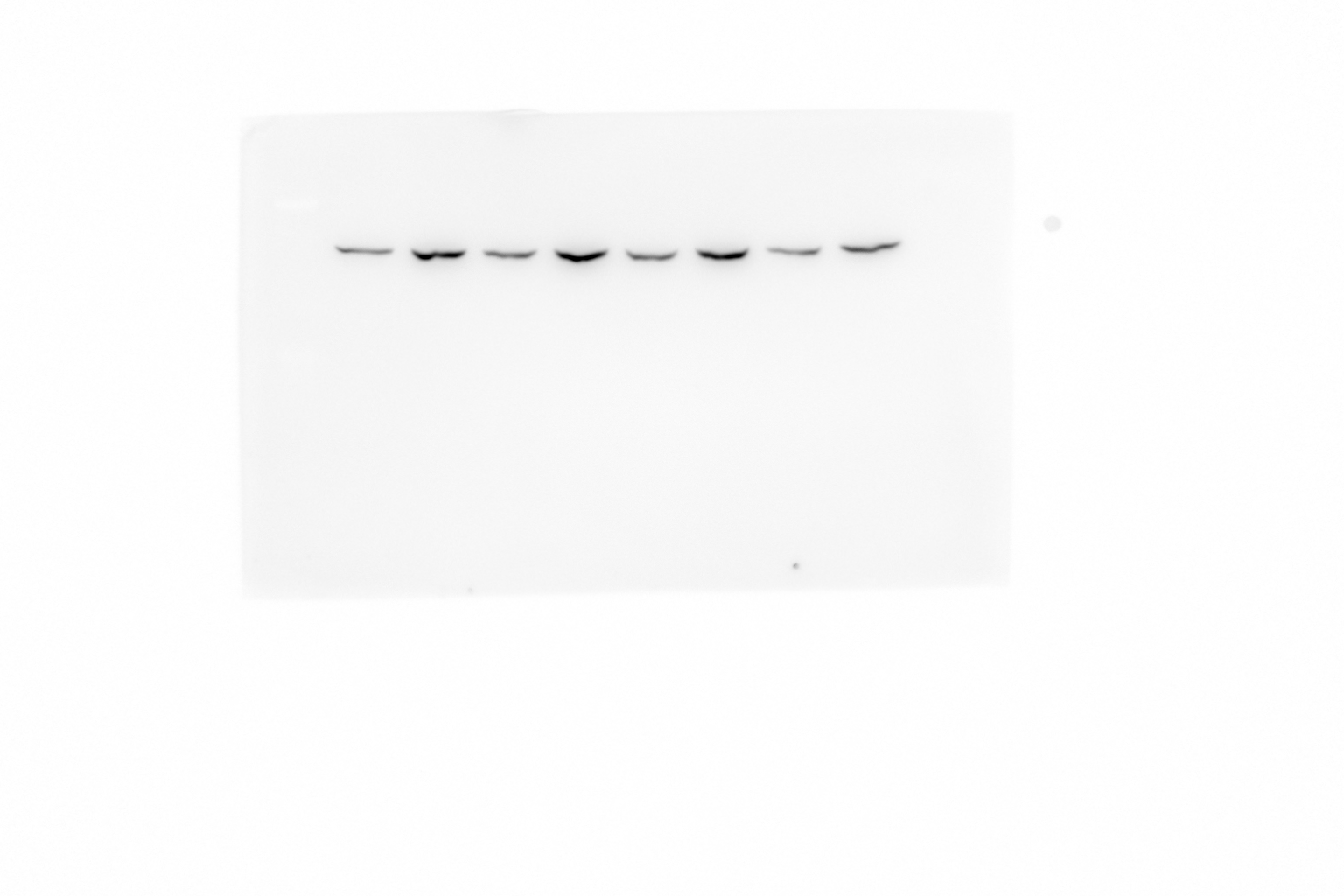

Supplement: Figure 3—source data 2. [file elife-106814-fig3-data2.zip › Figure 3-source data 2/Figure 3K_pASK1 (12 h).tif]

Figure 4A: Nrf2

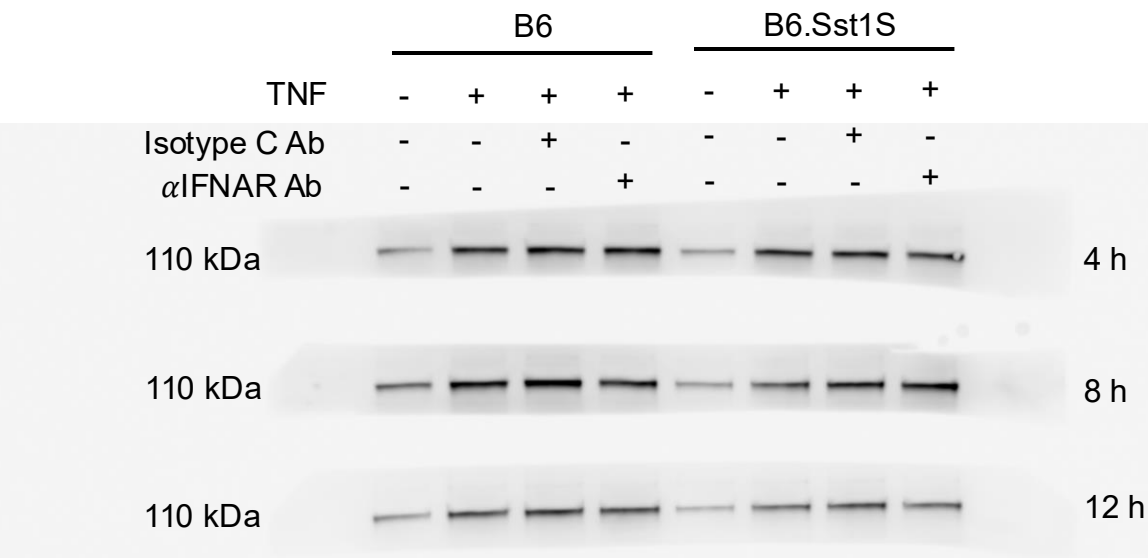

Figure 4A:  $\beta$ -tubulin

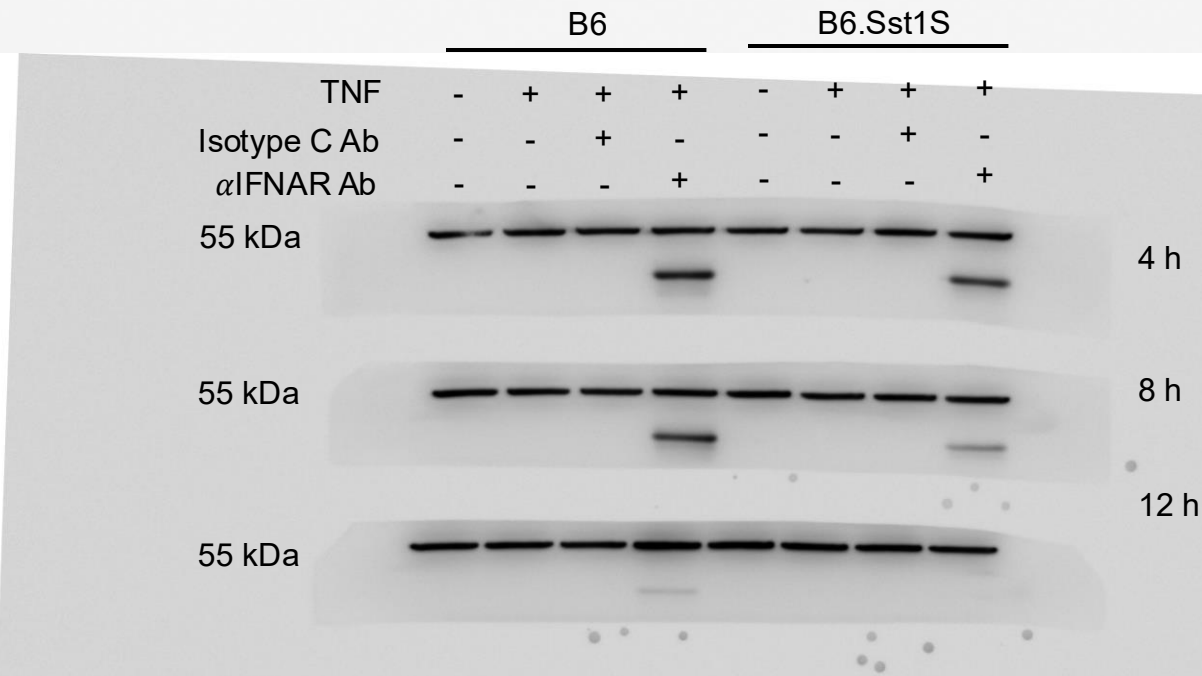

Figure 4B: Ftl

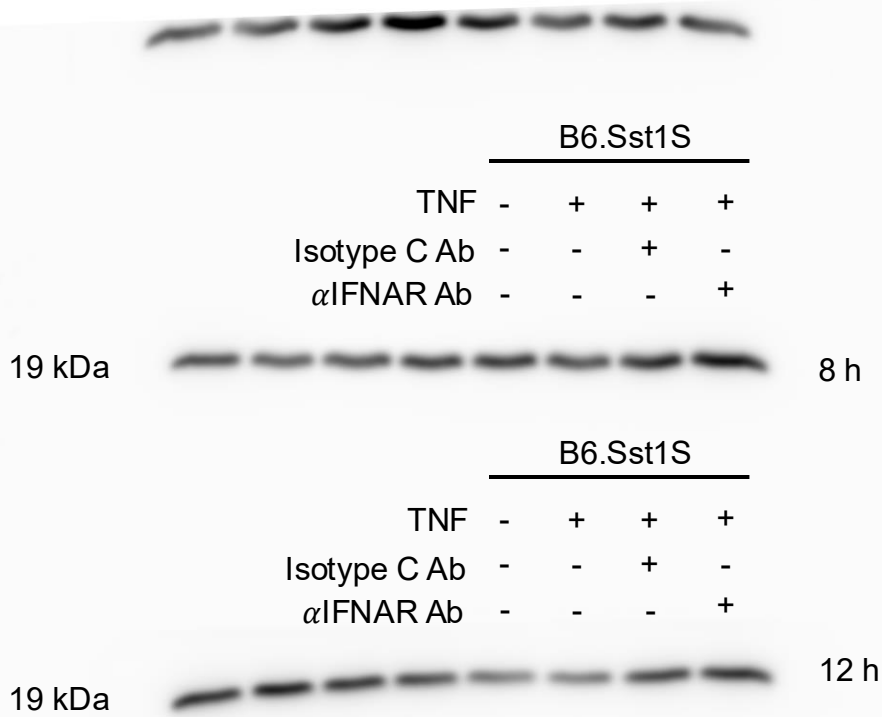

Figure 4B:  $\beta$ -tubulin

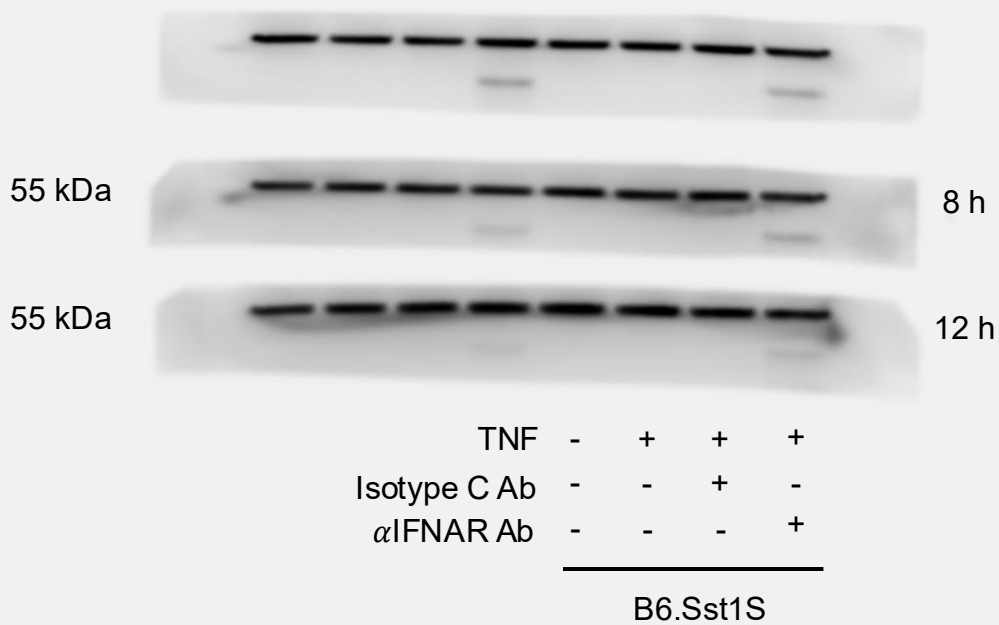

Supplement: Figure 4—source data 1. [file elife-106814-fig4-data1.zip › Figure 4-source data 1.pdf]

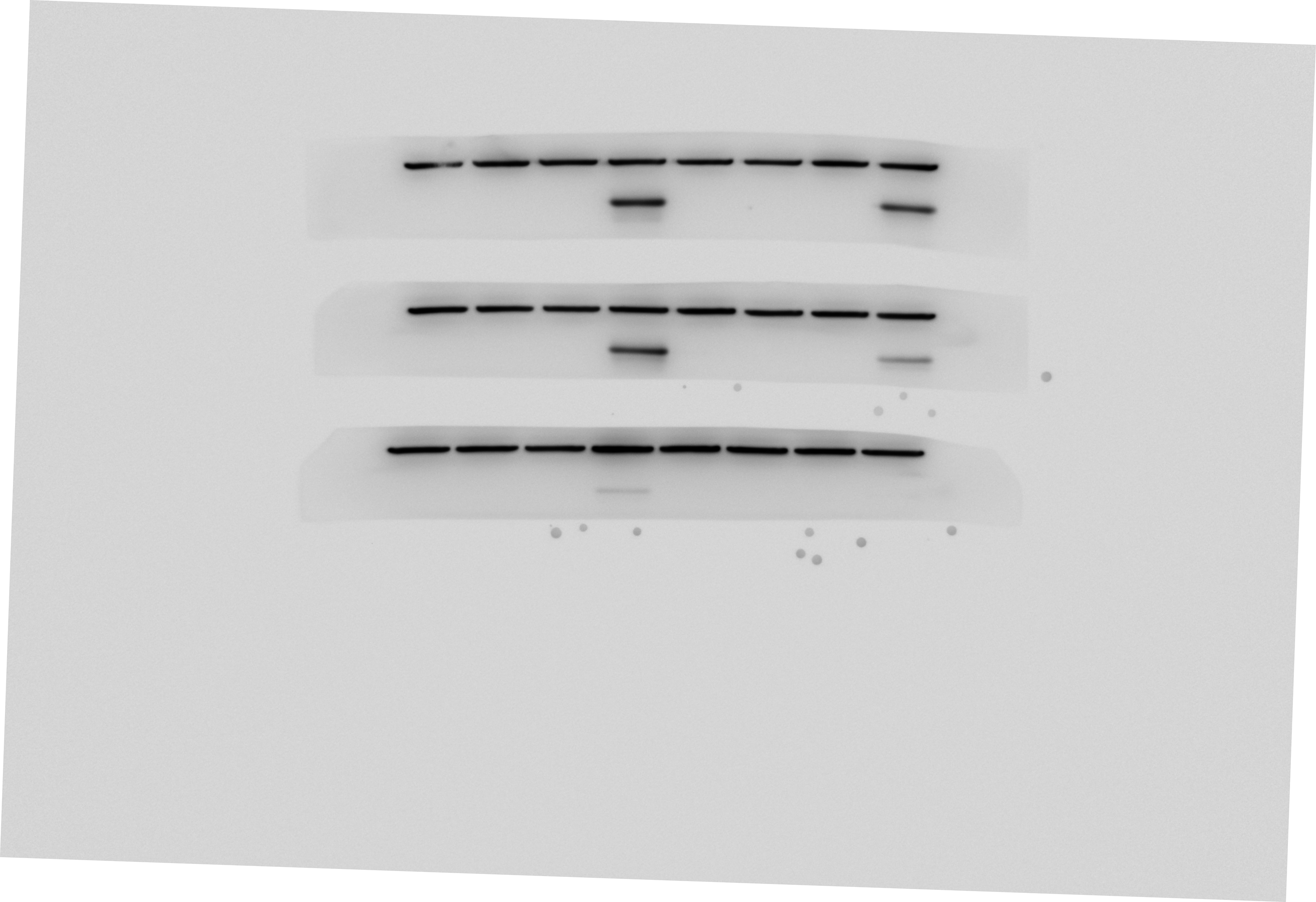

Supplement: Figure 4—source data 2. [file elife-106814-fig4-data2.zip › Figure 4-source data 2/Figure 4A_b-tubulin (for Nrf2).tif]

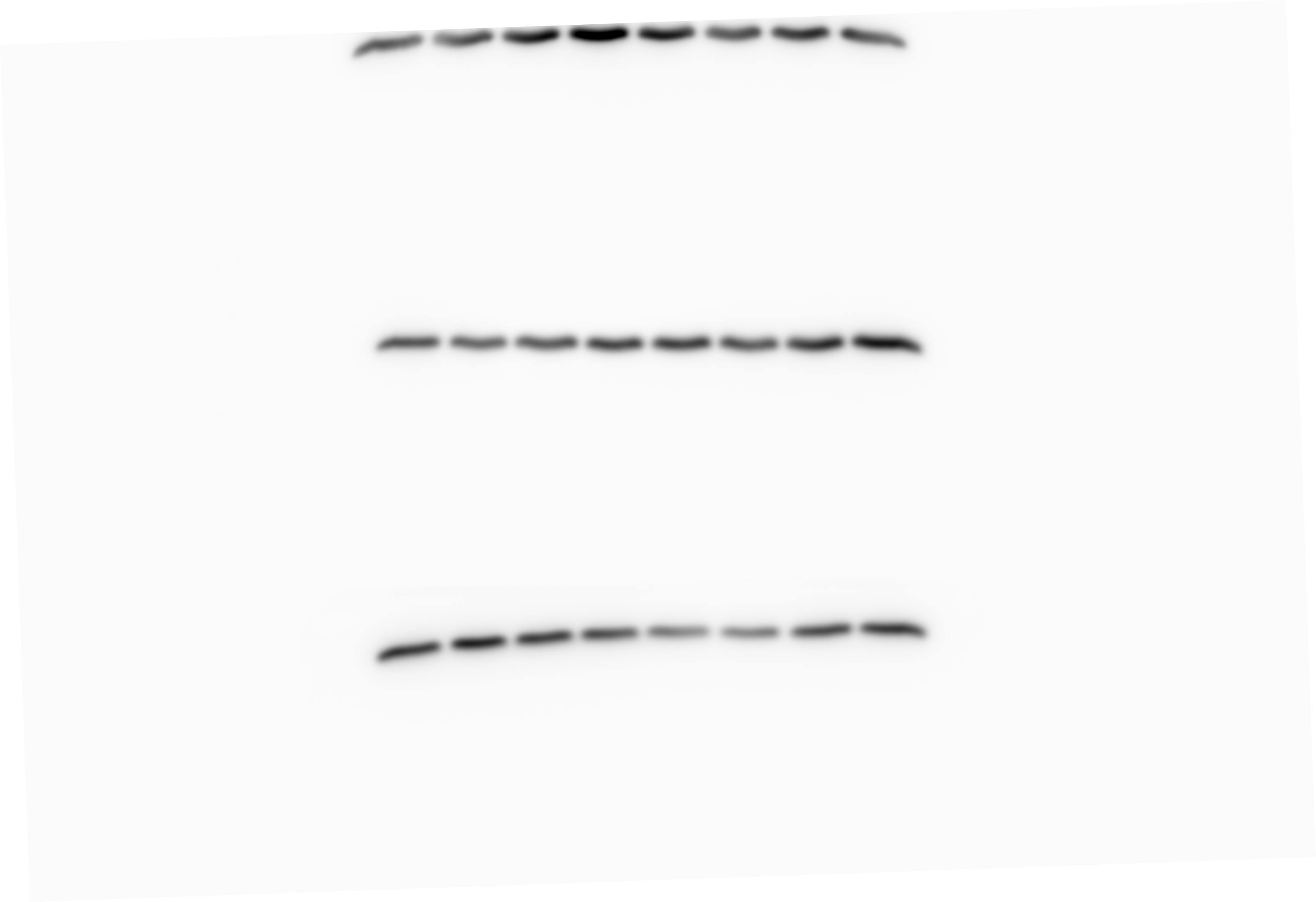

Supplement: Figure 4—source data 2. [file elife-106814-fig4-data2.zip › Figure 4-source data 2/Figure 4B_Ftl.tif]

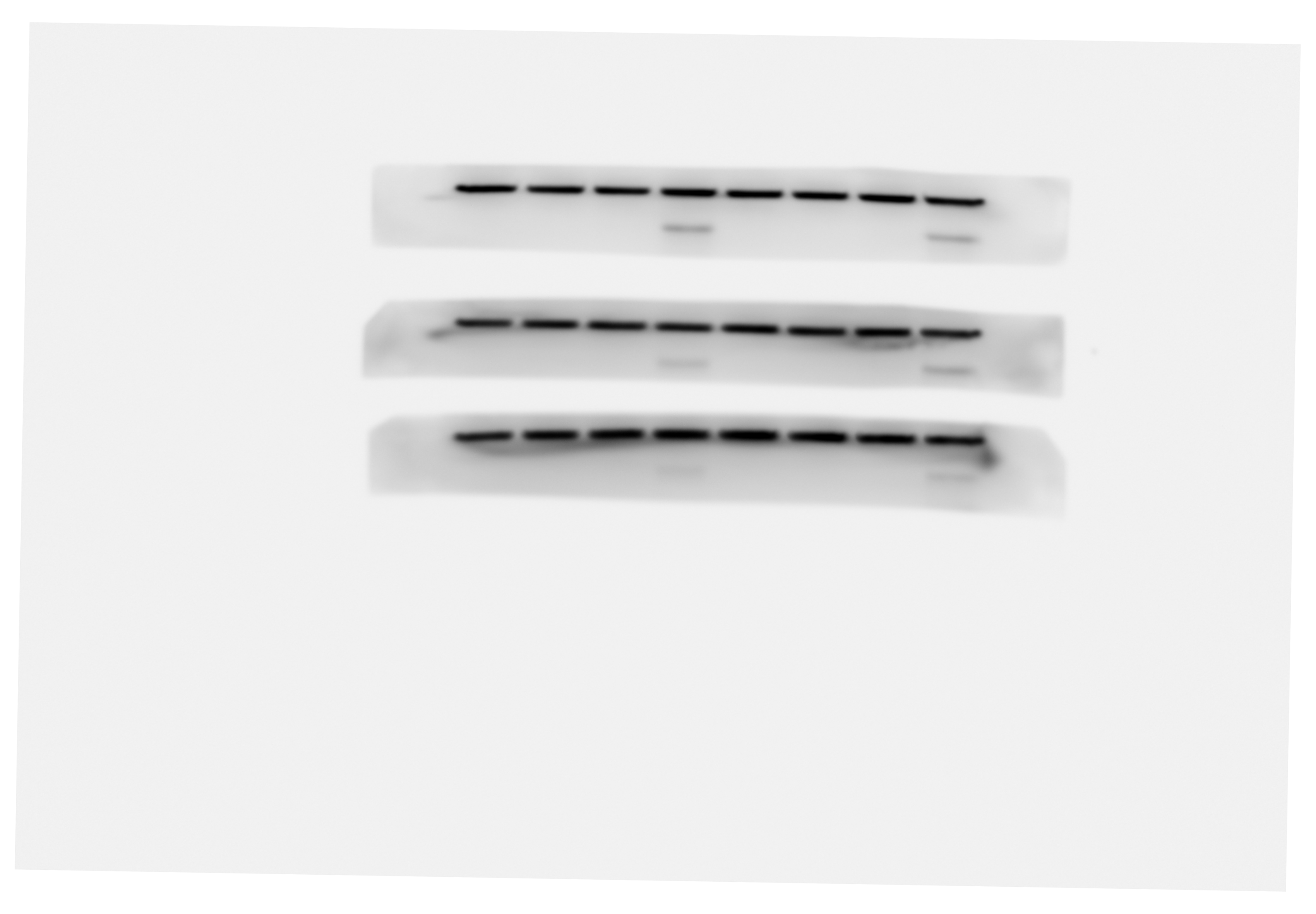

Supplement: Figure 4—source data 2. [file elife-106814-fig4-data2.zip › Figure 4-source data 2/Figure 4B_b-tubulin (for Ftl).tif]

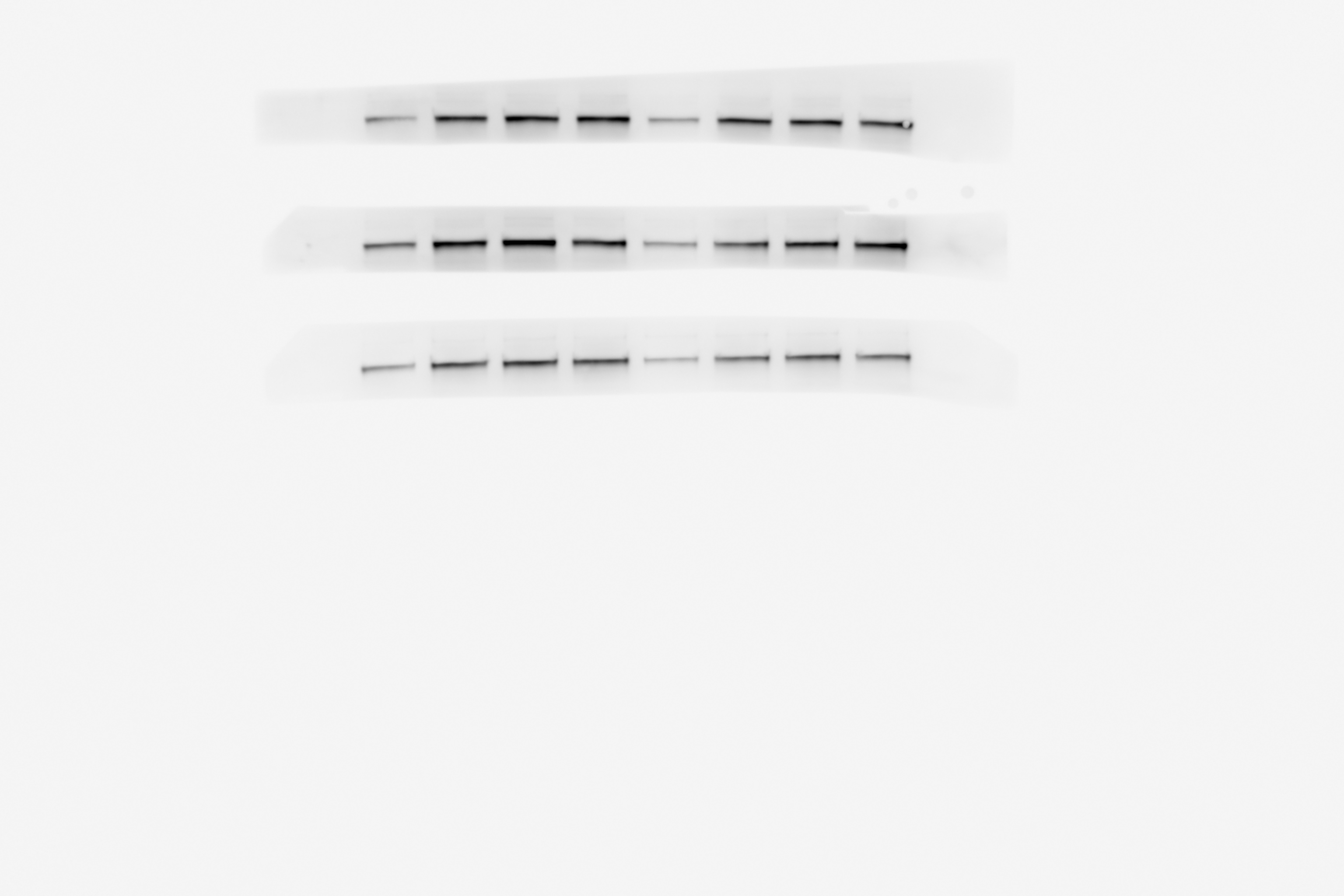

Supplement: Figure 4—source data 2. [file elife-106814-fig4-data2.zip › Figure 4-source data 2/Figure 4A_Nrf2.tif]

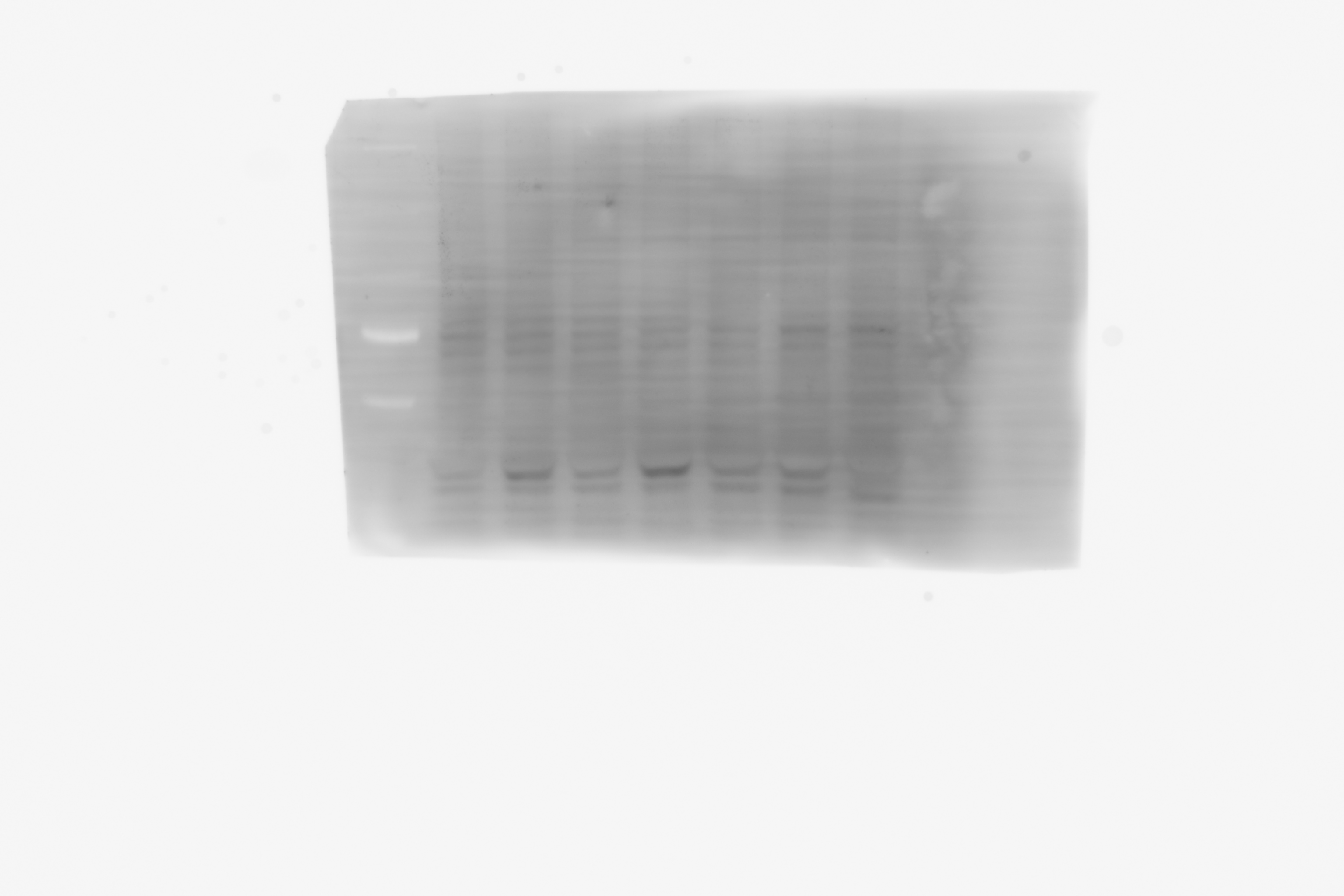

Supplement: Figure 5—source data 2. [file elife-106814-fig5-data2.zip › Figure 5-source data 2/Figure 5I_pcJun.tif]

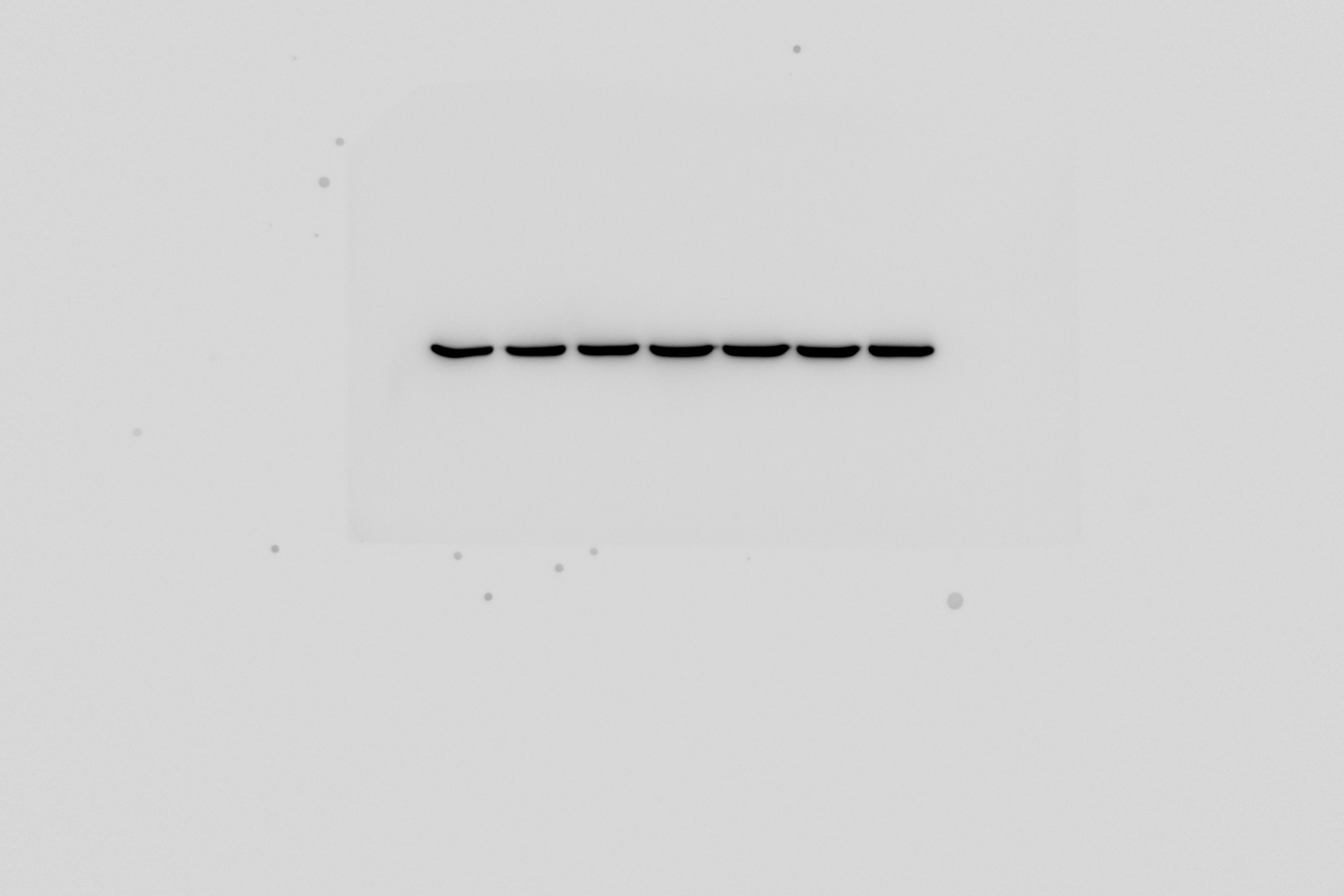

Supplement: Figure 5—source data 2. [file elife-106814-fig5-data2.zip › Figure 5-source data 2/Figure 5I_b-tubulin.tif]

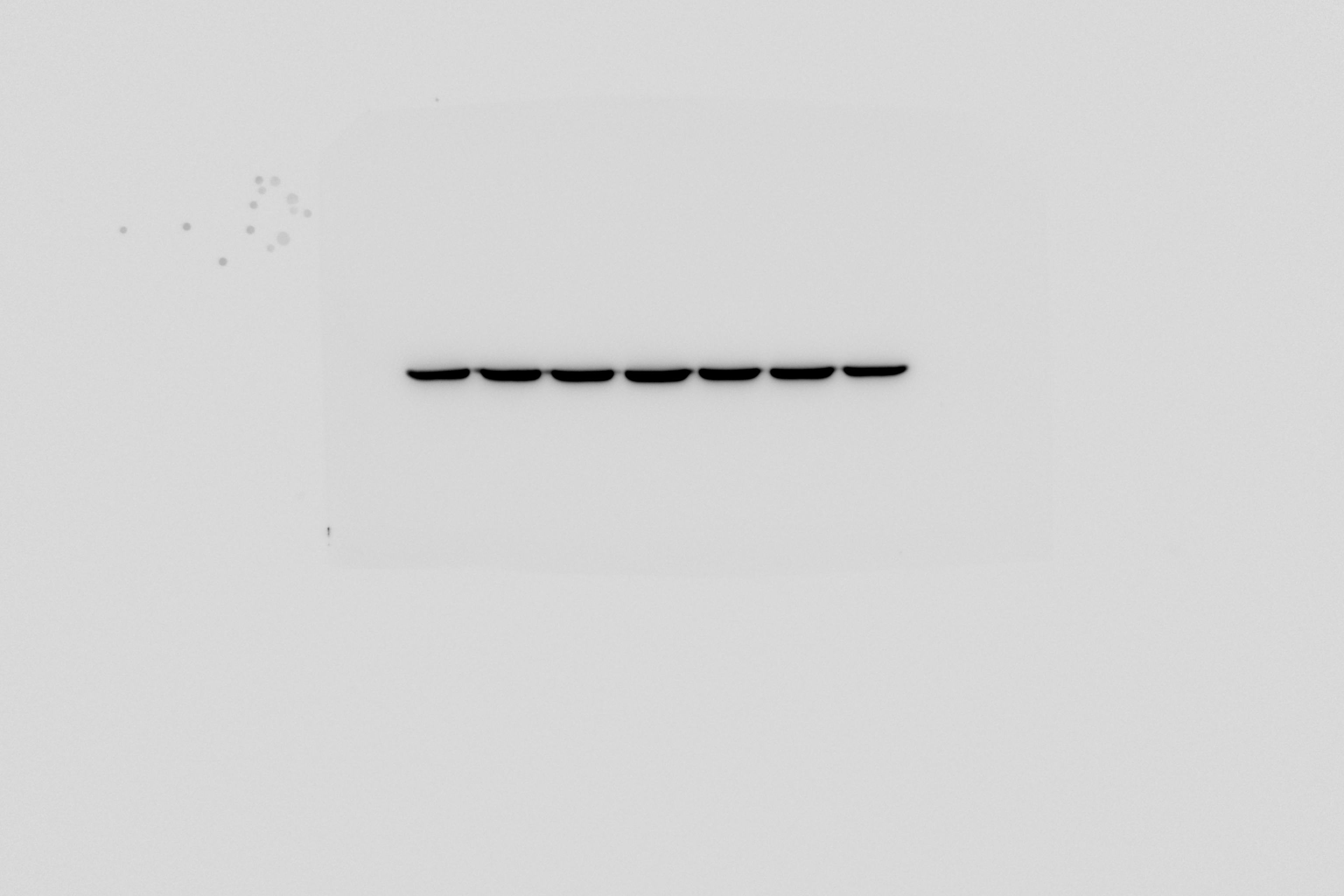

Supplement: Figure 5—source data 2. [file elife-106814-fig5-data2.zip › Figure 5-source data 2/Figure 5J_b-tubulin.tif]

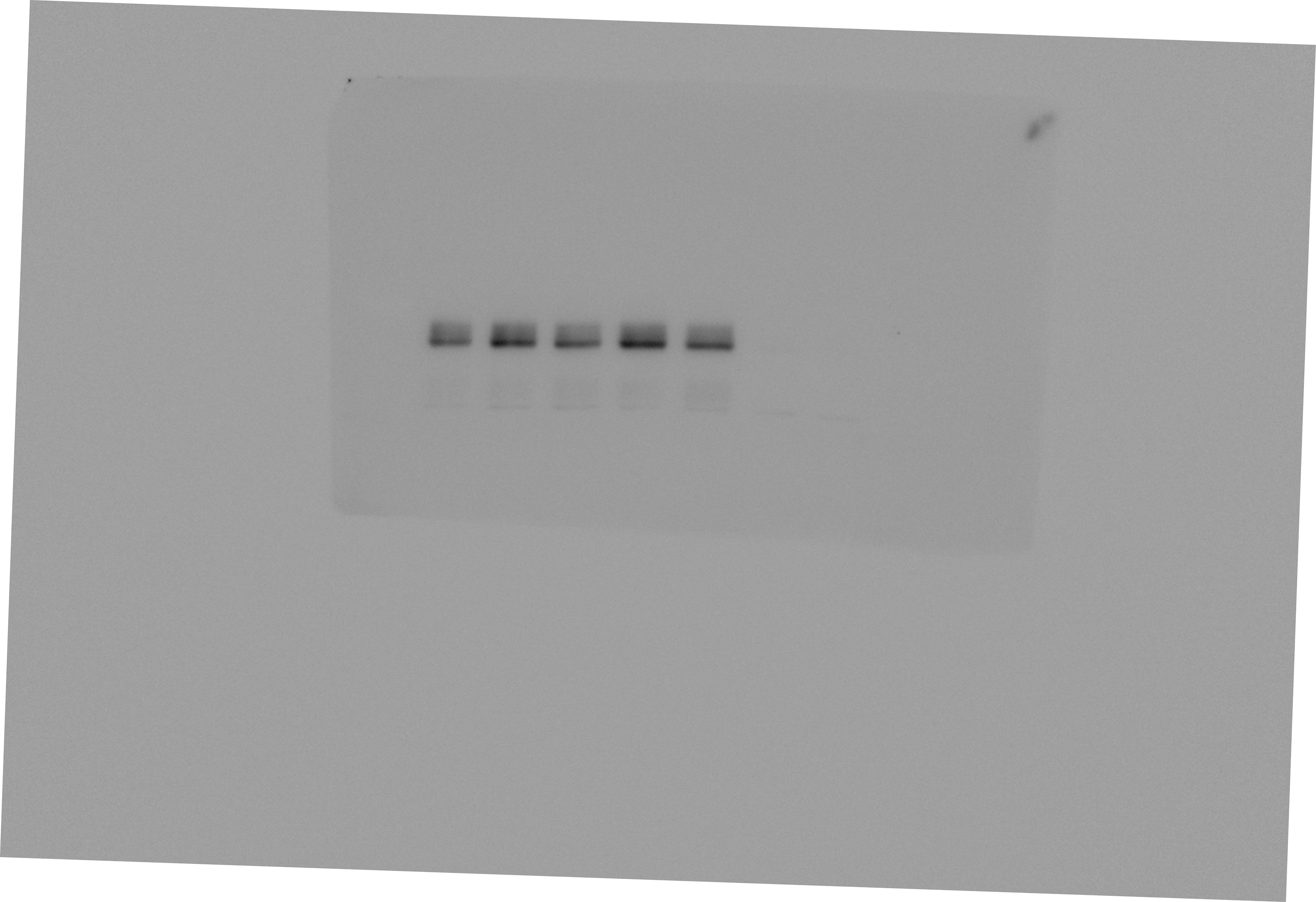

Supplement: Figure 5—source data 2. [file elife-106814-fig5-data2.zip › Figure 5-source data 2/Figure 5I_c-myc.tif]

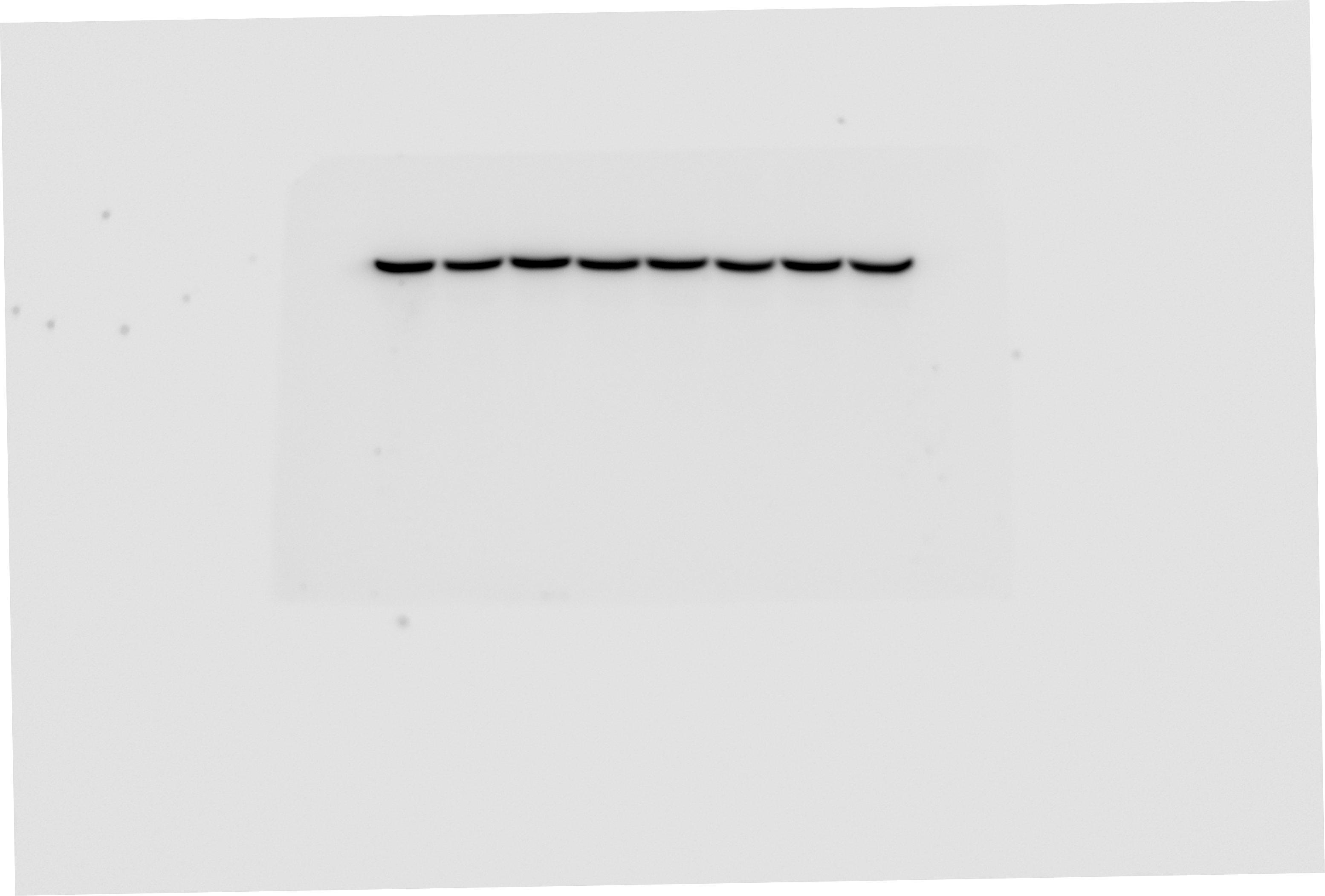

Supplement: Figure 5—source data 2. [file elife-106814-fig5-data2.zip › Figure 5-source data 2/Figure 5B_b-tubulin (12h).tif]

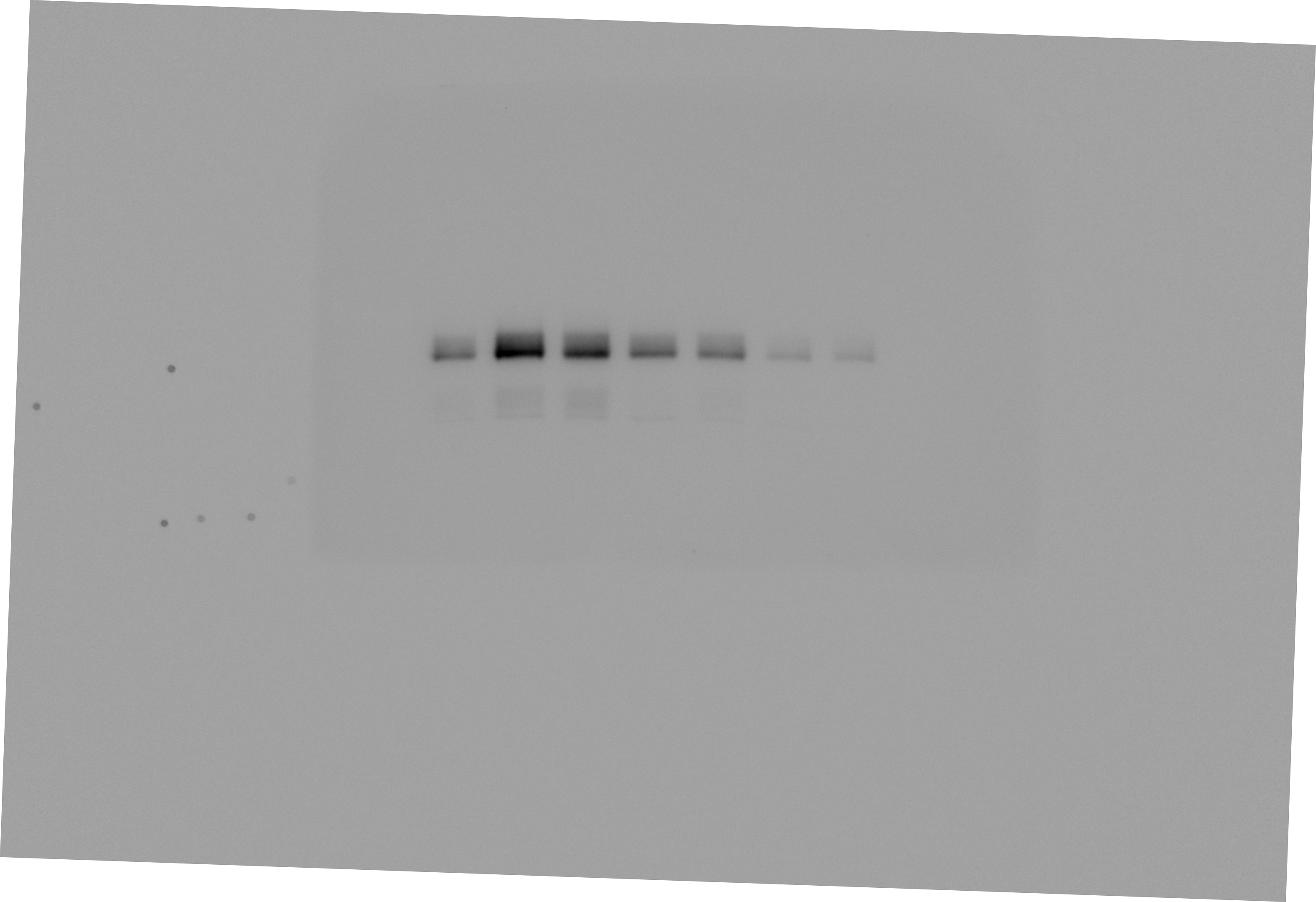

Supplement: Figure 5—source data 2. [file elife-106814-fig5-data2.zip › Figure 5-source data 2/Figure 5J_c-Myc.tif]

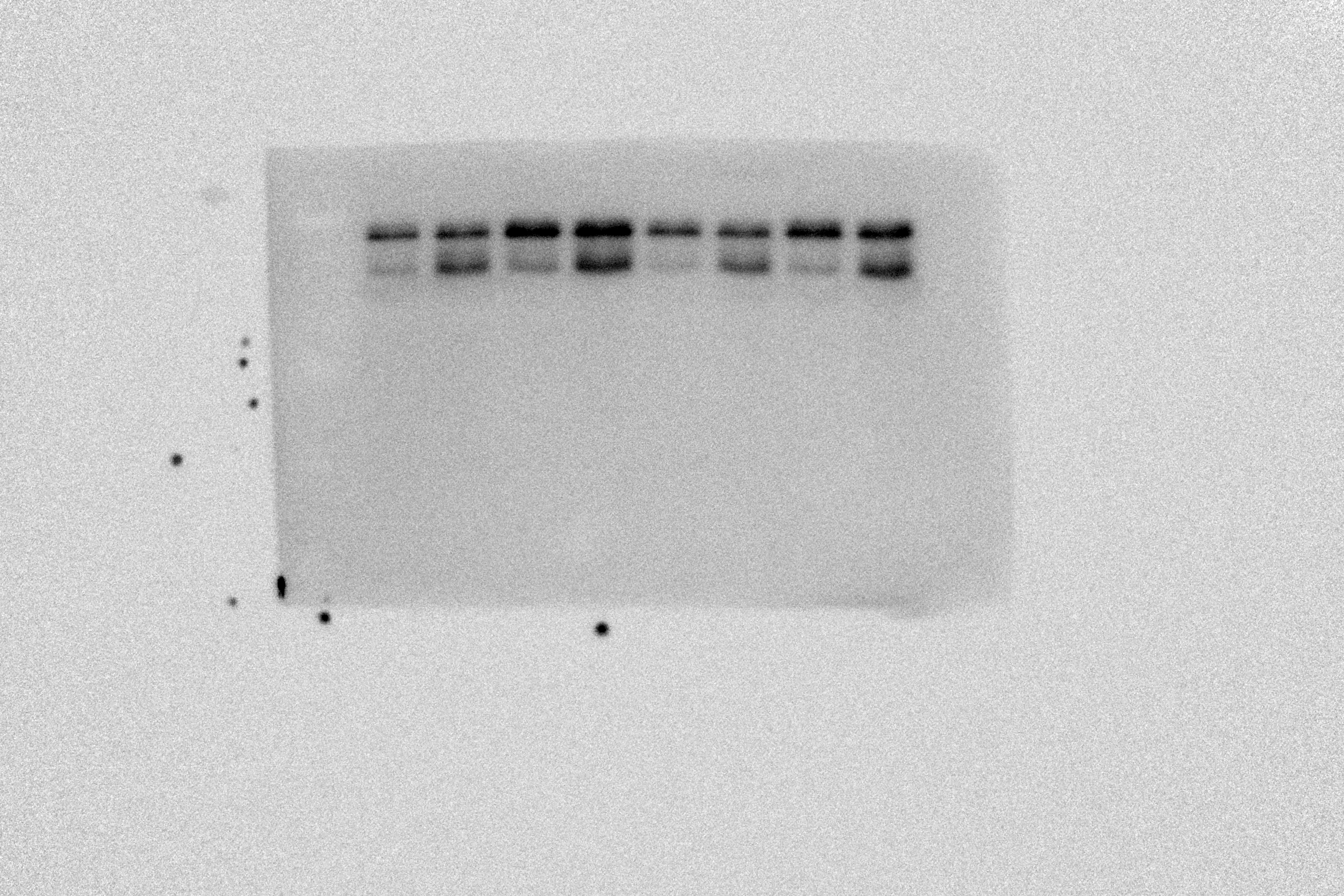

Supplement: Figure 5—source data 2. [file elife-106814-fig5-data2.zip › Figure 5-source data 2/Figure 5B_c-Myc (6h).tif]

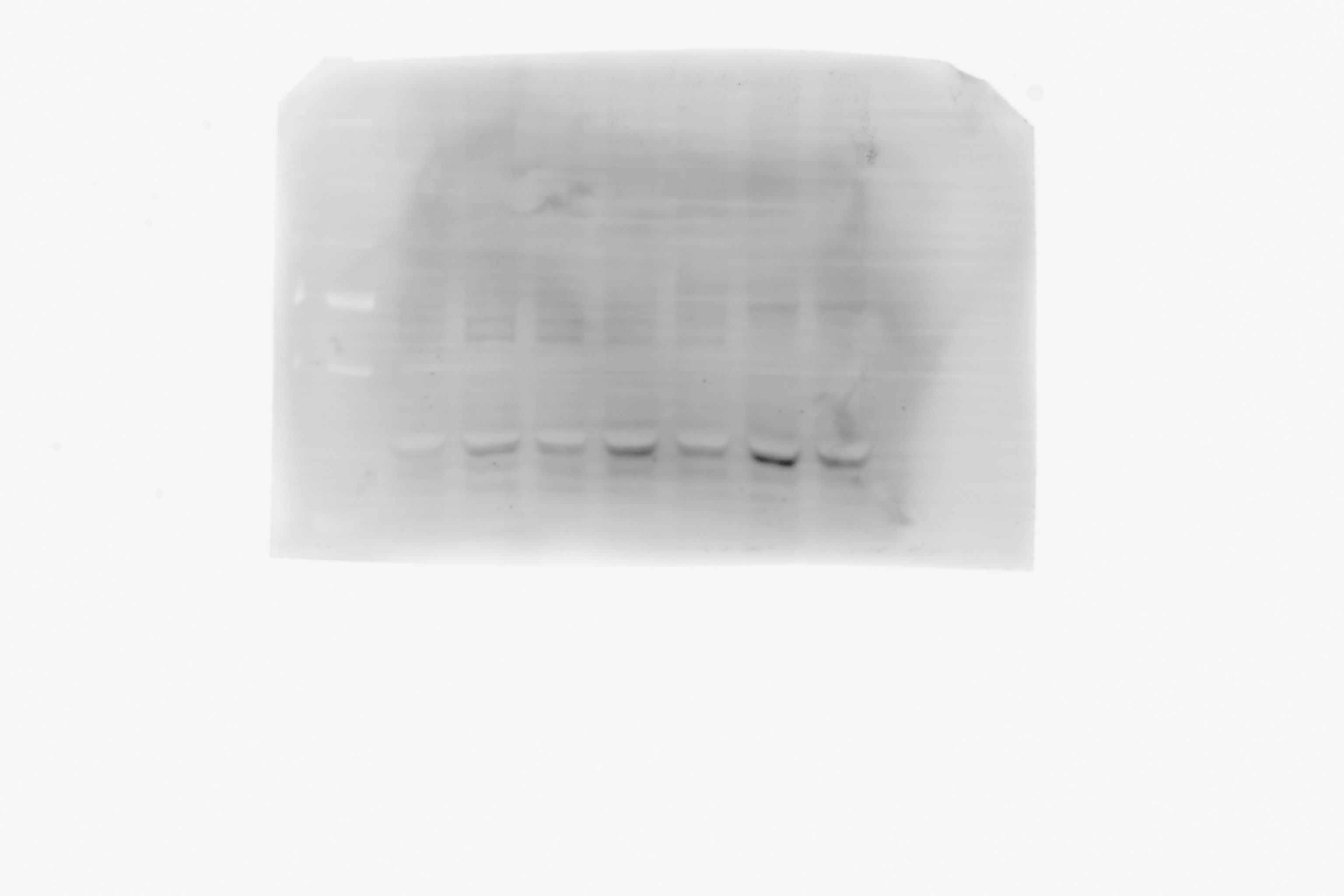

Supplement: Figure 5—source data 2. [file elife-106814-fig5-data2.zip › Figure 5-source data 2/Figure 5J_pcJun.tif]

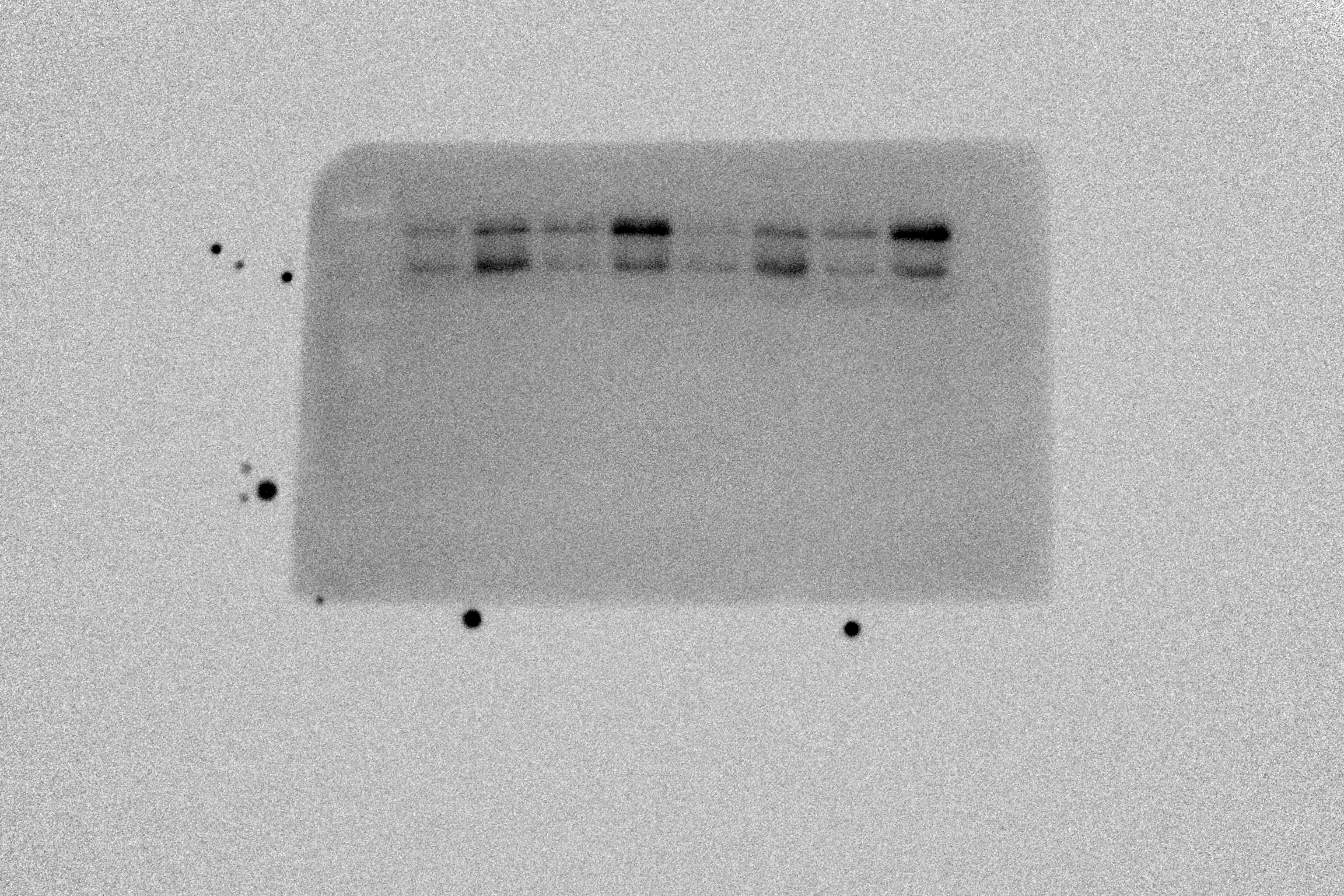

Supplement: Figure 5—source data 2. [file elife-106814-fig5-data2.zip › Figure 5-source data 2/Figure 5B_c-Myc (12h).tif]

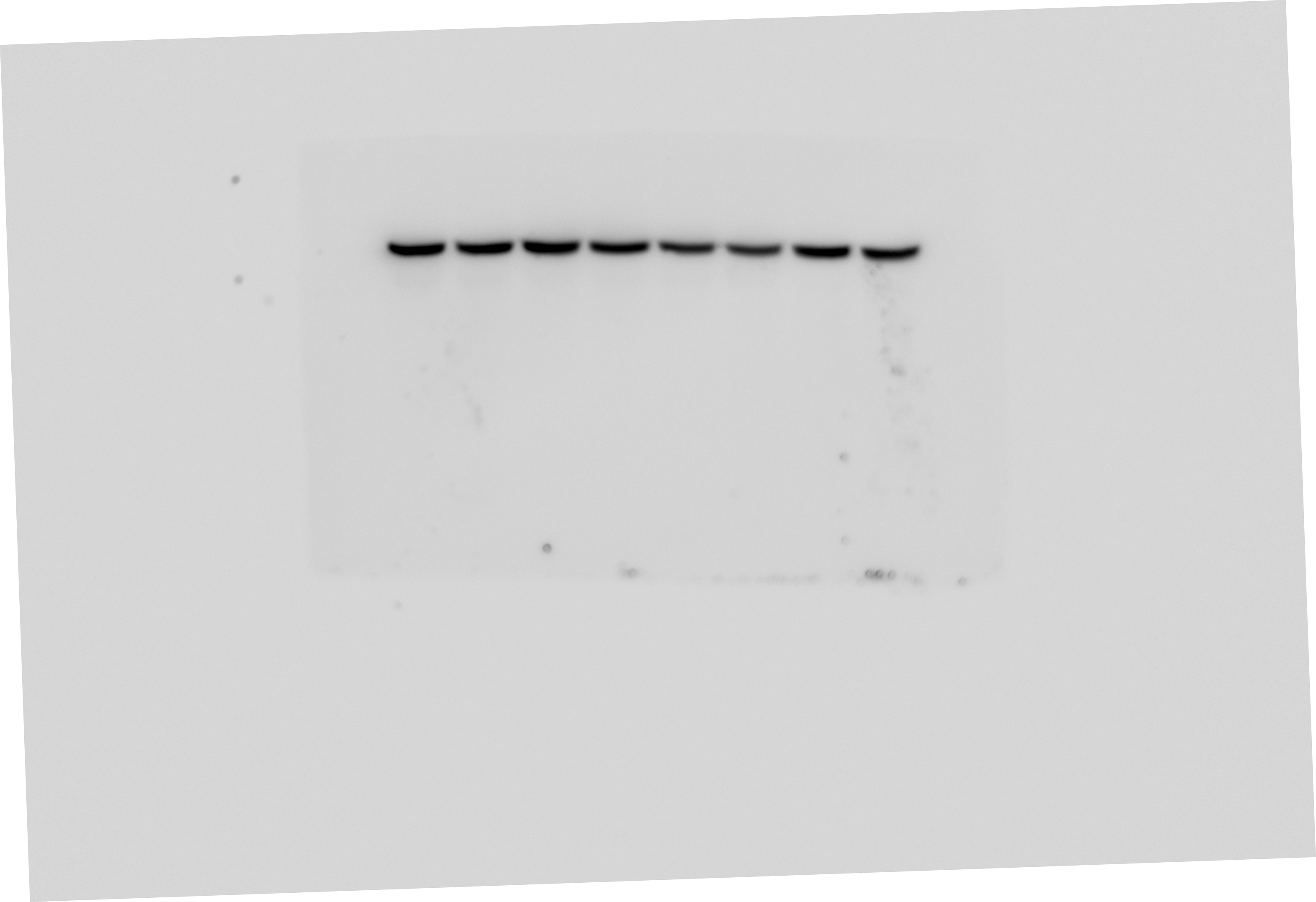

Supplement: Figure 5—source data 2. [file elife-106814-fig5-data2.zip › Figure 5-source data 2/Figure 5B_b-tubulin (6h).tif]
