# Supplementary material for: Lipid peroxidation and type I interferon coupling fuels pathogenic macrophage activation causing tuberculosis susceptibility
Source: eLife. 2025 Oct 2;14:RP106814. doi: 10.7554/eLife.106814 (PMC12490860; doi:10.7554/eLife.106814)

Figure 2-figure supplement 1 A: β-TrCP

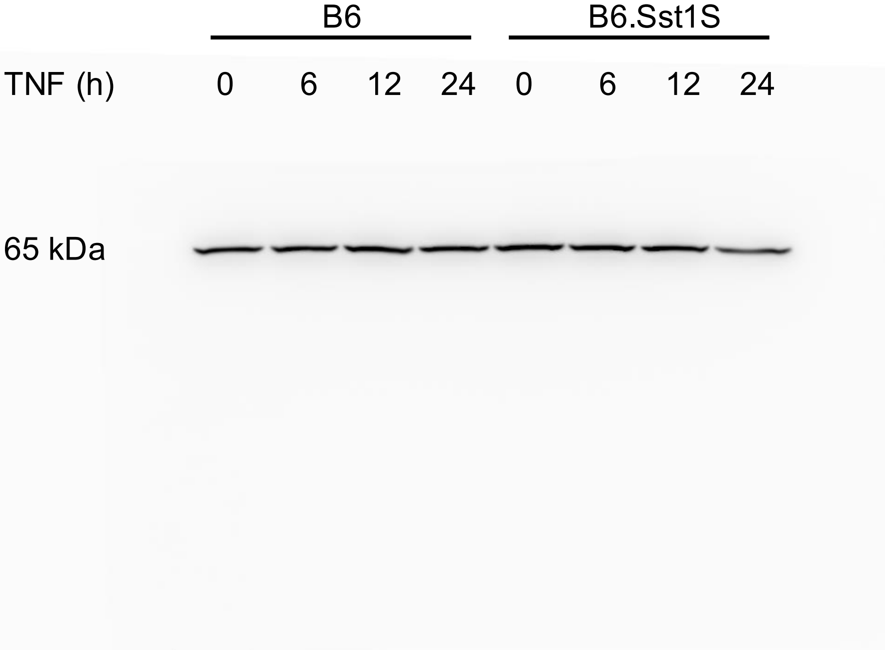

Figure 2-figure supplement 1 A: Keap1

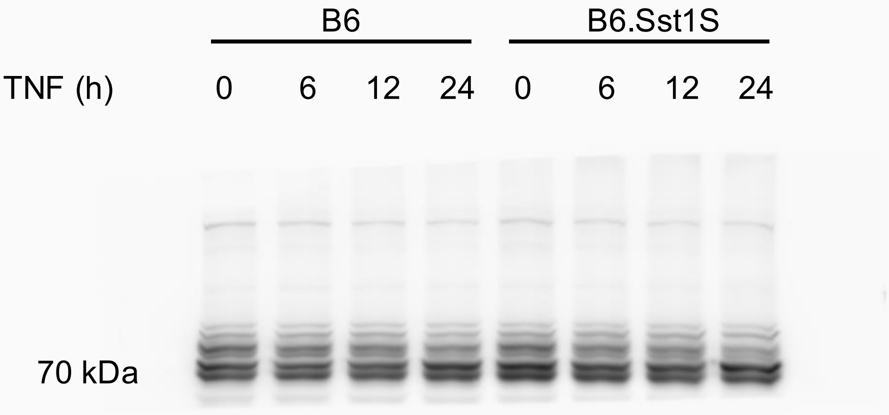

Figure 2-figure supplement 1 A: Nrf1

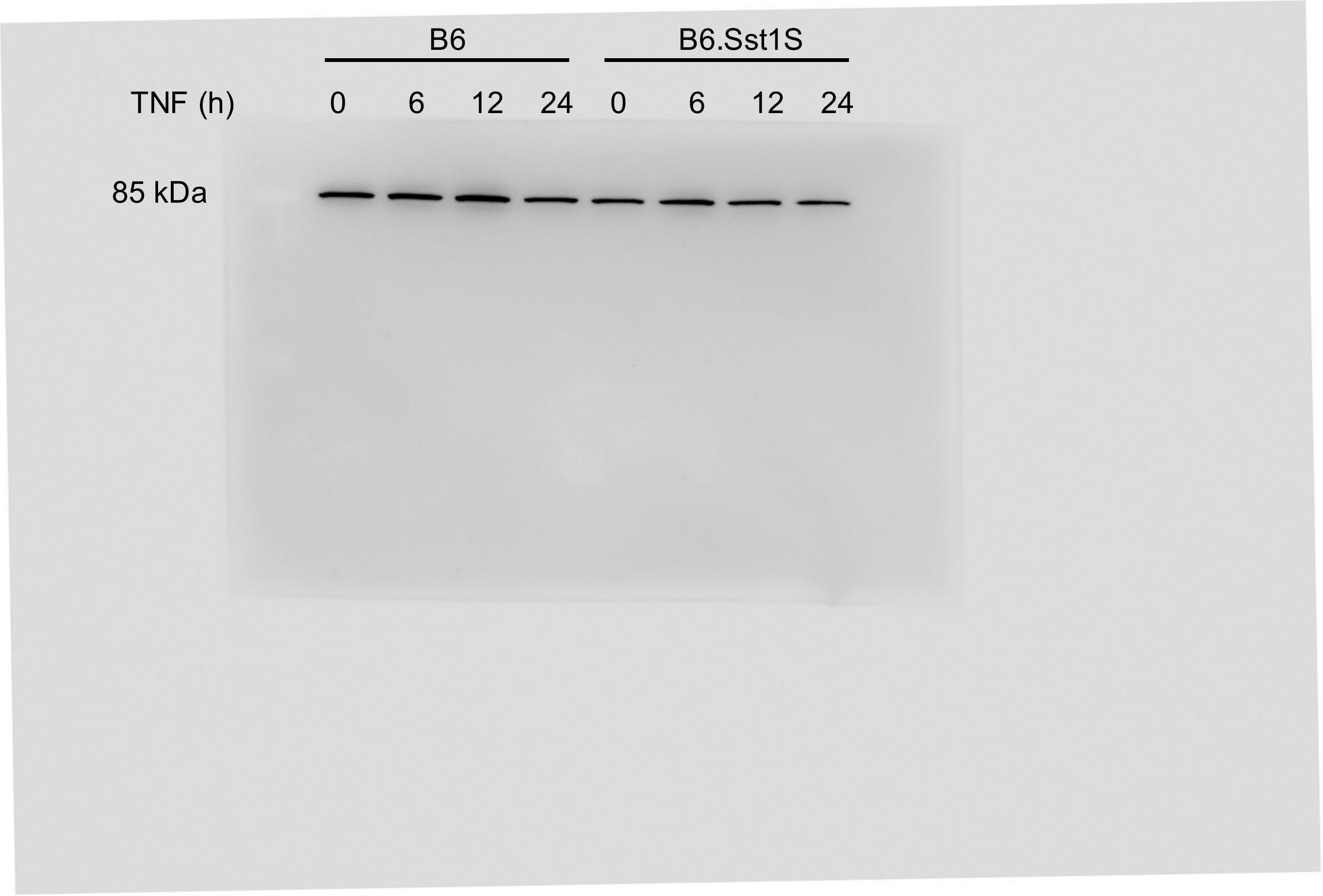

Figure 2-figure supplement 1 A: β-tubulin

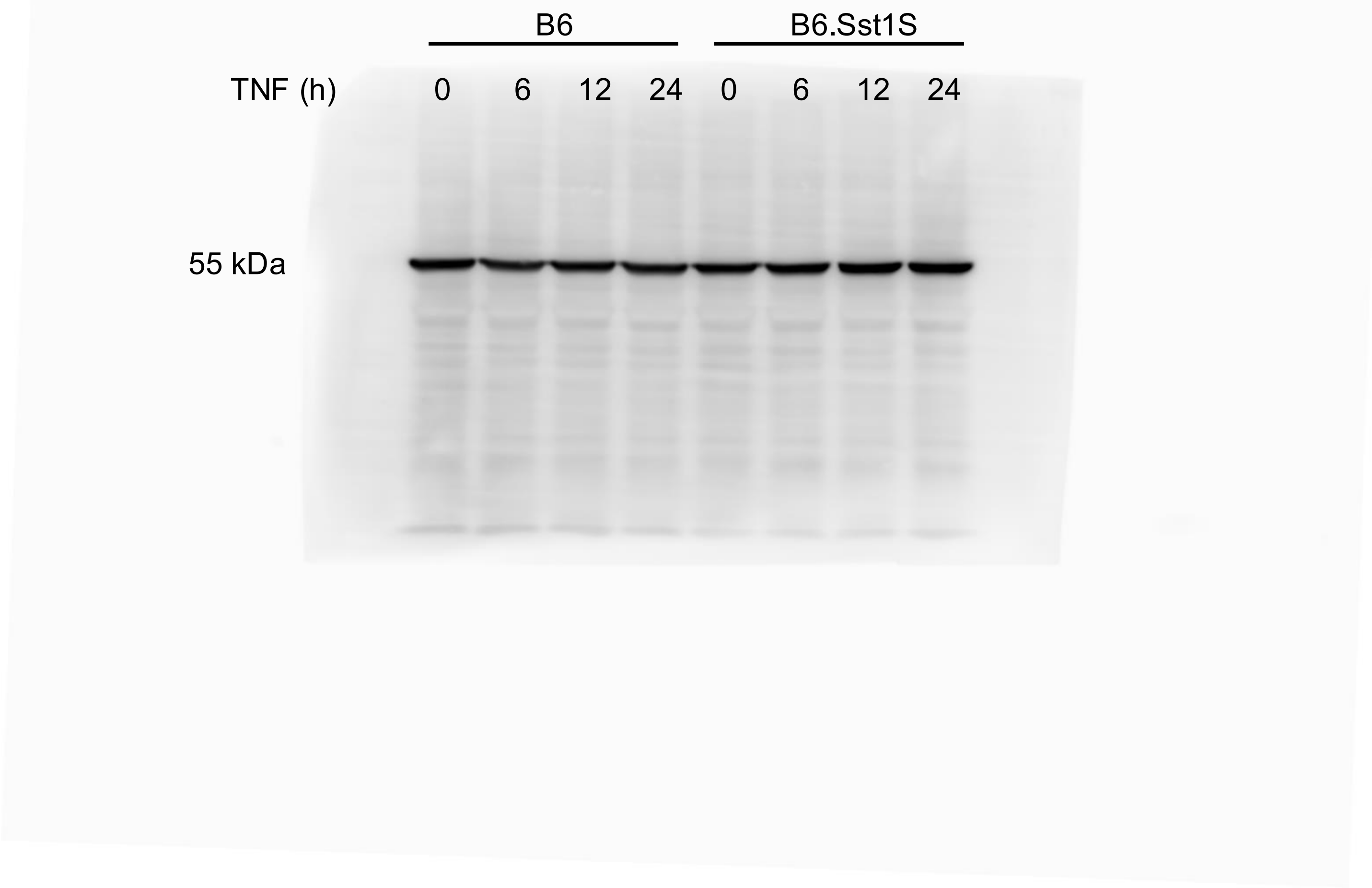

Supplement: Figure 2—figure supplement 1—source data 1. [file elife-106814-fig2-figsupp1-data1.zip › Figure 2-figure suppplement 1-source data 1.pdf]
