# Supplementary material for: Lipid peroxidation and type I interferon coupling fuels pathogenic macrophage activation causing tuberculosis susceptibility
Source: eLife. 2025 Oct 2;14:RP106814. doi: 10.7554/eLife.106814 (PMC12490860; doi:10.7554/eLife.106814)

Figure 3C: Fth

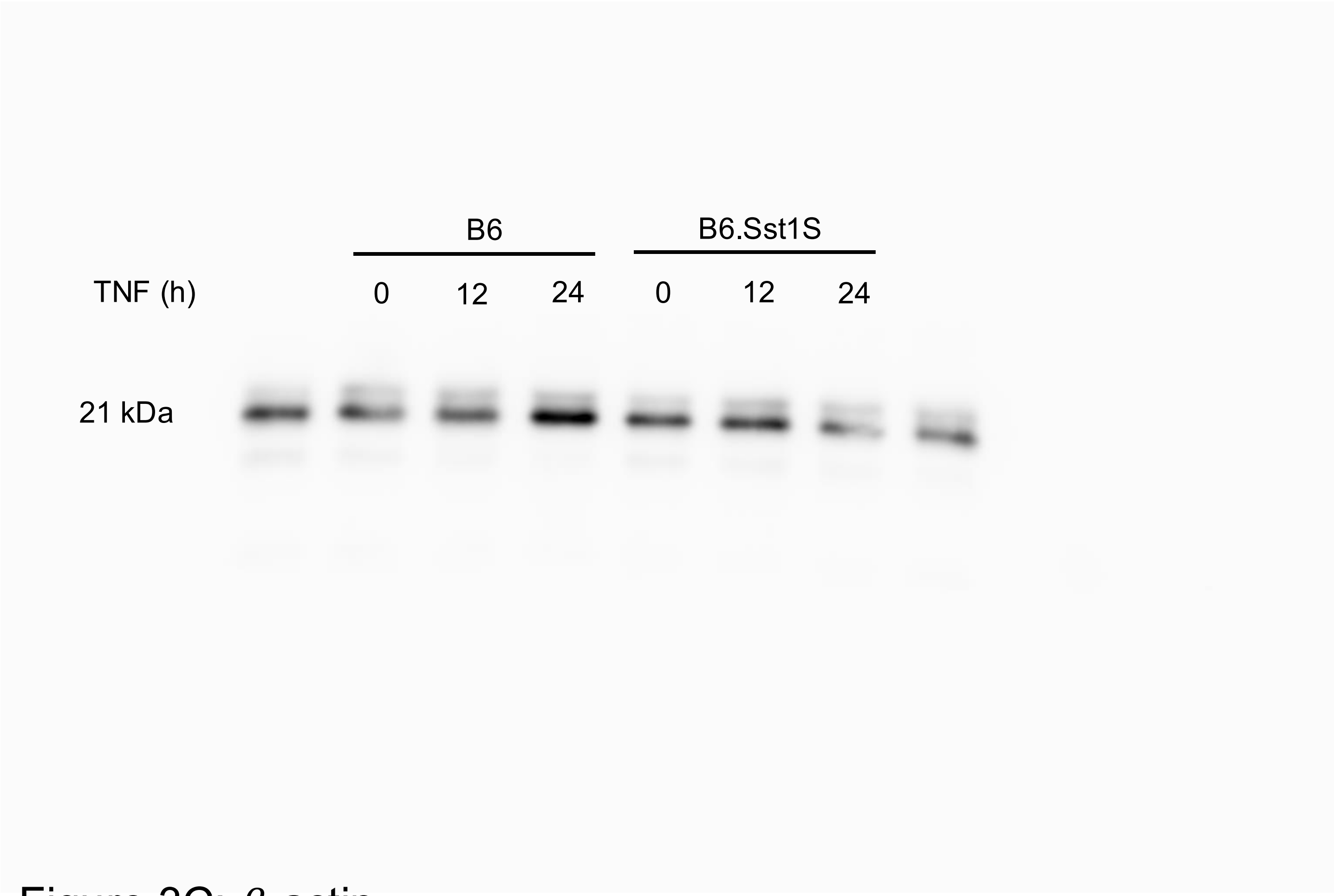

Figure 3C: β-actin

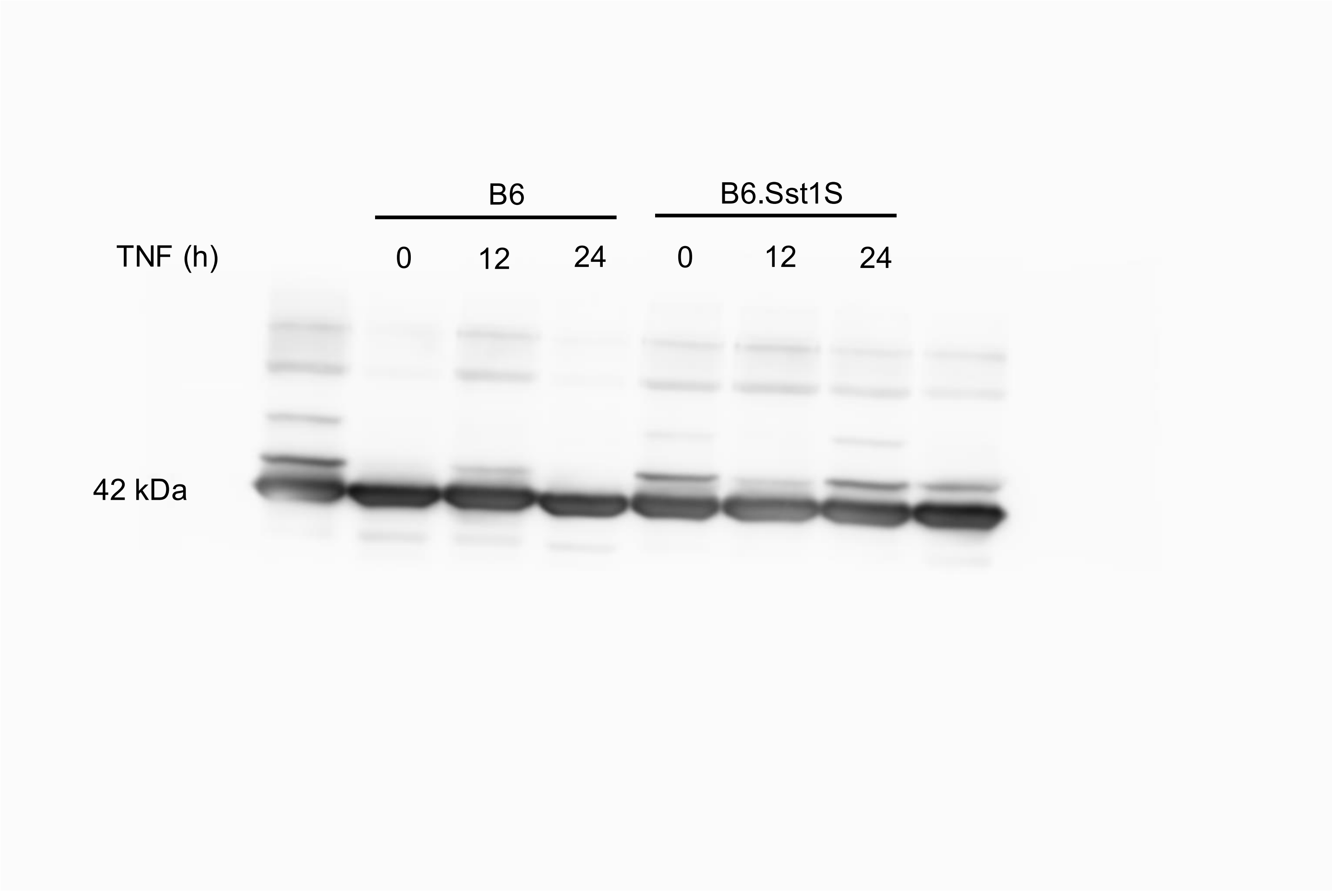

Figure 3C: Ftl

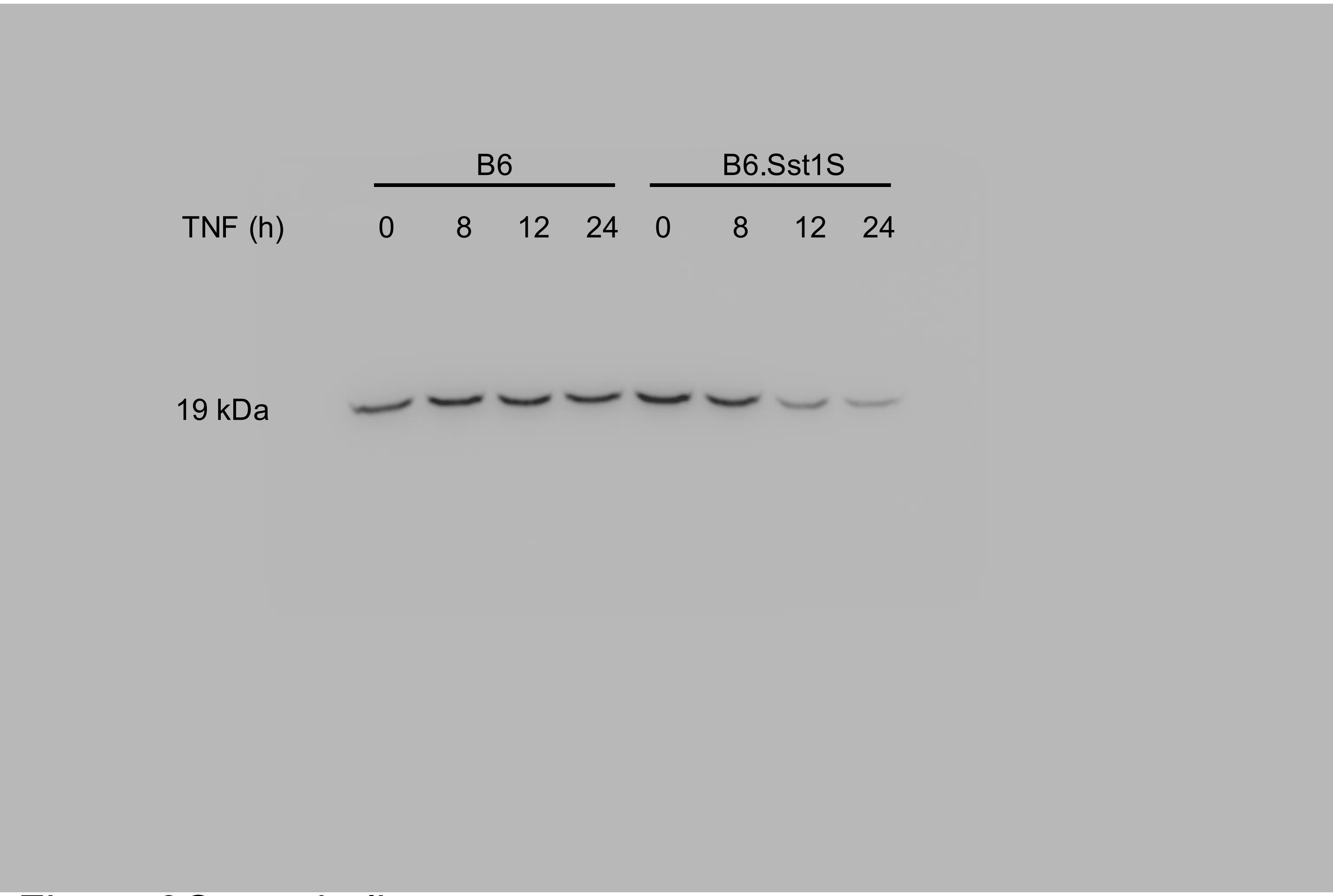

Figure 3C: β-tubulin

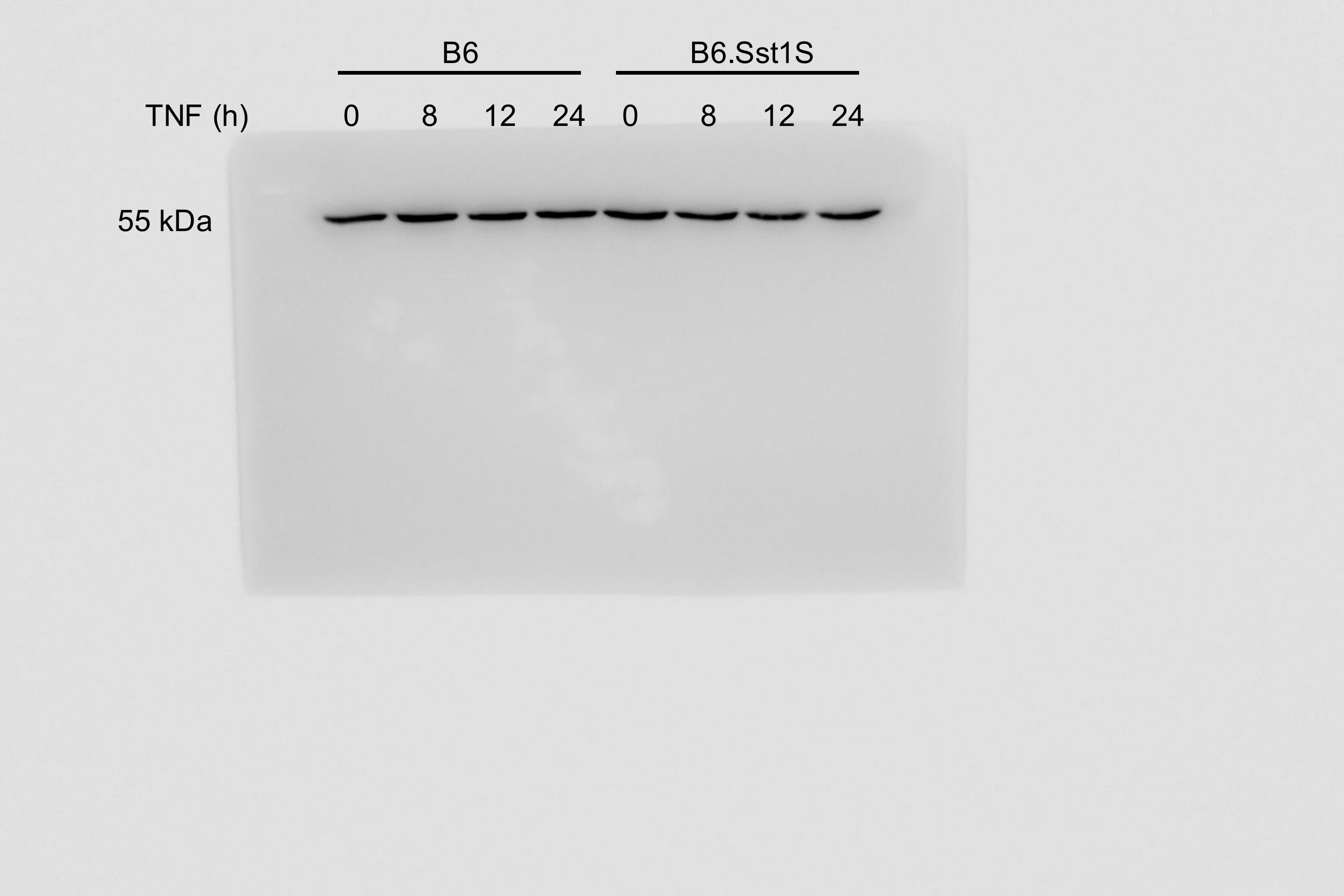

Figure 3D: Gpx1

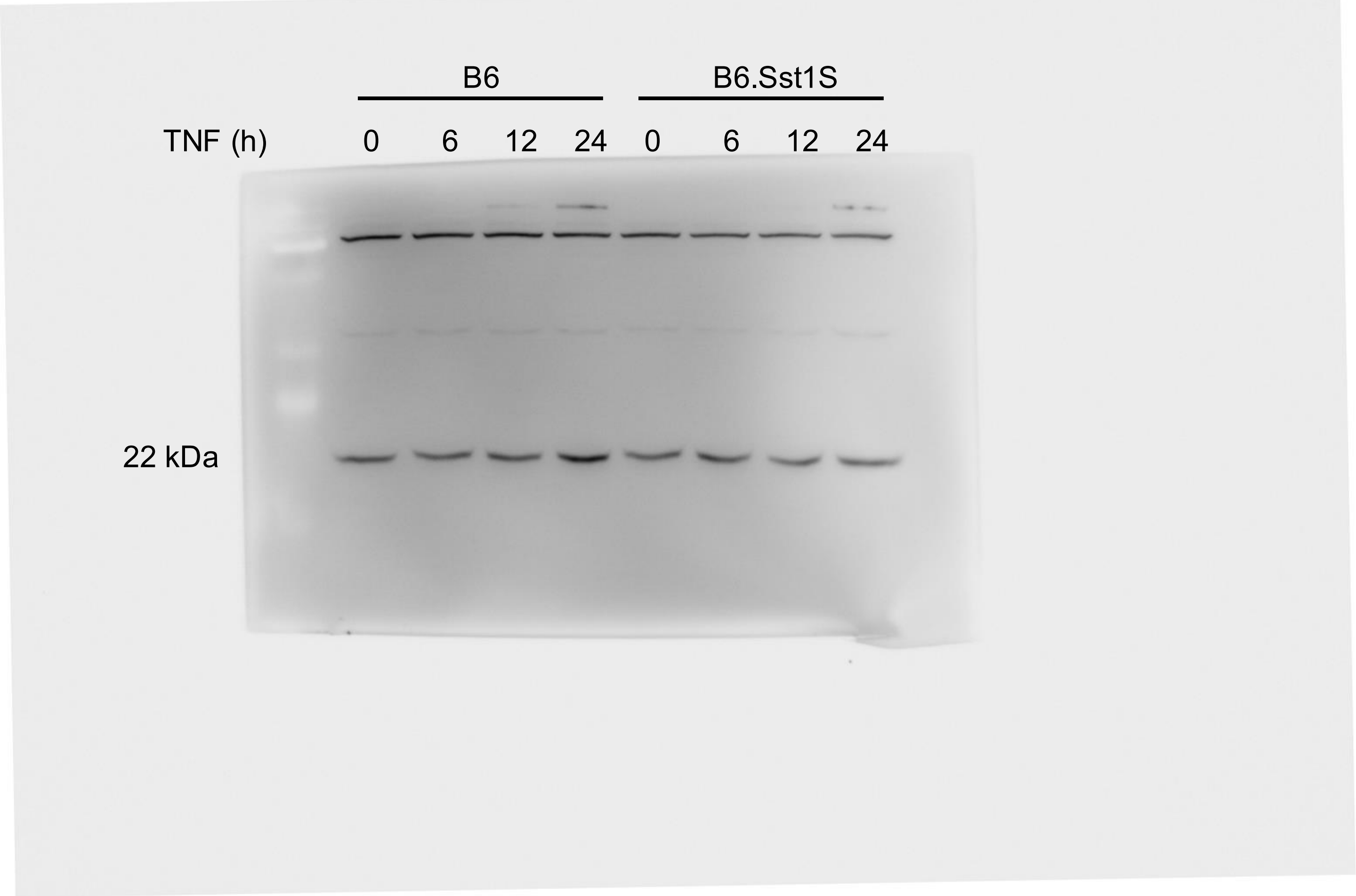

Figure 3D: β-tubulin

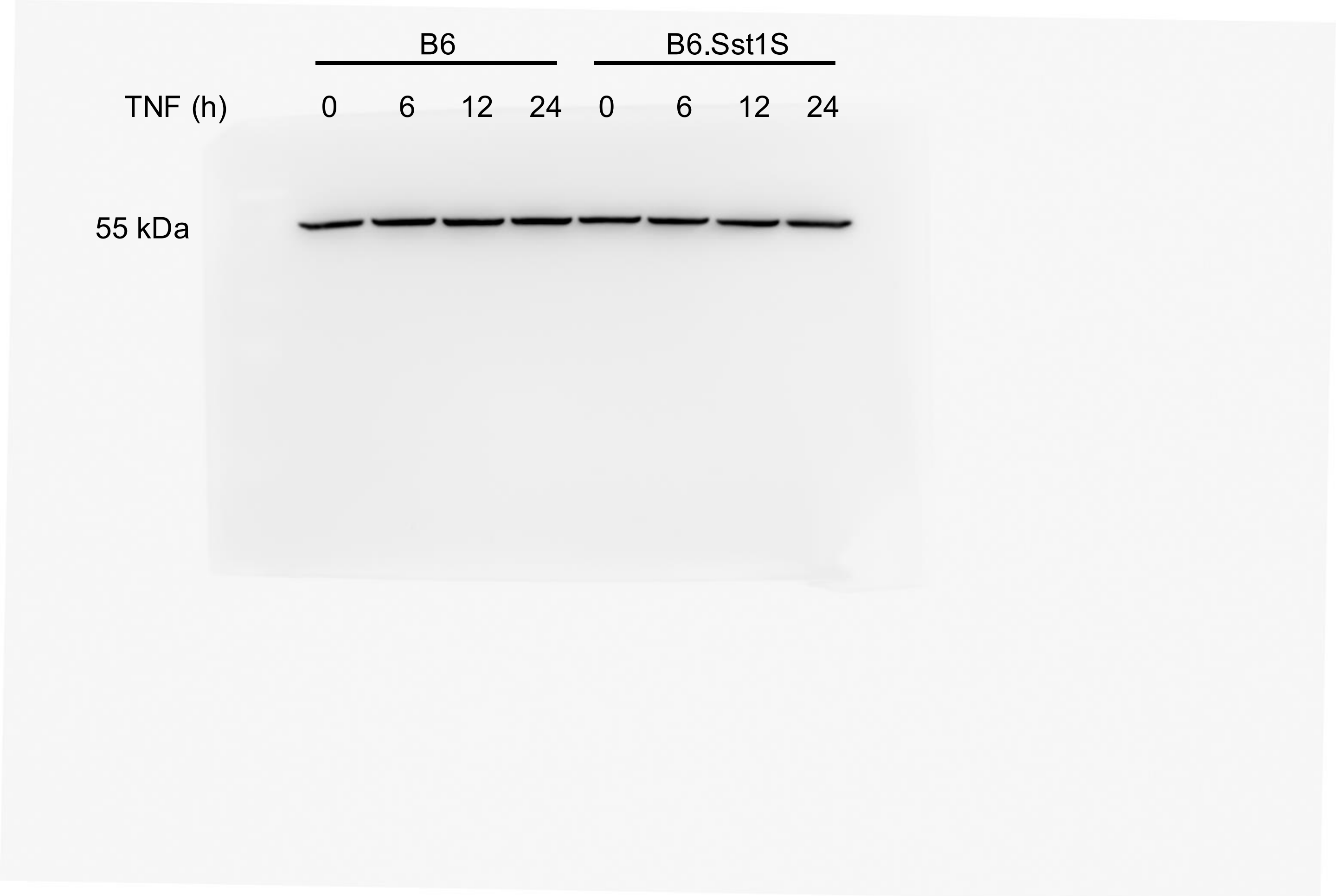

Figure 3D: Gpx4

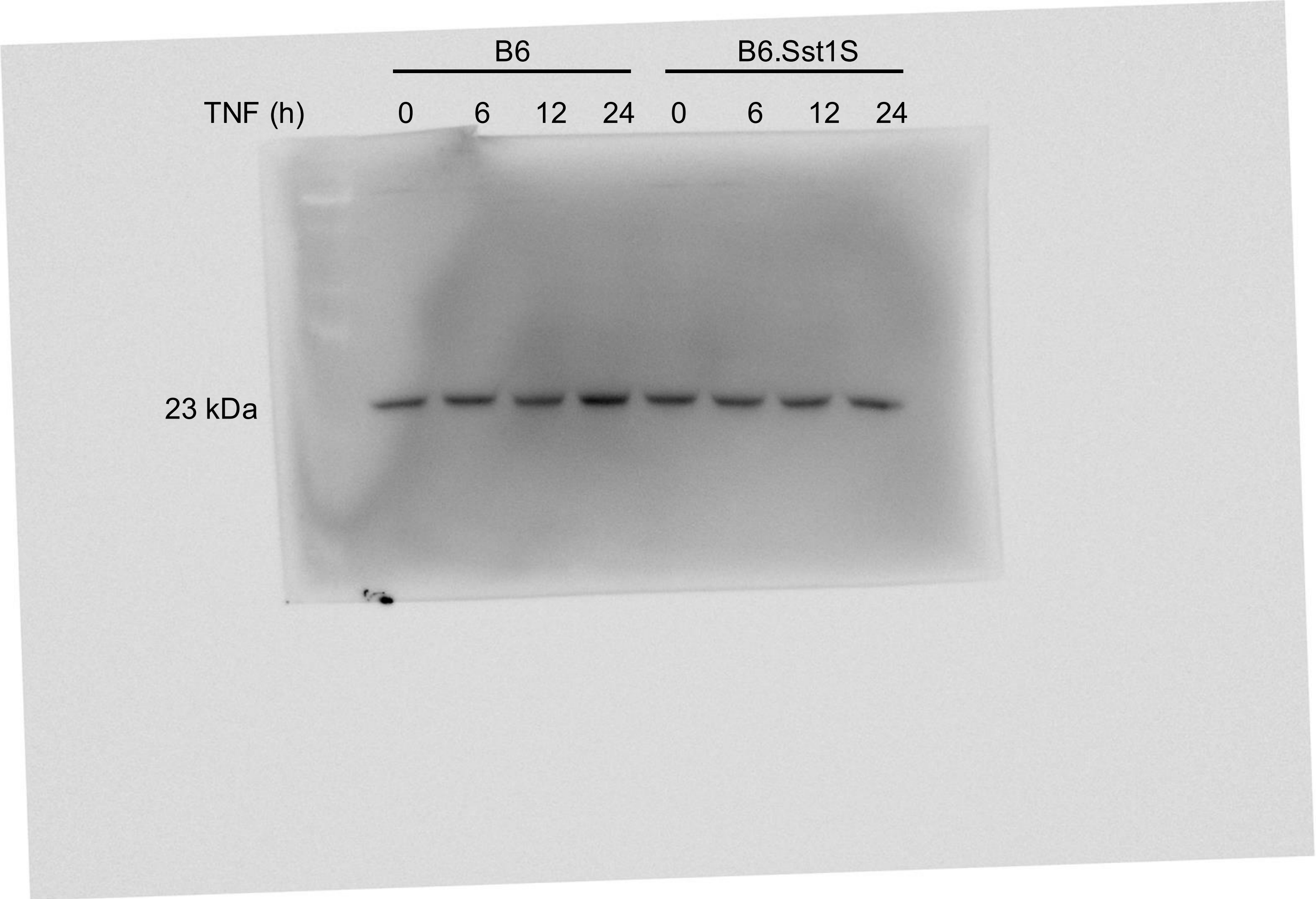

Figure 3D: β-tubulin

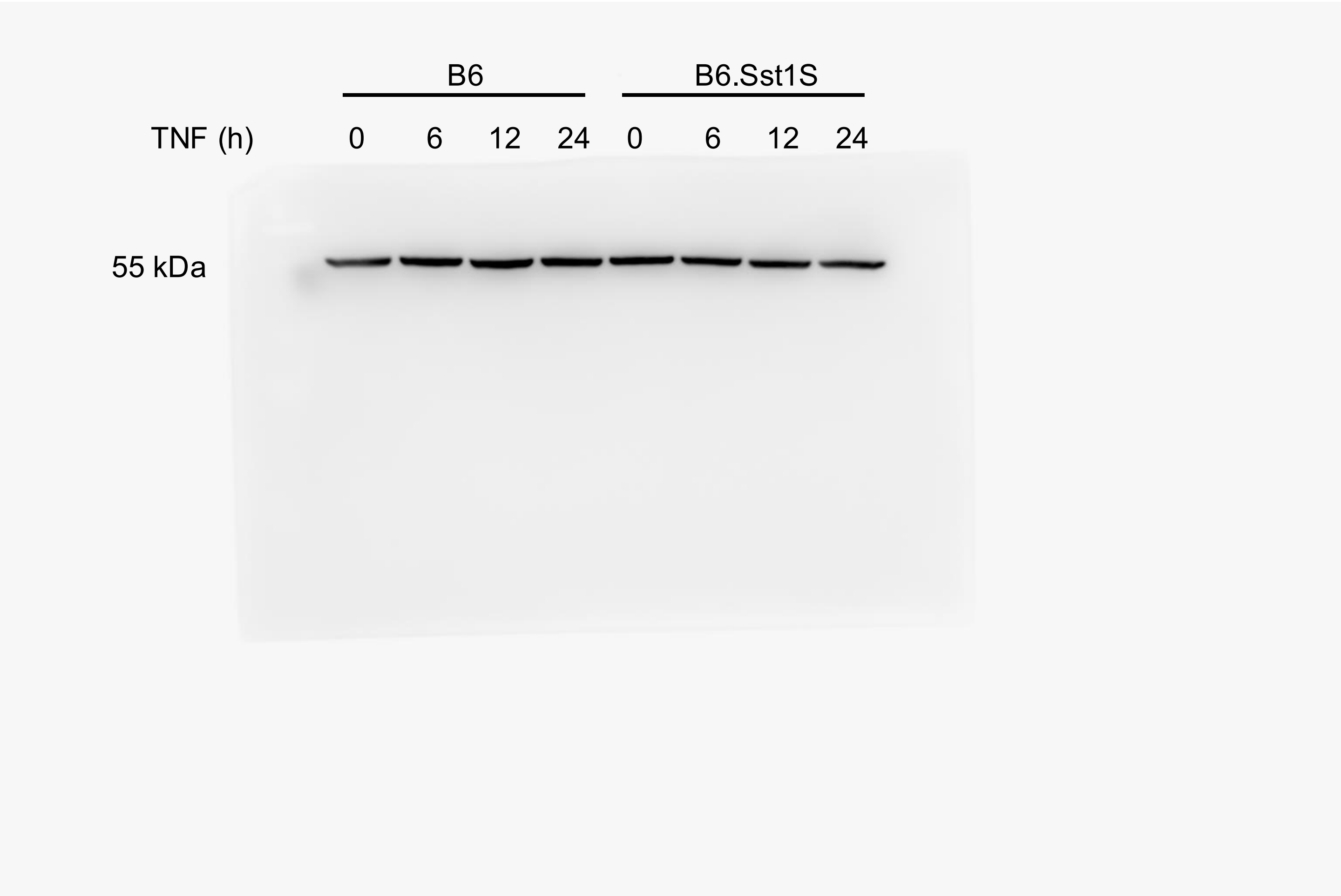

Figure 3K: p-cJun- 12 h

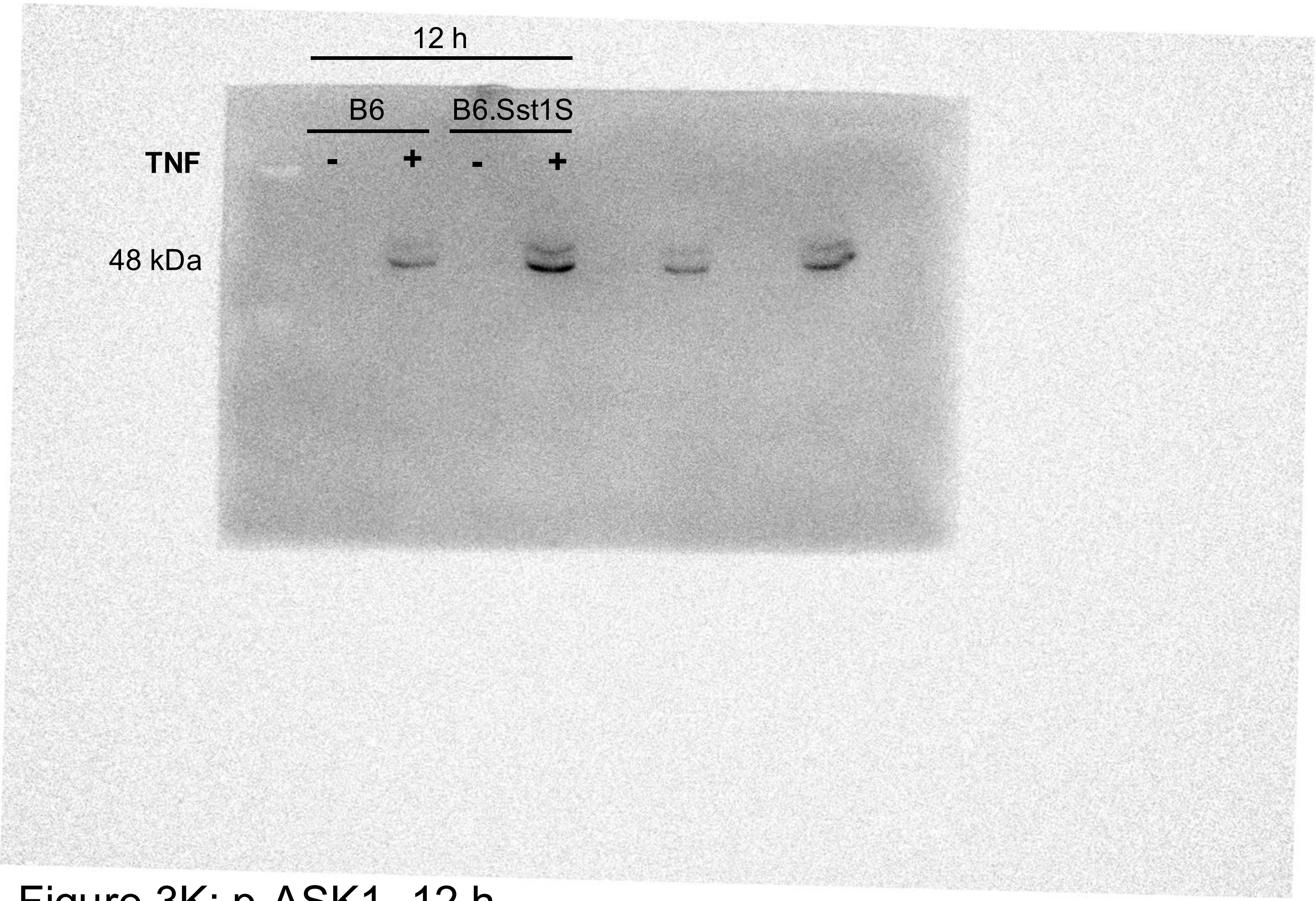

Figure 3K: p-ASK1- 12 h

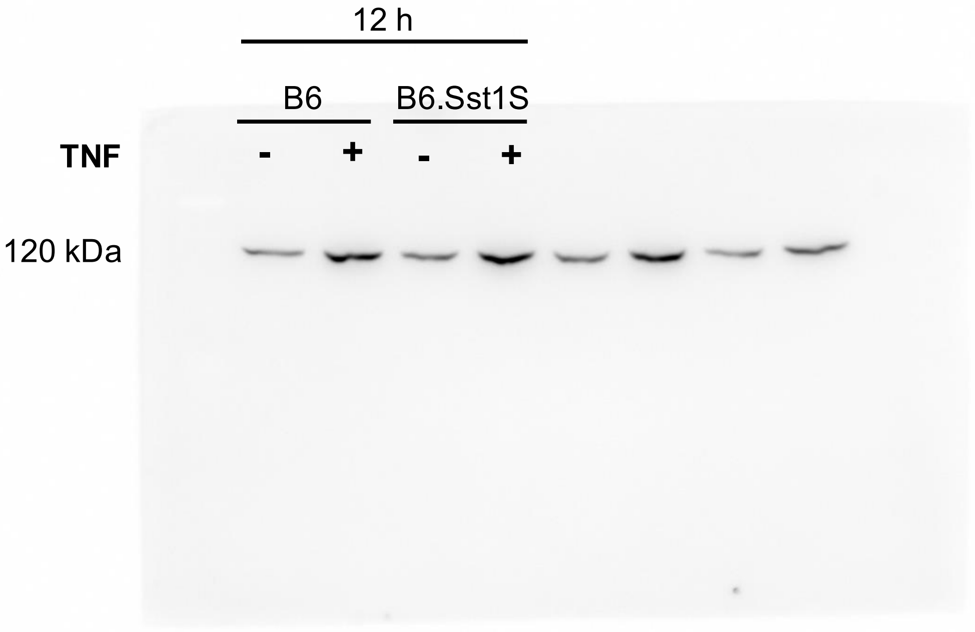

Figure 3K: β-tubulin- 12 h

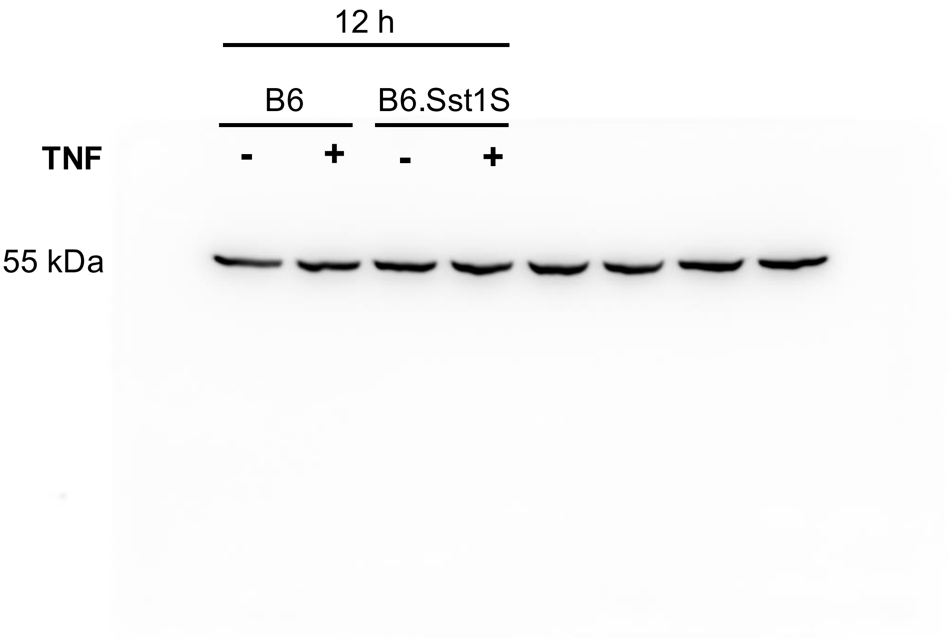

Figure 3K: p-cJun- 24 h

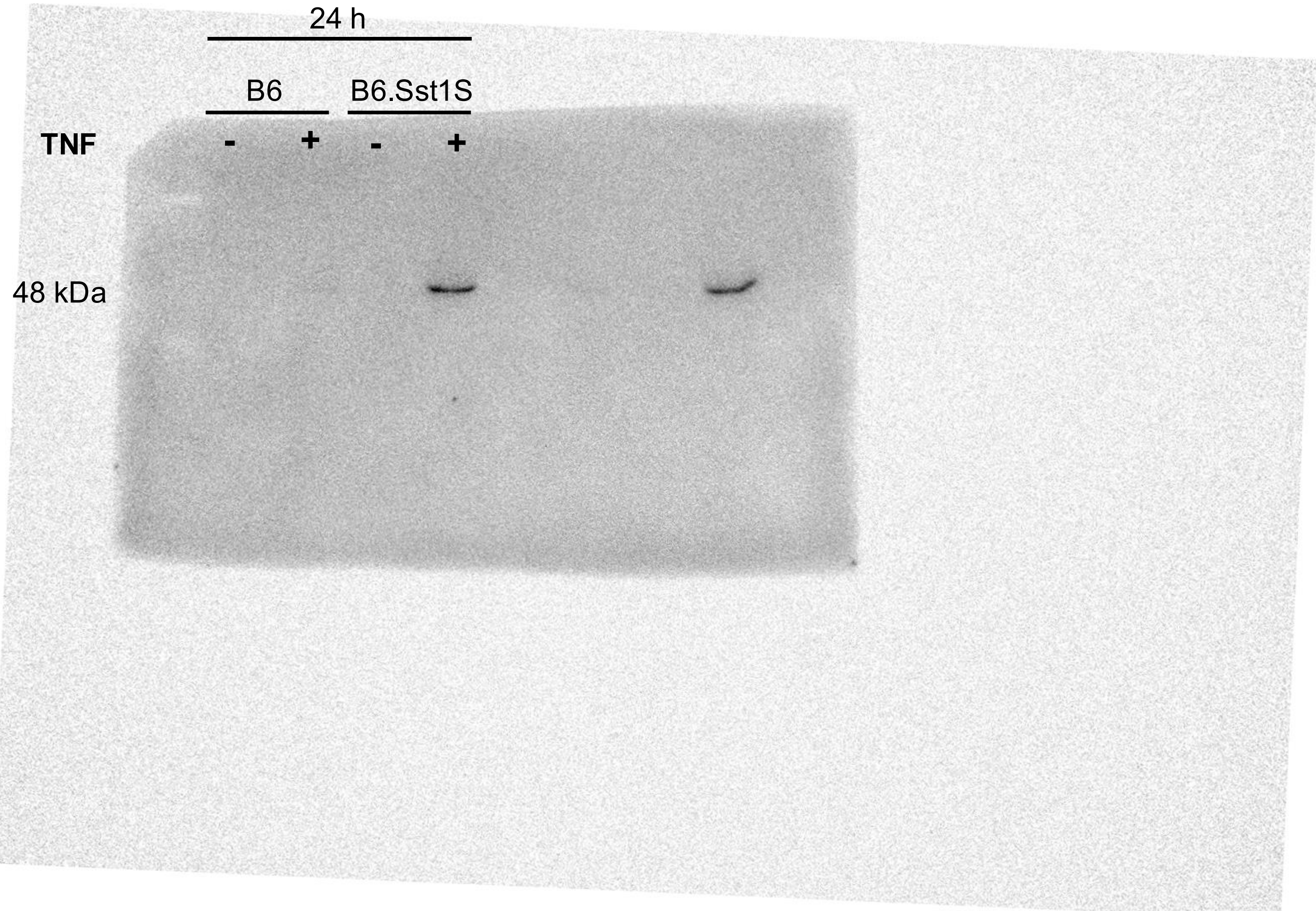

Figure 3K: p-ASK1- 24 h

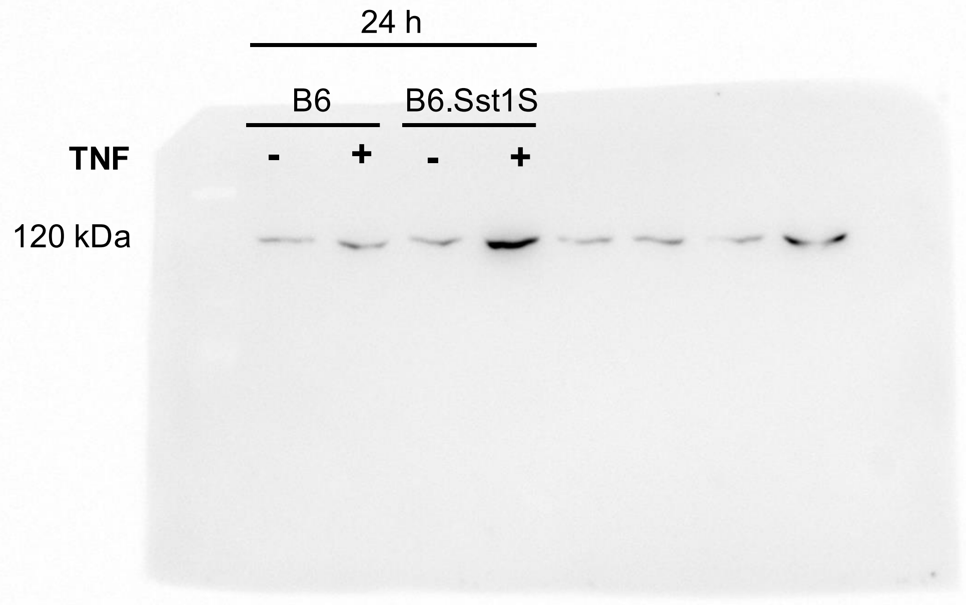

Figure 3K: β-tubulin- 24 h

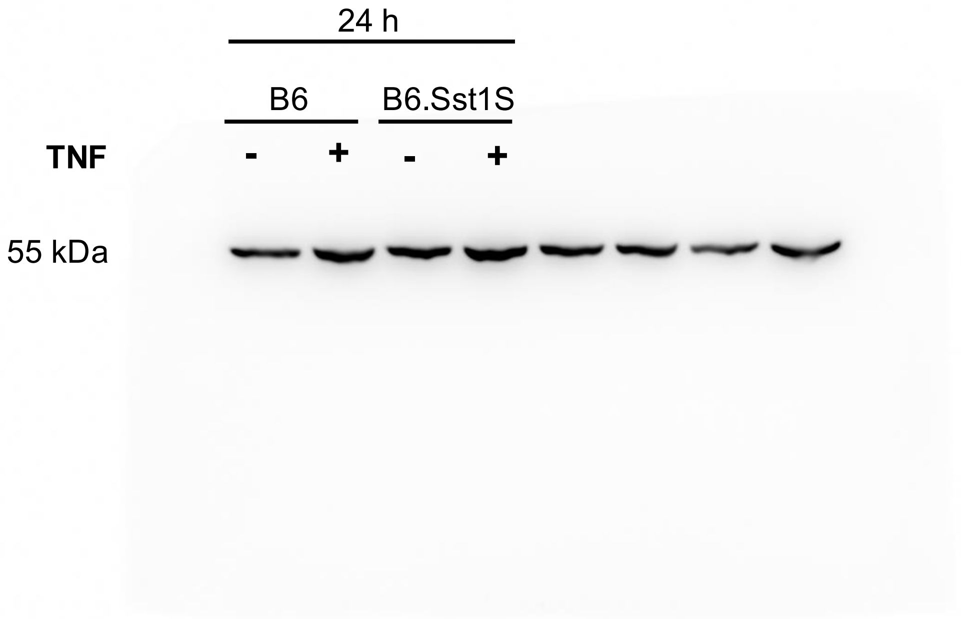

Figure 3K: p-cJun- 36 h

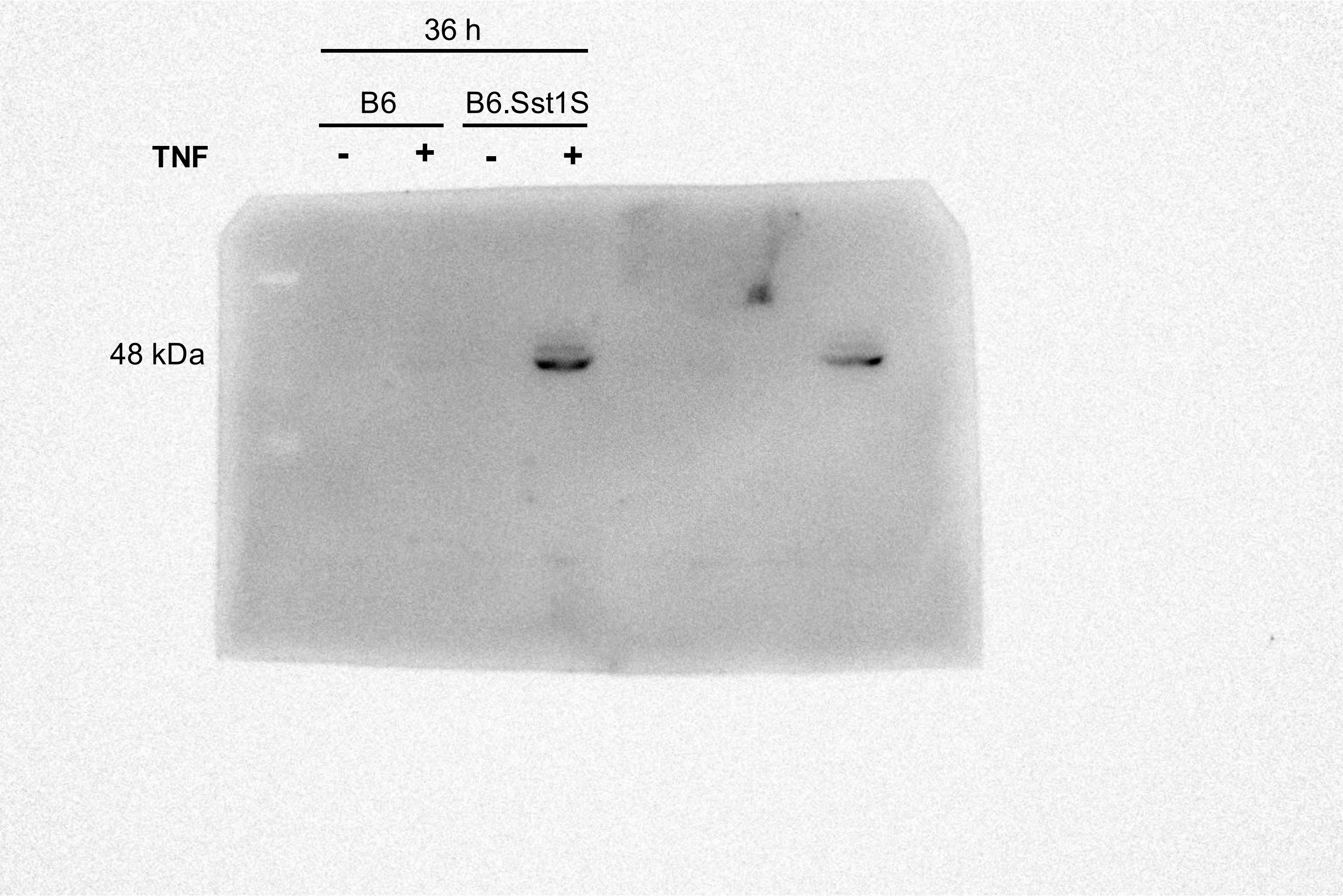

Figure 3K: p-ASK1- 36 h

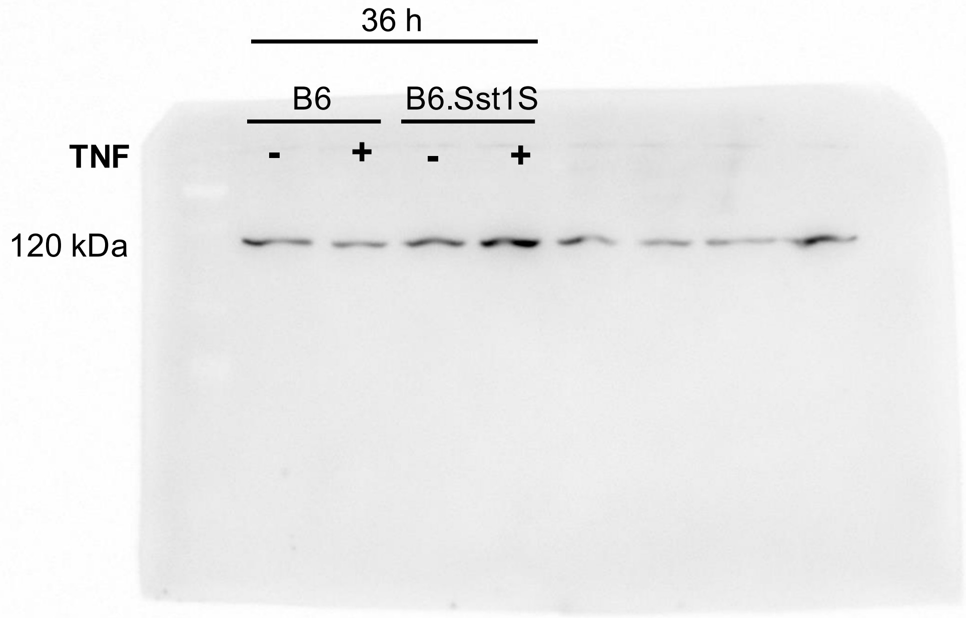

Figure 3K: β-tubulin- 36 h

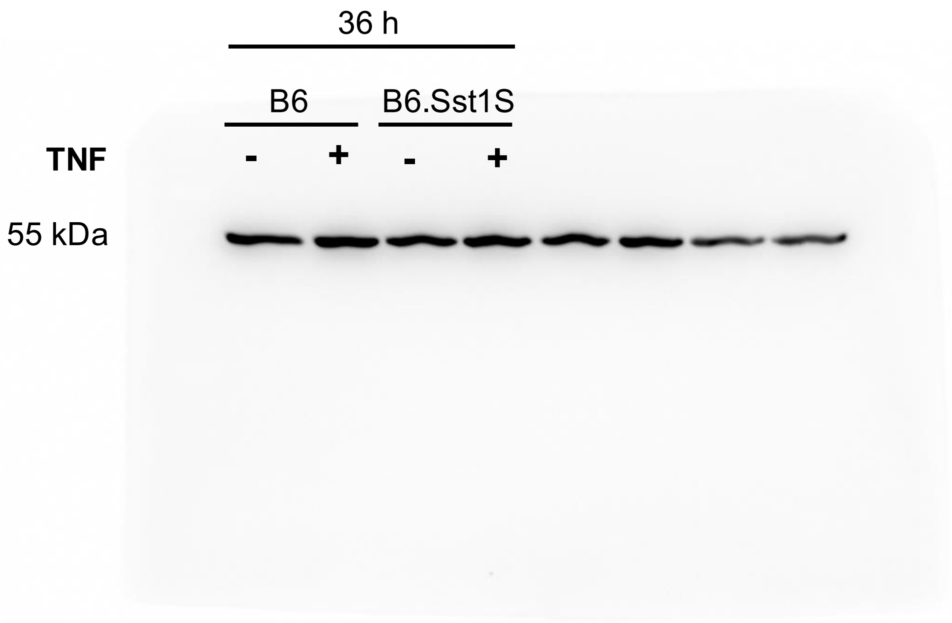

Supplement: Figure 3—source data 1. [file elife-106814-fig3-data1.zip › Figure 3-source data 1.pdf]
