# Supplementary material for: Lipid peroxidation and type I interferon coupling fuels pathogenic macrophage activation causing tuberculosis susceptibility
Source: eLife. 2025 Oct 2;14:RP106814. doi: 10.7554/eLife.106814 (PMC12490860; doi:10.7554/eLife.106814)

Figure 5B: c-Myc- 6 h

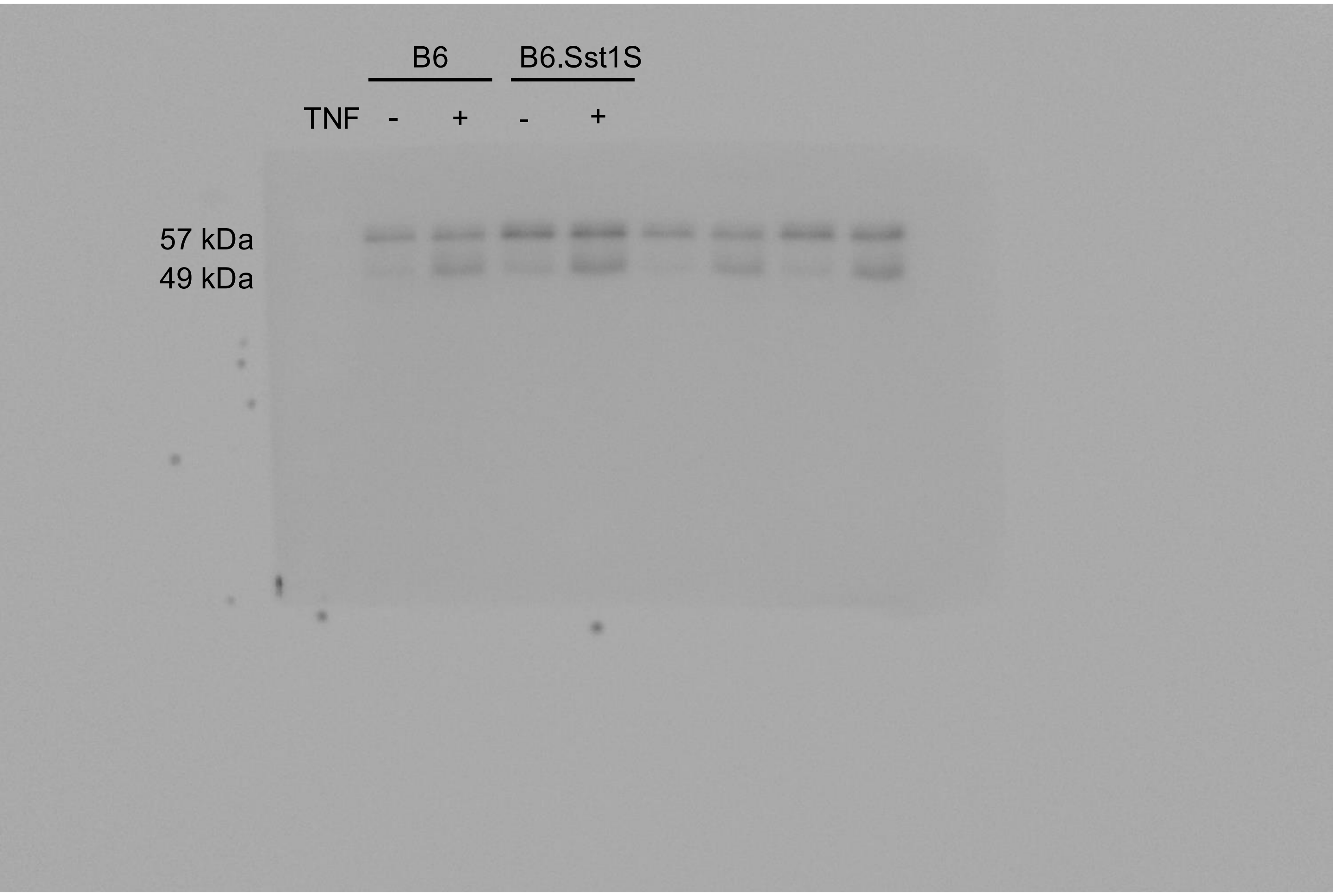

Figure 5B: β-tubulin- 6 h

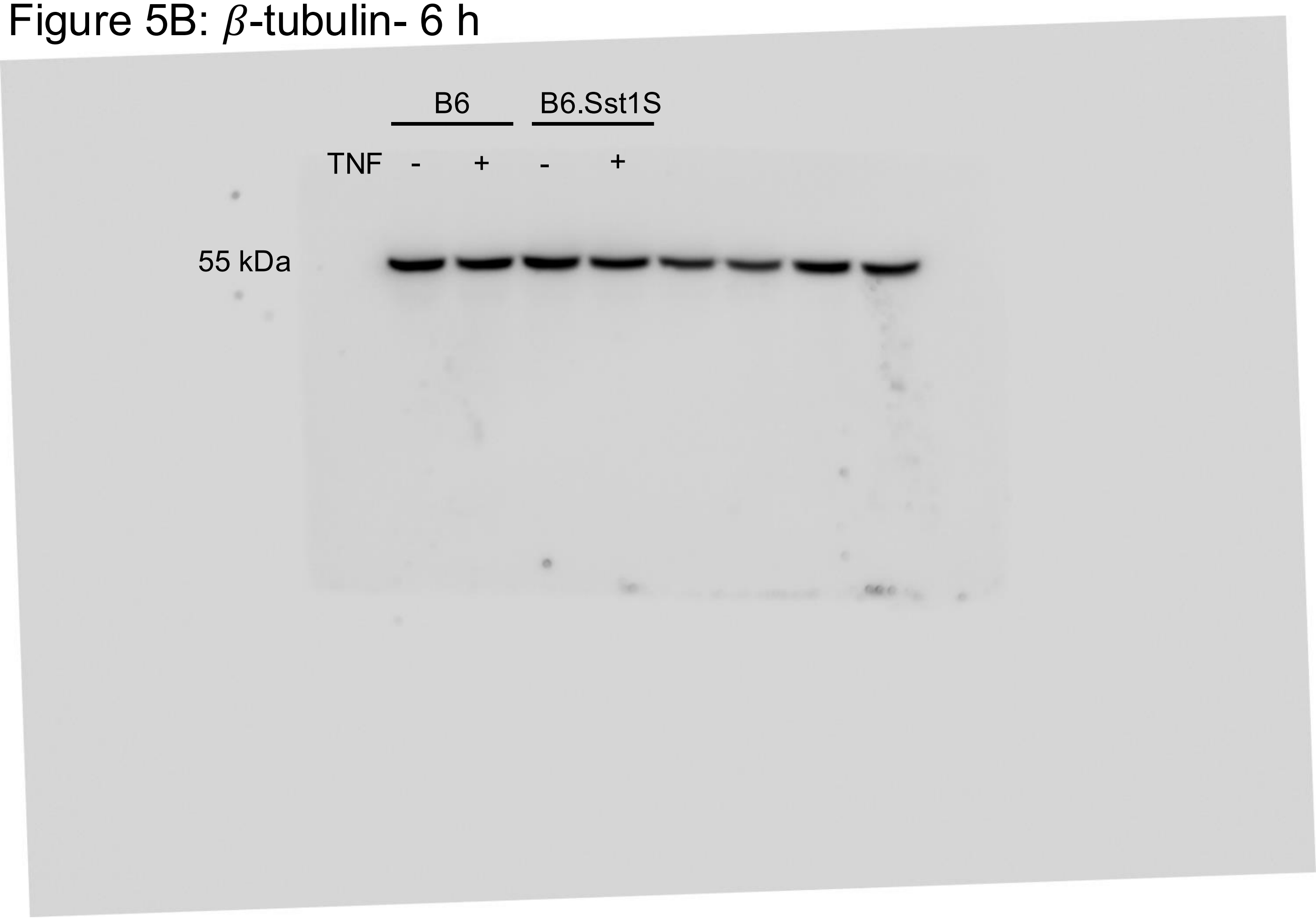

Figure 5B: c-Myc- 12 h

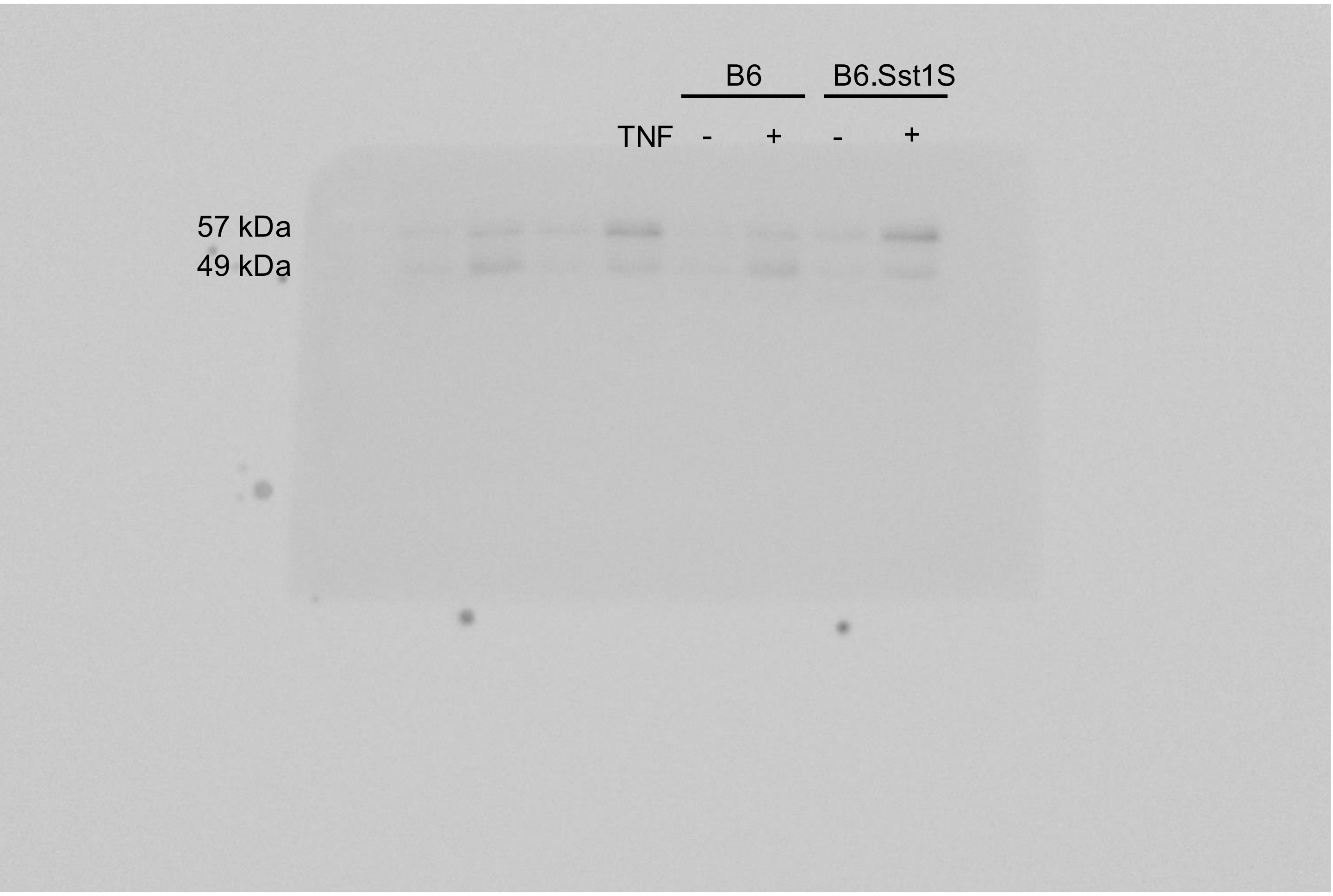

Figure 5B: β-tubulin- 12 h

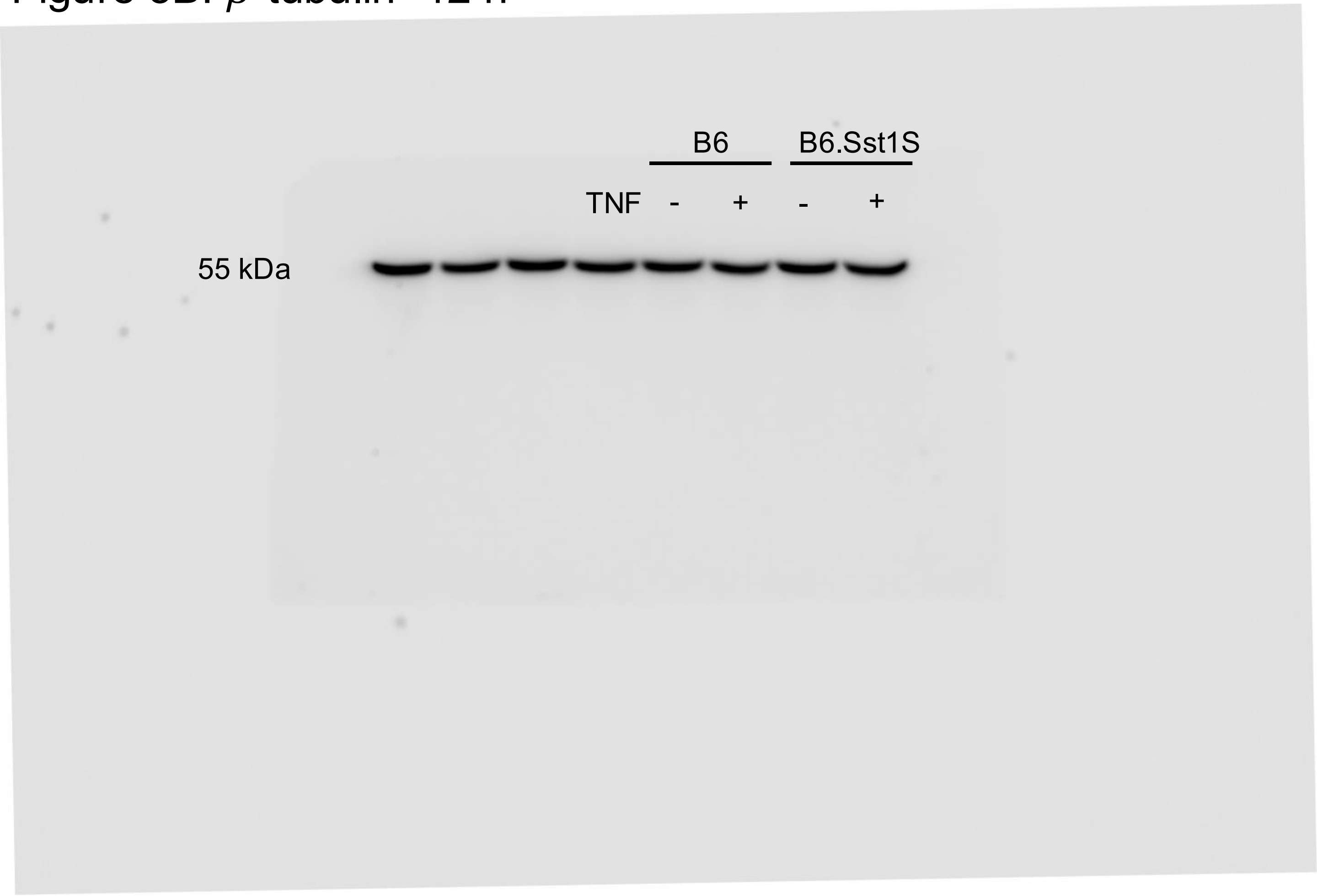

Figure 5C: Fth

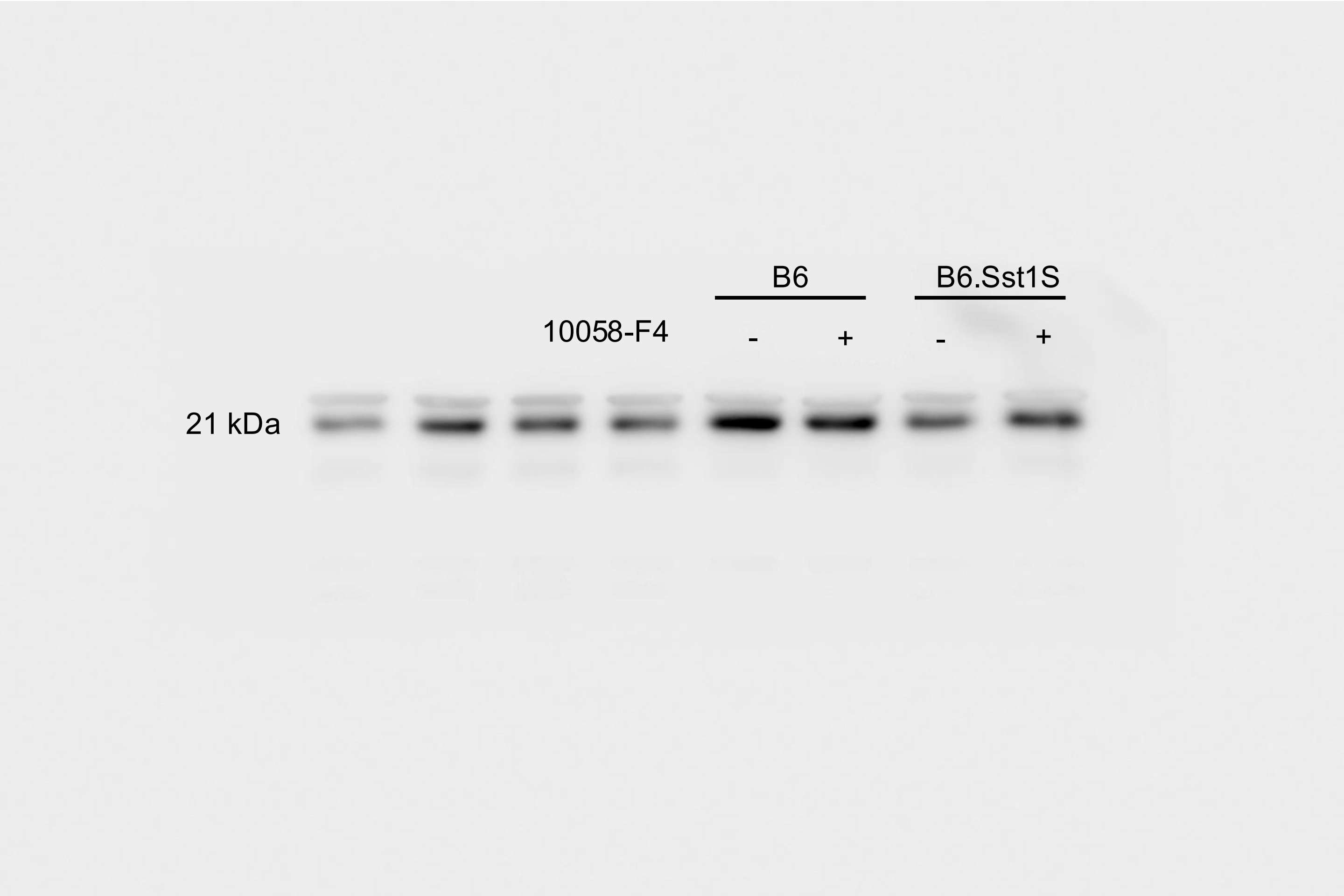

Figure 5C: Ftl

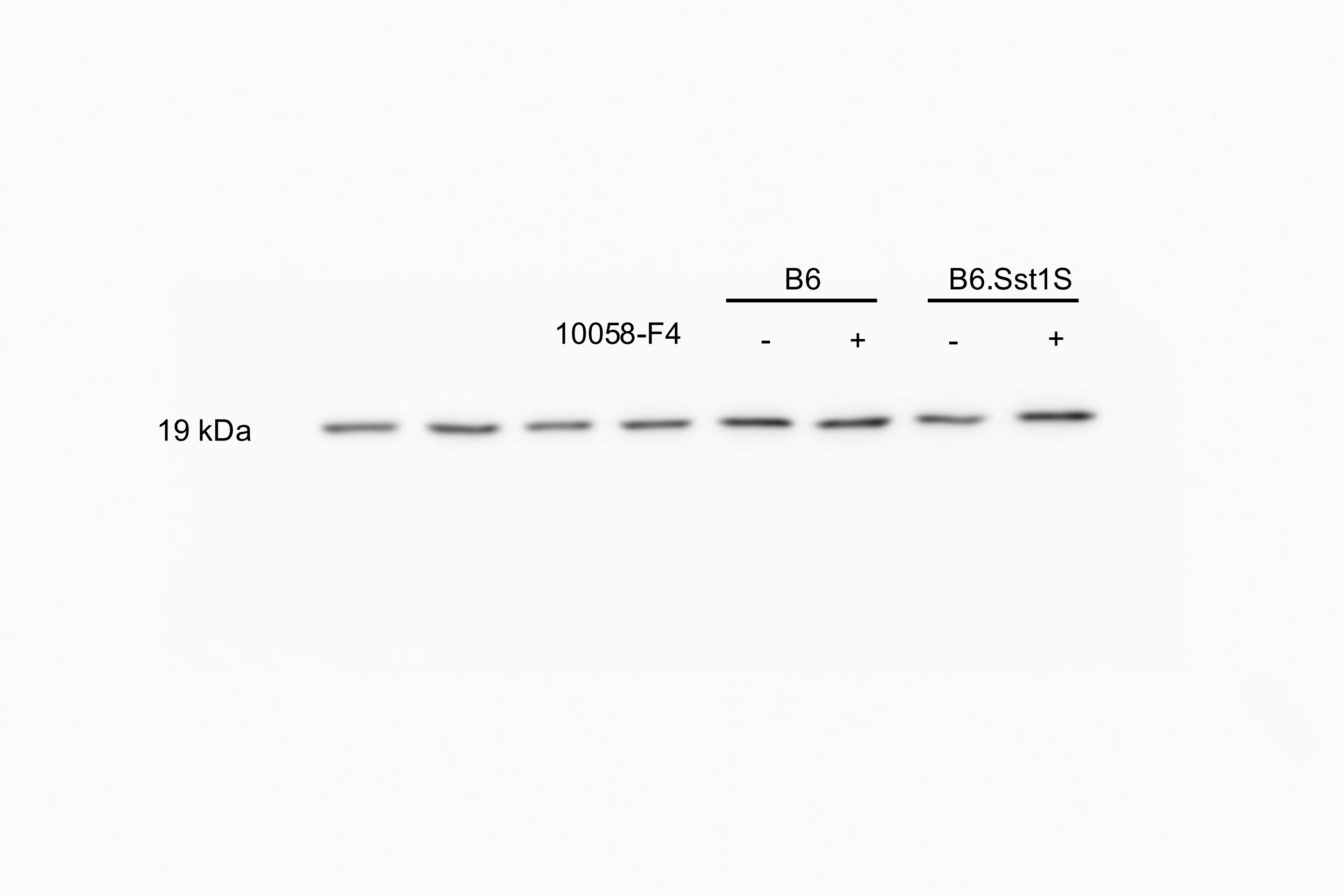

Figure 5C: β-tubulin

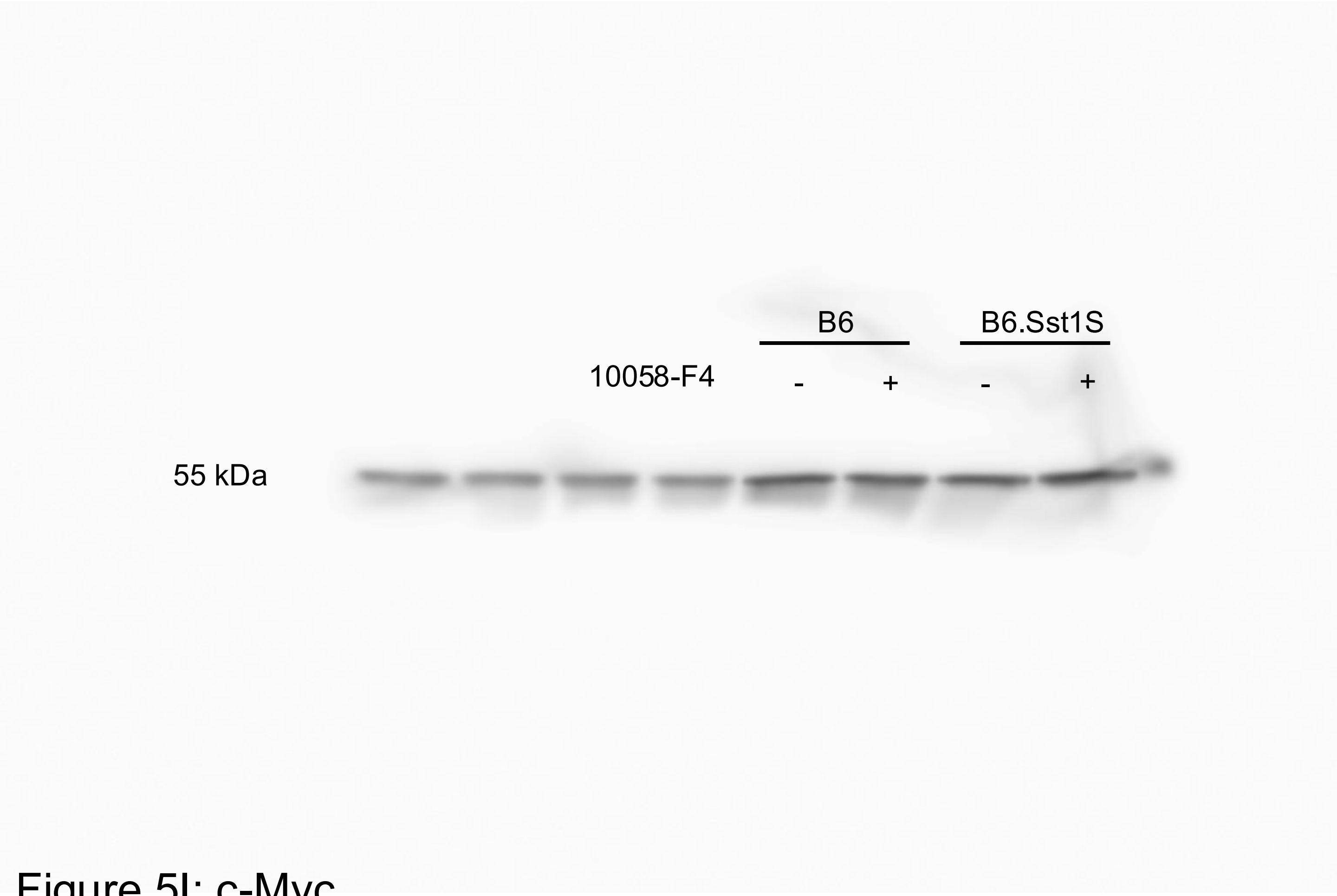

Figure 5I: c-Myc

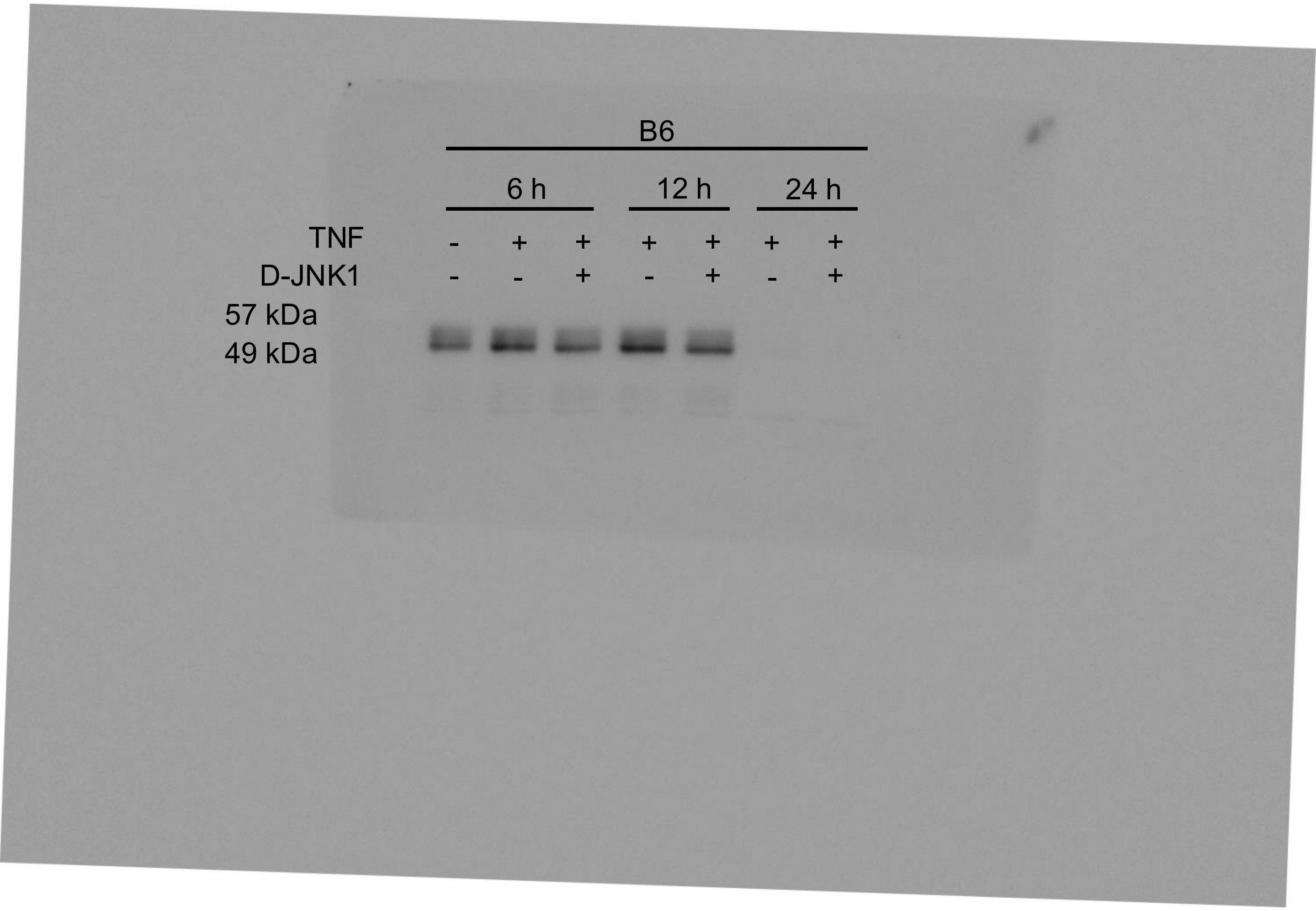

Figure 5I: p-cJun

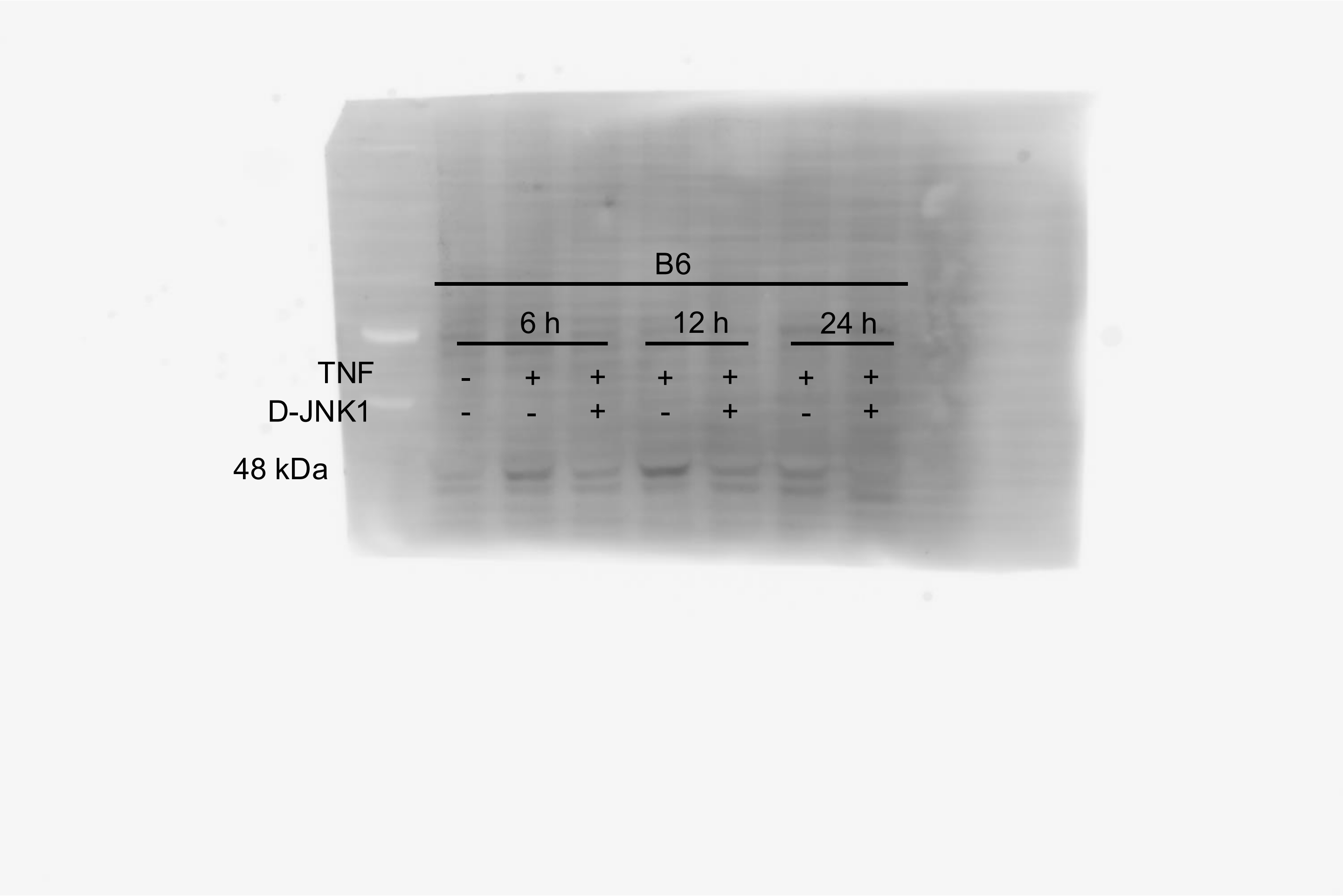

Figure 5I: β-tubulin

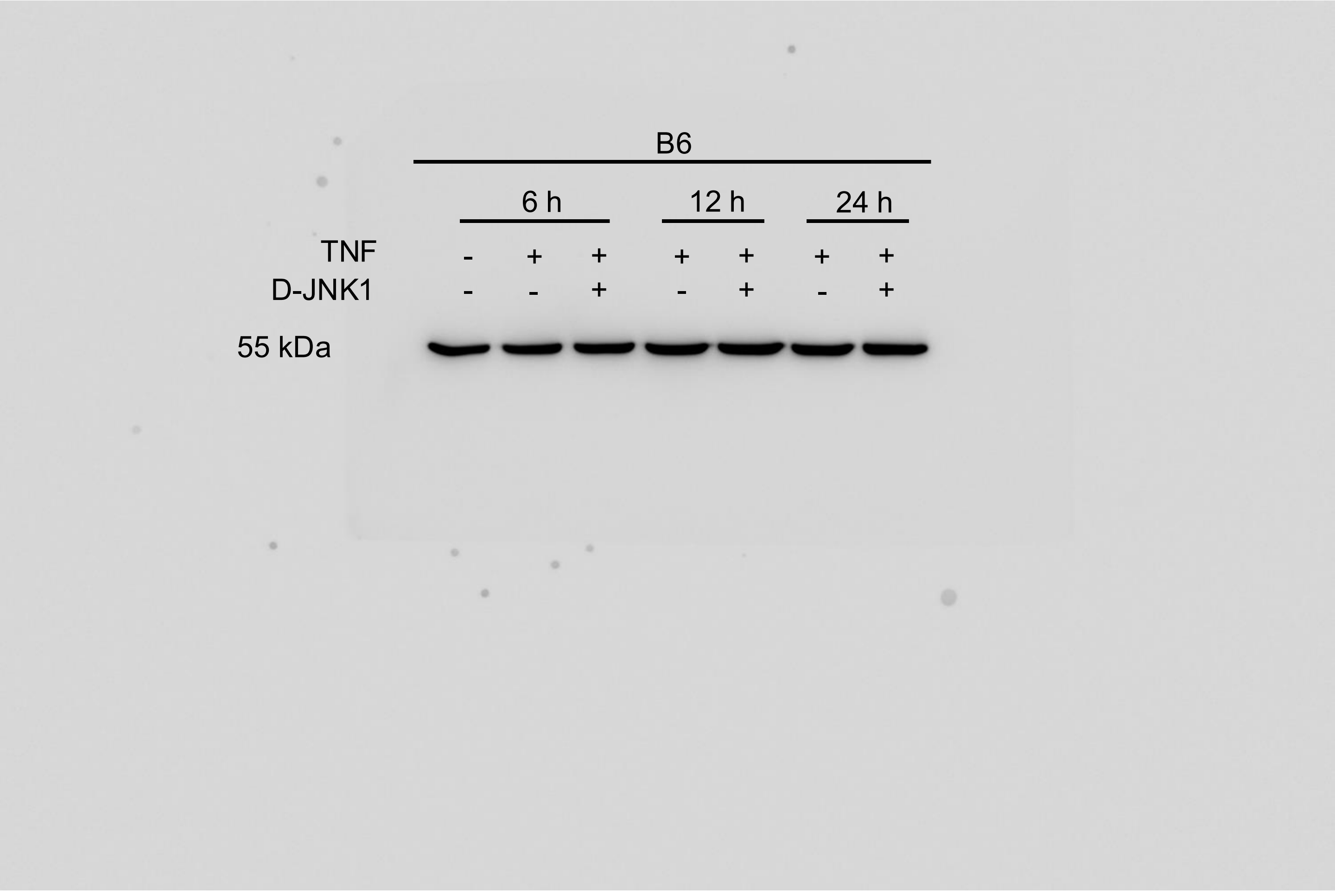

Figure 5J: c-Myc

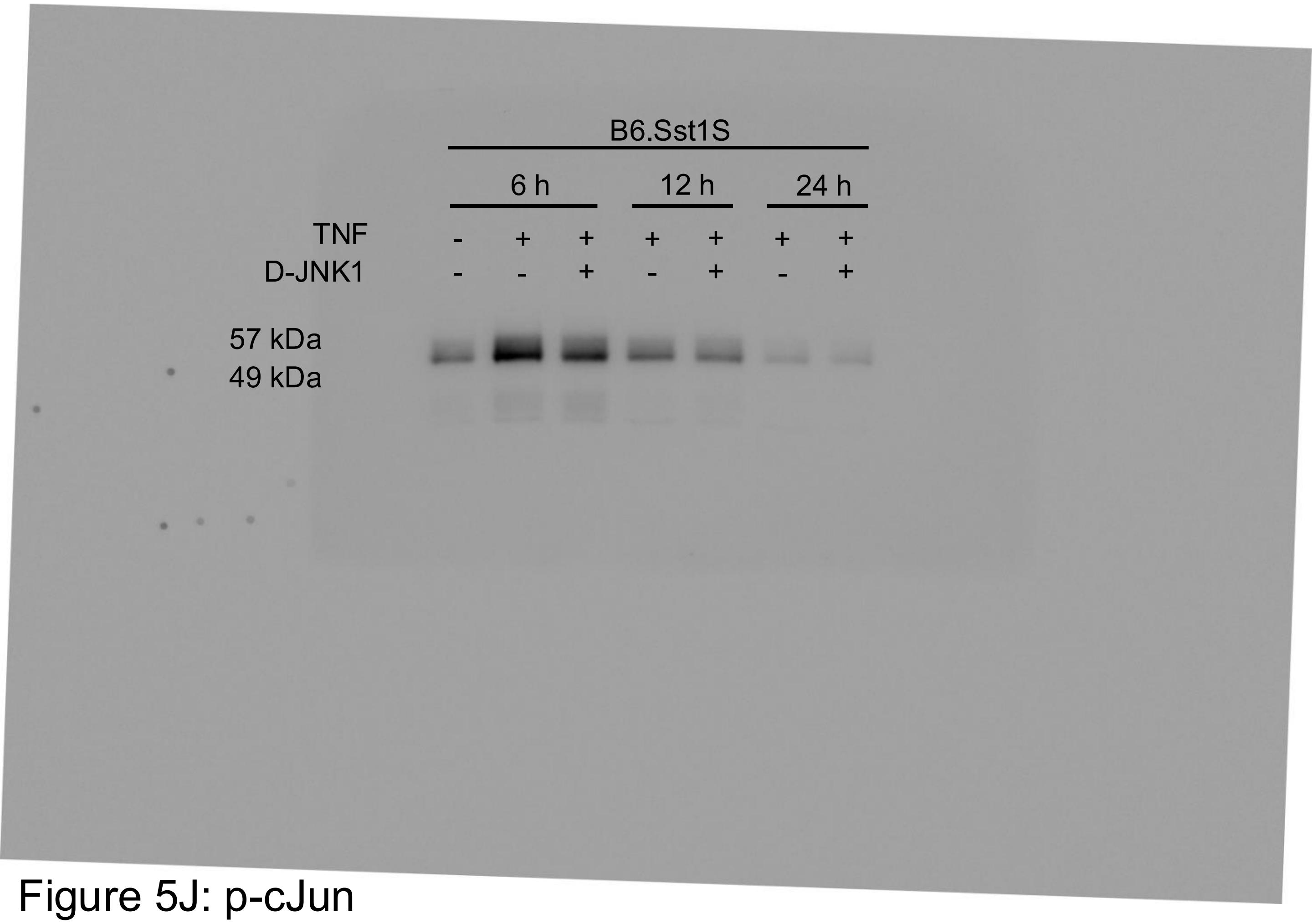

Figure 5J: p-cJun

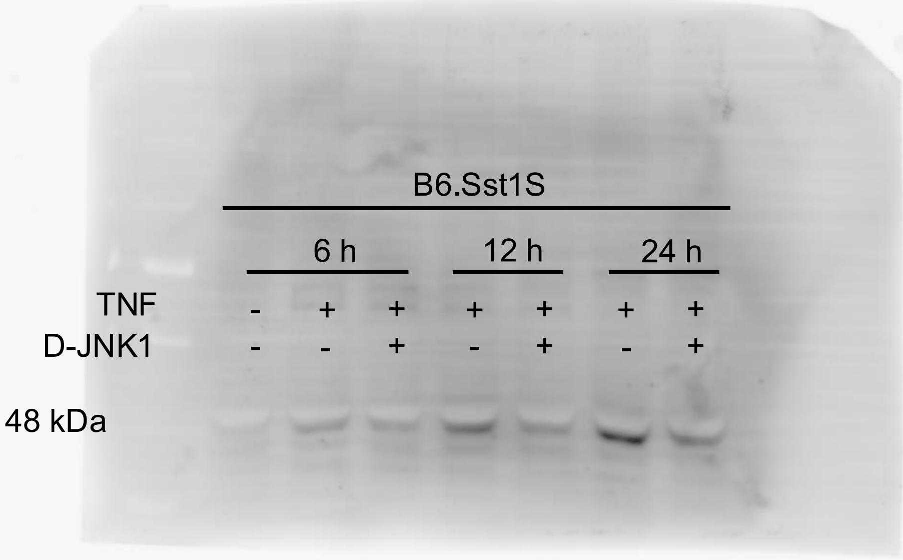

Figure 5J: β-tubulin

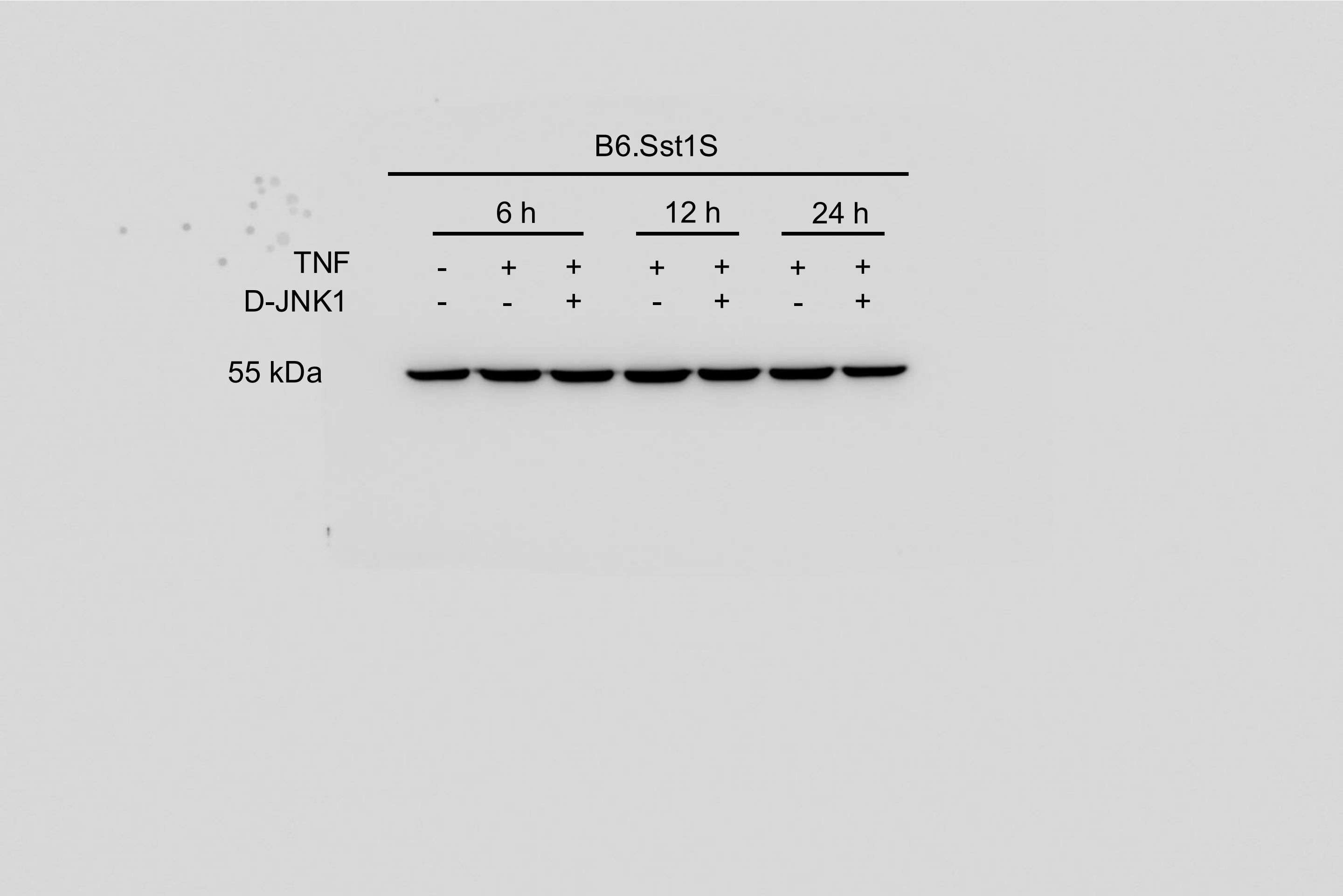

Supplement: Figure 5—source data 1. [file elife-106814-fig5-data1.zip › Figure 5-source data 1.pdf]
