# Supplementary material for: Lipid peroxidation and type I interferon coupling fuels pathogenic macrophage activation causing tuberculosis susceptibility
Source: eLife. 2025 Oct 2;14:RP106814. doi: 10.7554/eLife.106814 (PMC12490860; doi:10.7554/eLife.106814)
Supplement: Supplementary file 1. [file elife-106814-supp1.docx]

**Supplementary File 1. Cell cycle analysis of B6 and B6.Sst1S BMDMS 24 h after TNF stimulation using scRNA-seq.**

| **Sample** | **G1** | **S** | **G2M** | **G1/S** |
| --- | --- | --- | --- | --- |
| **R** | 3257 | 299 | 507 | 10.9 |
| **S** | 3073 | 437 | 540 | 7 |
| **RT** | 1877 | 638 | 507 | 2.9 |
| **ST** | 2049 | 1107 | 367 | 1.9 |
