# Supplementary material for: Lipid peroxidation and type I interferon coupling fuels pathogenic macrophage activation causing tuberculosis susceptibility
Source: eLife. 2025 Oct 2;14:RP106814. doi: 10.7554/eLife.106814 (PMC12490860; doi:10.7554/eLife.106814)
Supplement: Supplementary file 2. [file elife-106814-supp2.docx]

**Supplementary file 2. Cell cycle analysis of B6 and B6.Sst1S specific BMDM subpopulations 24 h after TNF stimulation using scRNA-seq.**

| **Cluster** | **G1** | **S** | **G2M** | **G1/S** |
| --- | --- | --- | --- | --- |
| 2 | 267 | 573 | 133 | 0.5 |
| **3** | 1627 | 269 | 41 | 6 |
| **4** | 943 | 220 | 17 | 4.3 |
| 5 | 718 | 308 | 29 | 2.3 |
