# Supplementary material for: Lipid peroxidation and type I interferon coupling fuels pathogenic macrophage activation causing tuberculosis susceptibility
Source: eLife. 2025 Oct 2;14:RP106814. doi: 10.7554/eLife.106814 (PMC12490860; doi:10.7554/eLife.106814)
Supplement: Supplementary file 3. [file elife-106814-supp3.docx]

**Supplementary file 3. Gene set enrichment analysis of differentially activated pathways in B6 and B6.Sst1S BMDMs 12 h after TNF stimulation.**

Gene set enrichment analysis results for the top enriched gene sets from the KEGG, MSigDB Hallmark, Reactome databases when comparing B6.sst1S vs B6 BMDMs stimulated with TNF for 12 h. Output includes gene set names and FDR corrected p-values. The analysis was performed using FPKM data and the camera function from the limma R package.

Gene set enrichment analysis results for the top enriched gene sets from the KEGG, MSigDB Hallmark, Reactome databases when comparing B6.Sst1S vs B6 BMDMs stimulated with TNF for 12 h. Output includes gene set names and FDR corrected p-values. The analysis was performed using FPKM data and the camera function from the limma R package.

Gene Set Enrichment Analysis

**
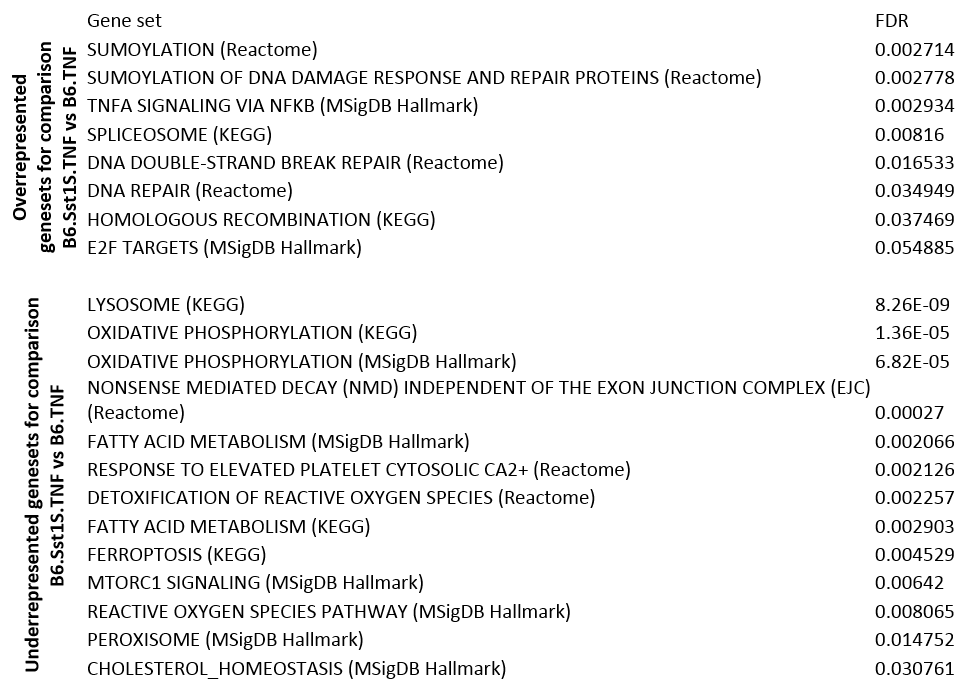
**
