# Supplementary material for: Lipid peroxidation and type I interferon coupling fuels pathogenic macrophage activation causing tuberculosis susceptibility
Source: eLife. 2025 Oct 2;14:RP106814. doi: 10.7554/eLife.106814 (PMC12490860; doi:10.7554/eLife.106814)

**Supplementary file 4.** **Transcription factor binding sites analysis of differentially expressed genes in B6 and B6.Sst1S BMDMs 12 h after TNF stimulation.**

The functional profiling and transcription factor binding sites analysis (TFBs) was performed for two sets of genes which represent B6.Sst1S and B6 BMDMs responses to TNF.


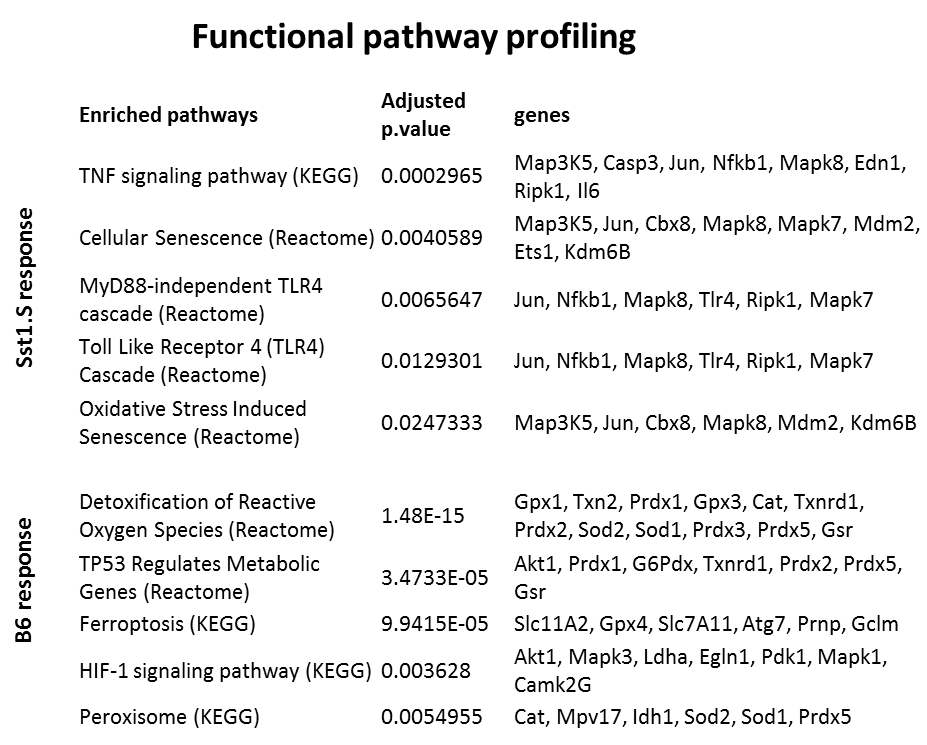

Supplement: Supplementary file 4. [file elife-106814-supp4.docx]
