# Supplementary material for: Lipid peroxidation and type I interferon coupling fuels pathogenic macrophage activation causing tuberculosis susceptibility
Source: eLife. 2025 Oct 2;14:RP106814. doi: 10.7554/eLife.106814 (PMC12490860; doi:10.7554/eLife.106814)

**Supplementary file 5. The list of identified transcription factors associated with differences between activated genes in response to TNF stimulation in B6 and B6.Sst1S BMDMs.**


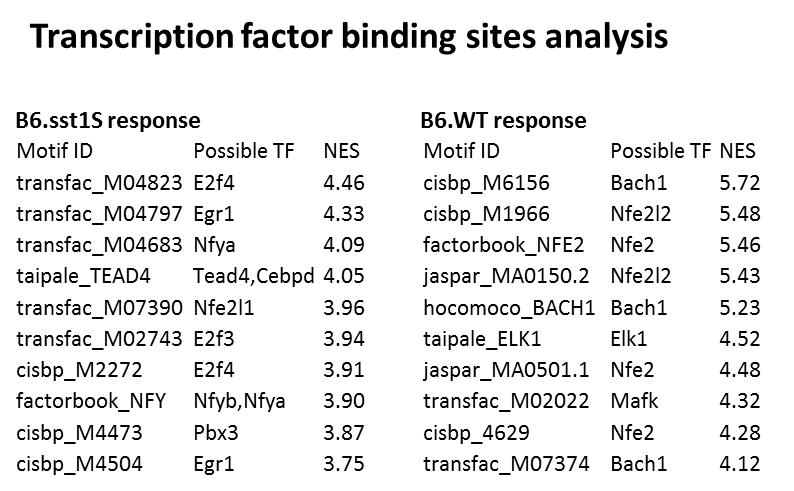

Supplement: Supplementary file 5. [file elife-106814-supp5.docx]
