# Supplementary material for: Lipid peroxidation and type I interferon coupling fuels pathogenic macrophage activation causing tuberculosis susceptibility
Source: eLife. 2025 Oct 2;14:RP106814. doi: 10.7554/eLife.106814 (PMC12490860; doi:10.7554/eLife.106814)

**Supplementary file 6. Master regulator analysis of the transcription factors associated with differences between activated genes in response to TNF stimulation in B6 and B6.Sst1S BMDMs.**

Master regulator analysis was performed using Virtual Inference of Protein Activity by Enriched Regulon Analysis (VIPER) algorithm, the mice macrophage gene regulatory network and log2 transformed FPKM values of genes from gene ontology category GO: 0006979. The table shows the identified list of transcription factors associated with differences between activated genes in response to TNF stimulation in B6.Sst1S vs B6 BMDMs.


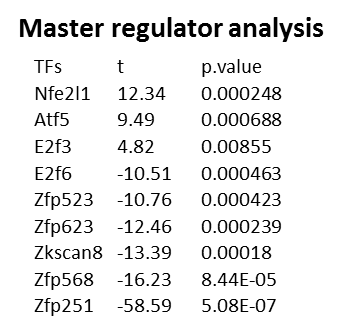

Supplement: Supplementary file 6. [file elife-106814-supp6.docx]
