# Supplementary material for: Lipid peroxidation and type I interferon coupling fuels pathogenic macrophage activation causing tuberculosis susceptibility
Source: eLife. 2025 Oct 2;14:RP106814. doi: 10.7554/eLife.106814 (PMC12490860; doi:10.7554/eLife.106814)
Supplement: MDAR checklist [file elife-106814-mdarchecklist1.docx]

**Materials Design Analysis Reporting (MDAR)**

**Checklist for Authors**

The [MDAR framework](https://osf.io/xfpn4/) establishes a minimum set of requirements in transparent reporting mainly applicable to studies in the life sciences.

For all that apply, please note **where in the article** the information is provided. Please note that we also collect information about data availability and ethics in the submission form.

**Materials:**

| **Newly created materials** | **Indicate where provided: section/figure legend** | **N/A** |
| --- | --- | --- |
| The manuscript includes a dedicated "materials availability statement" providing transparent disclosure about availability of newly created materials including details on how materials can be accessed and describing any restrictions on access. | Mouse strains (B6J.C3-*Sst1^C3HeB/Fej^*Krmn, B6.Sst1S,*Ifnb1*-YFP) and all unique/stable reagents generated in this study are available from corresponding author. Mouse strain (B6J.C3-*Sst1^C3HeB/Fej^*Krmn is available from https://www.mmrrc.org (Stock No: 043908-UNC). [materials and Methods: Experimental animals; Materials availability]. Page 18 |  |
|  |  |  |
| **Antibodies** | **Indicate where provided: section/figure legend** | **N/A** |
| For commercial reagents, provide supplier name, catalogue number and [RRID](https://scicrunch.org/resources), if available. | All antibodies (host species, clonality, supplier, catalog, RRID) are listed in the Key Resources Table and methods (page 23, 24 and 25). |  |
|  |  |  |
| **DNA and RNA sequences** | **Indicate where provided: section/figure legend** | **N/A** |
| Short novel DNA or RNA including primers, probes: Sequences should be included or deposited in a public repository. | All primers sequences used in the study are listed in the Key Resources Table. |  |
|  |  |  |
| **Cell materials** | **Indicate where provided: section/figure legend** | **N/A** |
| Cell lines: Provide species information, strain. Provide accession number in repository OR supplier name, catalog number, clone number, OR RRID. |  | X |
| Primary cultures: Provide species, strain, sex of origin, genetic modification status. | The study used primary murine (*Mus musculus*) BMDMs from C57BL/6J and B6.Sst1S mice. The details provided in Materials and Methods: BMDMs culture and Treatment (page 19) |  |
|  |  |  |
| **Experimental animals** | **Indicate where provided: section/figure legend** | **N/A** |
| Laboratory animals or Model organisms: Provide species, strain, sex, age, genetic modification status. Provide accession number in repository OR supplier name, catalog number, clone number, OR RRID. | The details of laboratory animals or Model organisms used in the study are listed in the Key Resources Table and materials and methods section (page 19). |  |
| Animal observed in or captured from the field: Provide species, sex, and age where possible. |  | X |
|  |  |  |
| **Plants and microbes** | **Indicate where provided: section/figure legend** | **N/A** |
| Plants: provide species and strain, ecotype and cultivar where relevant, unique accession number if available, and source (including location for collected wild specimens). |  | X |
| Microbes: provide species and strain, unique accession number if available, and source. | The following M. tuberculosis strains were used, *Mycobacterium tuberculosis* H37Rv (ATCC, Cat#27294), *Mycobacterium bovis* BCG (ATCC, Cat#35737) and *M. tuberculosis* Erdman(SSB-GFP, *smyc*′::mCherry)(a gift from Shumin Tan).  The details are listed in materials and methods (Infection of BMDM with *M. tuberculosis*, and Infection of mice with *Mycobacterium tuberculosis* and collection of organs) and in the Key Resources Table.  IBC protocol: 25-875 (page 19) |  |
|  |  |  |
| **Human research participants** | **Indicate where provided: section/figure legend) or state if these demographics were not collected** | **N/A** |
| If collected and within the bounds of privacy constraints report on age, sex, gender and ethnicity for all study participants. | Participant demographics including age, sex, body mass index (BMI), diabetes status, alcohol use, and tobacco use were recorded and matched for Blood collection. Blood samples were collected from 41 individuals and recently (<90 days) diagnosed with TB, within one week of treatment commencement from five clinical sites within the Regional Prospective Observational Research for Tuberculosis (RePORT)-India consortium. Individuals for this study were newly diagnosed with TB (within 90 days; sputum smear-positive or Xpert MTB/RIF assay positive (Cepheid, Sunnyvale, CA, USA)), at least 15 years of age, multi-drug-resistant negative, and had received less than one week of treatment. Sociodemographic data such as age, sex, behavioral characteristics (smoking, alcohol use (using the Alcohol Use Disorders Identification Test [AUDIT-C] ), tobacco use, and body mass index (BMI) were obtained through a questionnaire and the medical history of the participants. There were no significant differences in risk factors for TB or treatment failure, including sex, risky alcohol use, tobacco use, BMI, or diabetes. Patients were monitored for two years during and post-treatment with Rifampicin, isoniazid, ethambutol, and pyrazinamide, per India national guidelines [Division CT. Technical and Operational Guidelines for TB Control in India 2016. Central TB Division, Directorate General of Health Service, Ministry of Health and Family Welfare; Government of India, New Delhi. 2016 (Bush et al, 1998). Individuals who failed treatment (n=21) were identified by positive sputum culture or clinical diagnosis of symptoms at any time after 4 full months of treatment and symptoms determined to not be from another cause. Treatment controls were determined by those who remained culture-negative and symptom-negative for the two-year observation period. |  |

**Design:**

| **Study protocol** | **Indicate where provided: section/figure legend** | **N/A** |
| --- | --- | --- |
| If the study protocol has been pre-registered, provide DOI. For clinical trials, provide the trial registration number OR cite DOI. |  | X |
|  |  |  |
| **Laboratory protocol** | **Indicate where provided: section/figure legend** | **N/A** |
| Provide DOI OR other citation details if detailed step-by-step protocols are available. | IACUC: PROTO201800218 (page 19)  IBC: 25-875 (page 19)  <https://doi.org/10.1016/j.xpro.2025.103984>  <https://doi.org/10.1016/j.xpro.2022.101241>  <https://doi.org/10.1016/j.xpro.2025.103640> |  |
|  |  |  |
| **Experimental study design (statistics details) *** | | |
| **For in vivo studies: State whether and how the following have been done** | **Indicate where provided: section/figure legend. If it could have been done, but was not, write “not done”** | **N/A** |
| Sample size determination | Sample sizes were chosen based on prior studies using the same infection model; no formal power calculation was performed. Animals were randomly assigned to experimental groups, and Blinding was applied during data analysis where feasible. Predefined criteria were applied and are reported. |  |
| Randomisation | Animals were randomly assigned to experimental groups. |  |
| Blinding | Qualitative histopathology analysis and semi-quantitative bacterial load analysis were conducted by a board-certified veterinary pathologist in a blinded manner (page 24). |  |
| Inclusion/exclusion criteria |  | X |
|  |  |  |
| **Sample definition and in-laboratory replication** | **Indicate where provided: section/figure legend** | **N/A** |
| State number of times the experiment was replicated in the laboratory. | The experiments were repeated at least 2-4 times. The details are listed in figure legends of respective figure. |  |
| Define whether data describe technical or biological replicates. | The biological replicates were performed at least 2-4 times. The details of each experiment are presented in figure legends of respective figure. |  |
|  |  |  |
| **Ethics** | **Indicate where provided: section/submission form** | **N/A** |
| Studies involving human participants: State details of authority granting ethics approval (IRB or equivalent committee(s), provide reference number for approval. | Ethics approval for the study was obtained from the Institutional Ethics Committee of the participating institutions, and written informed consent was obtained prior to enrollment. This study utilized data from four longitudinal observational studies collected at five clinical sites within the Regional Prospective Observational Research for Tuberculosis (RePORT)-India consortium: Byramjee Jeejeebhoy Government Medical College (BJMC), Pune; the Jawaharlal Institute of Postgraduate Medical Education and Research (JIPMER, Puducherry); National Institute for Research in Tuberculosis (NIRT), Chennai; Prof. M. Viswanathan Diabetes Research Centre (MVDRC), Chennai; and the Christian Medical College (CMC), Vellore(Ayiraveetil et al, 2020; Christopher et al, 2021; Gupte et al, 2016; Kornfeld et al, 2016). |  |
| Studies involving experimental animals: State details of authority granting ethics approval (IRB or equivalent committee(s), provide reference number for approval. | Animal experiments approved by IACUC (PROTO201800218) (page 19). |  |
| Studies involving specimen and field samples: State if relevant permits obtained, provide details of authority approving study; if none were required, explain why. | Data used and presented in this work were re-analyses or secondary analyses from a parent study. The samples and data were generated independently of this study, so no new samples were collected or sequenced for this study. For the parent study, ethics approval for the study was obtained from the Institutional Ethics Committee of the participating institutions, and written informed consent was obtained prior to enrollment. This study utilized data from four longitudinal observational studies collected at five clinical sites within the Regional Prospective Observational Research for Tuberculosis (RePORT)-India consortium: Byramjee Jeejeebhoy Government Medical College (BJMC), Pune; the Jawaharlal Institute of Postgraduate Medical Education and Research (JIPMER, Puducherry); National Institute for Research in Tuberculosis (NIRT), Chennai; Prof. M. Viswanathan Diabetes Research Centre (MVDRC), Chennai; and the Christian Medical College (CMC), Vellore. |  |
|  |  |  |
| **Dual Use Research of Concern (DURC)** | **Indicate where provided: section/submission form** | **N/A** |
| If study is subject to dual use research of concern regulations, state the authority granting approval and reference number for the regulatory approval. |  | X |

**Analysis:**

| **Attrition** | **Indicate where provided: section/figure legend** | **N/A** |
| --- | --- | --- |
| Describe whether exclusion criteria were pre-established. Report if sample or data points were omitted from analysis. If yes, report if this was due to attrition or intentional exclusion and provide justification. |  | X |
|  |  |  |
| **Statistics** | **Indicate where provided: section/figure legend** | **N/A** |
| Describe statistical tests used and justify choice of tests. | Statistical analyses were performed using GraphPad Prism 9 software (RRID:SCR_002798). Differences among groups involving two or more variables were assessed using two-way analysis of variance (ANOVA) with adjustments for multiple post hoc comparisons. For comparisons across multiple groups based on a single variable, one-way ANOVA with post hoc testing was applied. Two-tailed paired or unpaired t-tests were used for comparisons between two groups after verifying data normality. For non-parametric datasets, the Wilcoxon Rank Sum test (Mann-Whitney U test equivalent) was employed. Additional details of statistical tests used are provided in figure legends and materials and method: statistical analysis (page 19). |  |
|  |  |  |
| **Data availability** | **Indicate where provided: section/submission form** | **N/A** |
| For newly created and reused datasets, the manuscript includes a data availability statement that provides details for access (or notes restrictions on access). | The RNA-seq and spatial transcriptomics datasets generated in this study have been deposited in GEO under accession numbers GSE164698 and GSE292392 (page 18). |  |
| When newly created datasets are publicly available, provide accession number in repository OR DOI and licensing details where available. | The RNA-seq and spatial transcriptomics datasets generated in this study have been deposited in GEO under accession numbers GSE164698 and GSE292392 (page 18). |  |
| If reused data is publicly available provide accession number in repository OR DOI, OR URL, OR citation. |  | X |
|  |  |  |
| **Code availability** | **Indicate where provided: section/figure legend** | **N/A** |
| For any computer code/software/mathematical algorithms essential for replicating the main findings of the study, whether newly generated or re-used, the manuscript includes a data availability statement that provides details for access or notes restrictions. | . | X |
| Where newly generated code is publicly available, provide accession number in repository, OR DOI OR URL and licensing details where available. State any restrictions on code availability or accessibility. |  | X |
| If reused code is publicly available provide accession number in repository OR DOI OR URL, OR citation. |  | X |

**Reporting:**

The MDAR framework recommends adoption of discipline-specific guidelines, established and endorsed through community initiatives.

| **Adherence to community standards** | **Indicate where provided: section/figure legend** | **N/A** |
| --- | --- | --- |
| State if relevant guidelines (e.g., ICMJE, MIBBI, ARRIVE, STRANGE) have been followed, and whether a checklist (e.g., CONSORT, PRISMA, ARRIVE) is provided with the manuscript. | Reporting follows ARRIVE and MDAR guidance. All the details are provided in Key Resources Table and in Materials and Methods section. |  |
